# Global Burden and Trends of Primary Liver Cancer Attributable to Comorbid Type 2 Diabetes Mellitus Among People Living with Hepatitis B: An Observational Trend Study from 1990 to 2019

**DOI:** 10.1007/s44197-024-00237-1

**Published:** 2024-05-07

**Authors:** Jinzhao Xie, Xiao Lin, Xiaoyan Fan, Xu Wang, Deng Pan, Jinghua Li, Yuantao Hao, Yusheng Jie, Lei Zhang, Jing Gu

**Affiliations:** 1 Department of Medical Statistics, School of Public Health, Sun Yat-sen University, Guangzhou, China; 2 School of Public Health, LKS Faculty of Medicine, The University of Hong Kong, Pokfulam, Hong Kong SAR, China; 3 Sun Yat-sen Global Health Institute, School of Public Health and Institute of State Governance, Sun Yat-sen University, Guangzhou, China; 4 Key Laboratory of Health Informatics of Guangdong Province, Sun Yat-sen University, Guangzhou, China; 5 Center for Public Health and Epidemic Preparedness and Response, Peking University, Beijing, China; 6 Department of Infectious Diseases, The Third Affiliated Hospital, Sun Yat-sen University, Guangzhou, China; 7 China-Australia Joint Research Centre for Infectious Diseases, School of Public Health, Xi’an Jiaotong University Health Science Centre, Xi’an, China; 8 Central Clinical School, Faculty of Medicine, Monash University, Melbourne, VIC Australia; 9 Artificial Intelligence and Modelling in Epidemiology Program, Melbourne Sexual Health Centre, Alfred Health, Melbourne, VIC Australia; 10 Department of Epidemiology and Biostatistics, College of Public Health, Zhengzhou University, Zhengzhou, China

**Keywords:** Hepatitis B virus, Diabetes Mellitus, Global Burden of Disease, Liver neoplasms, Prevalence, Disability-adjusted life years

## Abstract

**Background:**

Type 2 diabetes mellitus (T2DM) increases the risk of liver cancer among people living with hepatitis B virus (HBV). Our study aimed to estimate the global burden and trends of liver cancer attributable to comorbid T2DM among people living with HBV from 1990 to 2019.

**Methods:**

We calculated the population attributable fractions (PAFs) of liver cancer attributable to comorbid T2DM among the burden of HBV-related liver cancer. We applied the PAFs to the burden of HBV-related liver cancer derived from the Global Burden of Disease (GBD) 2019 database to obtain the burden of liver cancer attributable to HBV–T2DM comorbidity. The prevalence, disability-adjusted life year (DALY), and deaths of liver cancer attributable to the comorbidity were assessed at the global, regional, and country levels and then stratified by the sociodemographic index (SDI), sex, and age group. Estimated annual percentage changes (EAPCs) were calculated to quantify the temporal trends.

**Results:**

In 2019, the global age-standardized prevalence and DALY rates of liver cancer attributable to HBV–T2DM comorbidity were 9.9 (8.4–11.5) and 182.4 (154.9–212.7) per 10,000,000 individuals, respectively. High-income Asia Pacific and East Asia had the highest age-standardized prevalence and DALY rates of liver cancer attributable to HBV–T2DM comorbidity, respectively. From 1990 to 2019, age-standardized prevalence and DALY rates increased in 16 out of 21 GBD regions. High-income North America had the largest annual increases in both age-standardized prevalence rates (EAPC = 6.07; 95% UI, 5.59 to 6.56) and DALY rates (EAPC = 4.77; 95% UI, 4.35 to 5.20), followed by Australasia and Central Asia. Across all SDI regions, the high SDI region exhibited the most rapid increase in age-standardized prevalence and DALY rates from 1990 to 2019. Additionally, men had consistently higher disease burdens than women across all age groups. The patterns of mortality burden and trends are similar to those of DALYs.

**Conclusions:**

The burden of liver cancer attributable to comorbid T2DM among people living with HBV has exhibited an increasing trend across most regions over the last three decades. Tailored prevention strategies targeting T2DM should be implemented among individuals living with HBV.

**Supplementary Information:**

The online version contains supplementary material available at 10.1007/s44197-024-00237-1.

## Introduction

Liver cancer is the fourth leading cause of cancer-related deaths worldwide [1]. In 2019, approximately 50% of all reported cases of hepatocellular carcinoma (HCC) globally were attributed to hepatitis B virus (HBV) infection [2, 3]. In certain regions, such as East Asia and Africa, this percentage can be even higher [2]. Despite the effective implementation of global hepatitis B vaccine strategies [4, 5], there are still approximately 300 million individuals worldwide living with chronic HBV [6]. These individuals are gradually aging, making them more susceptible to comorbid noncommunicable diseases (NCDs), such as type 2 diabetes mellitus (T2DM) [7–10]. Over the last three decades, the prevalence of T2DM among the HBV-infected population in the Western Pacific region has increased by 161.07% [10]. Moreover, T2DM is also one of the most common NCDs coexisting with HBV infection in North America [9, 11].

T2DM and HBV infection contribute to the development of each other. HBV infection can cause liver damage and persistent inflammatory responses, leading to a glycometabolic disorder [12, 13]. A previous meta-analysis estimated that the odds ratio for the prevalence of T2DM between HBV-infected individuals and those without HBV infection was 1.33 (95% confidence interval [CI] 1.09 to 1.62) [14]. Additionally, patients with T2DM, due to their frequent percutaneous exposure to blood, are at an increased risk of HBV infection [15]. The literature indicates that patients with T2DM have a 60% higher seroprevalence of antibodies to hepatitis B core antigen, indicating the possibility of past or present HBV infection, compared to individuals without T2DM [16]. The global burden of the comorbidity between T2DM and HBV may increase, owing to synergistic interactions between the two diseases, coupled with the rising burden of T2DM.

T2DM has been demonstrated to be an independent risk factor for liver cancer [17, 18], which may be due to hyperglycemia, hyperinsulinemia, insulin resistance, and enhanced inflammatory processes caused by T2DM [19]. For individuals with HBV infection, T2DM increases the risk of liver cancer. The accumulation of plasma free fatty acids, along with oxidative stress and inflammation resulting from T2DM, may further elevate the risk of cirrhosis and liver cancer caused by HBV infection [20–22]. Epidemiological evidence also reveals that T2DM increases the risk of liver cancer among individuals infected with HBV. A meta-analysis of 19 cohort studies indicated that the hazard ratio of developing liver cancer between patients with HBV-T2DM comorbidity and patients with HBV infection alone was 1.36 (95% CI 1.23 to 1.49) [23].

Given the large number of individuals with HBV infection who are rapidly aging and the increasing global burden of T2DM, the future burden of liver cancer attributable to comorbid T2DM in HBV-infected individuals may escalate [7, 24]. Although several previous studies have assessed the burden of liver cancer among individuals with HBV infection, none have considered the impact of comorbid T2DM [3, 25]. It is essential to estimate the disease burden caused by T2DM in HBV-infected individuals across different regions to gain valuable insights into prognosis factors. Such estimations can help design tailored intervention strategies to reduce the disease burden for those affected by HBV–T2DM comorbidity. However, currently, there is a lack of studies comprehensively estimating the global burden and trends of liver cancer attributable to comorbid T2DM in HBV-infected individuals.

Therefore, our aim is to estimate the global disease burden of liver cancer attributable to HBV–T2DM comorbidity among people living with HBV based on the Global Burden of Disease (GBD) 2019 database. We calculated the global, regional, and country-level prevalence and disability-adjusted life years (DALYs) of liver cancer due to the comorbidity and analyzed the trends in disease burden from 1990 to 2019. Additionally, we reported the disease burden and trends stratified according to the sociodemographic index (SDI), sex, and age. This study was conducted following the Guidelines for Accurate and Transparent Health Estimates Reporting [26].

## Materials and Methods

### Data Source

Our data were obtained from the GBD 2019 database, which is constructed and maintained by the Institute for Health Metrics and Evaluation. The GBD database contains data on the global burden of 369 diseases and injuries across 204 countries and territories from 1990 to 2019 [27]. We extracted the annual prevalence, DALYs, and deaths of T2DM and liver cancer due to HBV for the years 1990–2019 using the Global Health Data Exchange query tool (https://ghdx.healthdata.org/gbd-2019). The definitions for HBV infection, T2DM, and primary liver cancer, along with the search strategy for the GBD database, are detailed in the Supplemental Methods section of the Supplementary Material. The 204 countries in the GBD database were categorized into 21 regions according to their geographic locations [27]. Additionally, SDI, a composite indicator of sociodemographic developmental status, was developed by the GBD team to categorize the 204 countries into five socioeconomic developmental levels, ranging from low to high. The countries included in each SDI category are presented in Table S1. The SDI calculation is based on the average income per capita, educational attainment, and total fertility rate [27]. Data on the prevalence, DALYs, and deaths of liver cancer were available by region, country, SDI, sex, and age. We obtained the prevalence, DALY, and deaths as the total number of cases, rates (i.e., cases per 10,000,000 individuals), and age-standardized rates (ASR). ASRs for prevalence and DALYs were defined as the weighted average of the age-specific rates and used as the primary indicator of disease burden in this study. ASRs allow for comparisons of disease burden across regions with different age structures or within the same region over time [25]. The ASRs in the GBD data were estimated based on the standard world population as per the GBD database. Details of the GBD 2019 and the general methodology can be found in a previous study [27].

### Data Processing and Statistical Analysis

#### Calculating the Population Attributable Fraction of Liver Cancer

We first used Levin’s formula (Eq. 1) to estimate the population attributable fraction (PAF) of liver cancer burden attributable to comorbid T2DM among people living with HBV [28, 29].

1 $$PAF = \frac{{p \times \left( {RR - 1} \right)}}{{p \times \left( {RR - 1} \right) + 1}}$$

In Eq. 1, the variable *RR* represents the relative risk (RR) of developing liver cancer among those with HBV–T2DM comorbidity compared with those with only HBV. We searched Medline and Web of Science to identify an appropriate estimate for the *RR* in Eq. 1. The search strategy and temporal scope are detailed in Supplemental Methods section of the Supplementary Material. A meta-analysis of 19 cohort studies indicated that the hazard ratio of developing liver cancer between patients with HBV-T2DM comorbidity and patients with HBV infection alone was 1.36 (95% CI 1.23 to 1.49) [23]. We used the value of this hazard ratio as the *RR* in Eq. 1 to calculate the PAFs, consistent with methodologies adopted in previous studies [28, 29]. We assumed that the *RR* value in Eq. 1 is equal across countries and years. The variable *p* in Eq. 1 represents the estimated prevalence of comorbid T2DM among individuals with HBV infection. This prevalence was calculated by multiplying the prevalence of T2DM in the general population by the prevalence ratio of T2DM in HBV-infected individuals compared to that of the general population. The prevalence ratio was obtained from a large-scale meta-analysis (pooled prevalence ratio: 1.33, 95% CI 1.09 to 1.62) [14]. We assumed that the prevalence ratio is constant across countries, years, and age groups. The prevalence of T2DM in the general population of different countries, years, and age groups was obtained from the GBD 2019 database. The PAFs are specific to time, region, sex, and age. PAFs ranged from 0.73 to 4.78% across the 21 GBD regions in 2019. PAFs for different regions, countries, SDI levels, sex, and age groups are presented in Table S2 and Figure S1.

#### Analyzing the Global Burden and Trends of Liver Cancer Due to Comorbid T2DM Among People Living with HBV

We estimated the burden of liver cancer attributable to comorbid T2DM among people living with HBV by multiplying the PAFs by the disease burdens (i.e., prevalence, DALY, and death) of liver cancer related to HBV [29]. We examined the global, regional, and country-level burdens and trends of liver cancer attributable to HBV–T2DM comorbidity from 1990 to 2019. Additionally, we stratified the disease burden according to the SDI, sex, and age. We reported the age-standardized prevalence and DALY rates of liver cancer attributable to HBV–T2DM comorbidity as our primary results. The results of age-standardized mortality rate are presented in the supplementary material. The total number of prevalent cases, DALYs, and deaths of liver cancer were also reported.

To describe the trends of the age-standardized prevalence, DALY, and mortality rates of liver cancer, we calculated the estimated annual percentage change (EAPC) using Eq. 2. The EAPC is a widely used measure for assessing the ASR trend over a time interval, as proposed by Hankey et al. [25, 30–32]. The value of *β* in Eq. 2 was obtained by fitting the following regression model: ${\text{ln}}(ASR)=\alpha +\beta x+\varepsilon$, where *x* is the calendar year. The 95% CI of the EAPC was calculated by fitting the lower and upper 95% CI boundaries of *β* in Eq. 2.

2 $$EAPC = 100 \times \left( {e^{\beta } - 1} \right)$$

The ASR was considered to increase when the lower 95% CI boundary of the EAPC was greater than 0, and vice versa. If the 95% CI included 0, the ASR was considered stable. We also calculated the relative percentage change in the total cases, DALYs, and deaths of liver cancer from 1990 to 2019. All analyses were performed using the R software package version 4.1.1. A two-tailed *p* value of < 0.05 was considered statistically significant.

## Results

### Global Burden of Liver Cancer Due to HBV-T2DM Comorbidity in 2019

**Fig. 1:**
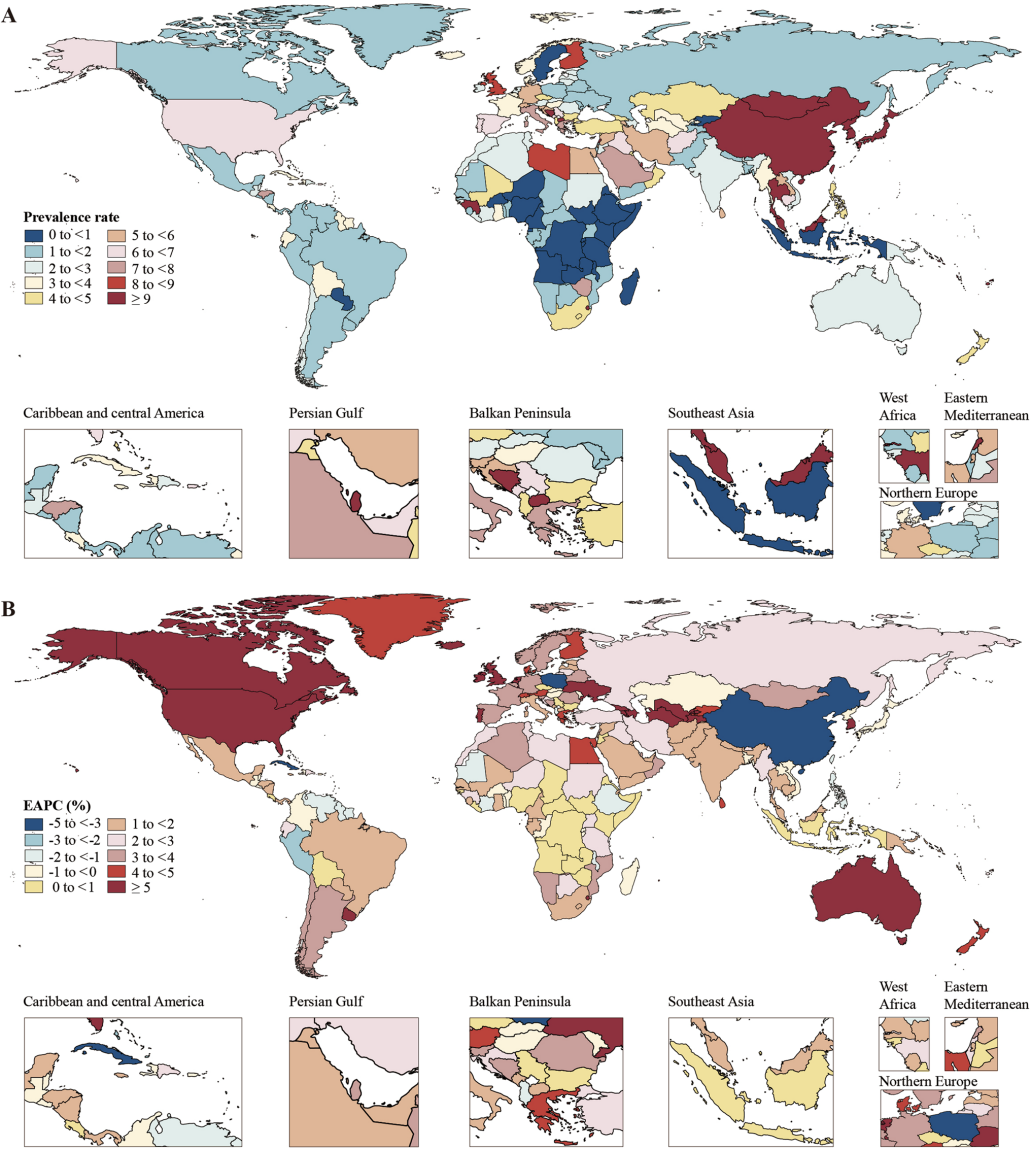
The age-standardized prevalence (**A**) rates (per 10,000,000 individuals) of liver cancer due to HBV–T2DM comorbidity in 204 countries and territories in 2019; EAPCs in the age-standardized prevalence (**B**) rates of liver cancer due to HBV–T2DM comorbidity in 204 countries and territories from 1990 to 2019. EAPC estimated annual percentage change, HBV hepatitis B virus, T2DM type 2 diabetes mellitus

**Fig. 2:**
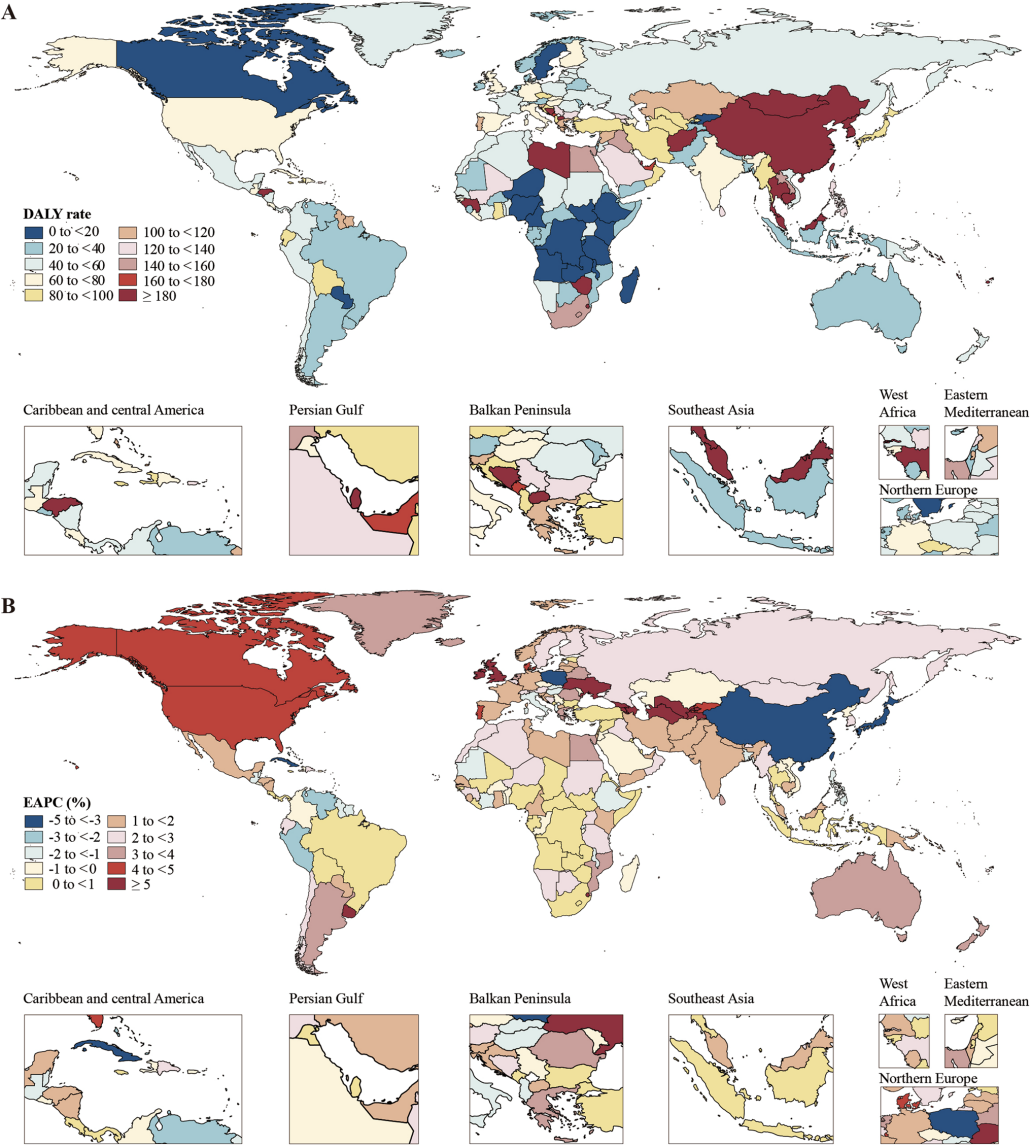
The age-standardized DALY (**A**) rates (per 10,000,000 individuals) of liver cancer due to HBV–T2DM comorbidity in 204 countries and territories in 2019; EAPCs in the age-standardized DALY (**B**) rates of liver cancer due to HBV–T2DM comorbidity in 204 countries and territories from 1990 to 2019. DALY disability-adjusted life year, EAPC estimated annual percentage change, HBV hepatitis B virus, T2DM type 2 diabetes mellitus

In 2019, the global age-standardized prevalence and DALY rates of liver cancer attributable to the comorbidity were 9.9 (95% UI, 8.4 to 11.5) and 182.4 (95% UI, 154.9 to 212.7) per 10,000,000 individuals, respectively (Table 1). Among the 21 GBD regions, the age-standardized prevalence rates were highest in high-income Asia Pacific (34.8; 95% UI, 28.2 to 41.9), East Asia (27.9; 95% UI, 22.5 to 33.8), and Southeast Asia (6.6; 95% UI, 5.0 to 8.7), while the lowest burdens were observed in Eastern Sub-Saharan Africa (0.6; 95% UI, 0.5 to 0.9), Central Sub-Saharan Africa (0.7; 95% UI, 0.5 to 1.0), and Tropical Latin America (1.2; 95% UI, 1.0 to 1.4). Regarding age-standardized DALY rates, East Asia (519.1; 95% UI, 423.7 to 634.7), high-income Asia Pacific (289.8; 95% UI, 247.7 to 334.7), and Oceania (159.3; 95% UI, 123.6 to 205.3) carried the highest burdens, while the lowest burdens were found in Eastern Sub-Saharan Africa (17.7; 95% UI, 12.9 to 23.8), Central Sub-Saharan Africa (20.0; 95% UI, 13.6 to 29.2), and Southern Latin America (29.3; 95% UI, 20.5 to 40.9). The age-standardized prevalence and DALY rates of liver cancer for the 204 countries and territories in 2019 are shown in Figs. 1A, 2A and Table S3. In terms of the total prevalent and DALY cases, an estimated 8289.7 (95% UI, 7040.7 to 9625.1) liver cancer cases and 152,945.2 (95% UI, 129,587.1 to 178,641.4) DALYs were attributable to HBV–T2DM comorbidity in 2019 (Table S4). Among the 21 regions, both the highest number of prevalent cases (5849.1; 95% UI, 4717.0 to 7108.3) and the highest DALYs (108,747.6; 95% UI, 88,445.7 to 133,364.2) were observed in East Asia (Table S4). The prevalent and DALY cases of liver cancer attributable to the comorbidity for 204 countries are shown in Table S4, Figure S2A, and Figure S3A. The patterns of age-standardized mortality rates and the number of death cases in 2019 show similarities to the DALYs figures, as presented in Table S5, Figure S4A, and Figure S5A.

**Table 1:** Age-standardized prevalence and DALY rates (per 10,000,000 individuals) for liver cancer due to HBV–T2DM comorbidity in 1990 and 2019 and the EAPCs from 1990 to 2019, stratified by SDI and region

Location	Age-standardized prevalence rate			Age-standardized DALY rate		
	1990 No. (95% UI)	2019 No. (95% UI)	EAPC % (95% CI)	1990 No. (95% UI)	2019 No. (95% UI)	EAPC % (95% CI)
Global	8.9 (7.4, 10.4)	9.9 (8.4, 11.5)	− 0.87 (− 1.38, − 0.36)	243.5 (208.0, 285.9)	182.4 (154.9, 212.7)	− 2.39 (− 2.96, − 1.82)
SDI						
Low SDI	2.3 (1.8, 2.8)	1.7 (1.3, 2.0)	0.69 (0.58, 0.80)	63.6 (51.6, 77.3)	45.5 (36.2, 56.2)	0.64 (0.52, 0.76)
Low-middle SDI	4.8 (4.1, 5.6)	4.1 (3.5, 4.8)	− 1.09 (− 1.58, − 0.60)	136.4 (116.4, 158.6)	106.9 (91.5, 122.8)	− 1.40 (− 1.89, − 0.90)
Middle SDI	17.0 (14.0, 20.4)	15.2 (12.5, 18.3)	− 1.94 (− 2.65, − 1.22)	476.3 (398.9, 570.2)	301.8 (250.1, 361.3)	− 3.12 (− 3.81, − 2.43)
High-middle SDI	10.1 (8.3, 12.0)	11.4 (9.2, 13.7)	− 2.03 (− 2.67, − 1.38)	277.6 (228.8, 329.9)	197.2 (162.9, 238.7)	− 3.73 (− 4.39, − 3.07)
High SDI	2.9 (2.5, 3.3)	14.2 (12.0, 16.7)	4.27 (3.78, 4.77)	57.2 (50.4, 64.9)	141.0 (121.1, 163.4)	1.55 (1.10, 2.01)
Regions						
High-income Asia Pacific	6.3 (5.5, 7.1)	34.8 (28.2, 41.9)	3.72 (3.12, 4.33)	111.2 (97.3, 126.3)	289.8 (247.7, 334.7)	0.82 (− 0.01, 1.65)
Central Asia	1.3 (0.9, 1.7)	5.0 (3.6, 6.7)	4.72 (4.08, 5.37)	34.3 (25.5, 46.3)	134.8 (97.1, 180.0)	4.70 (4.03, 5.37)
East Asia	31.4 (25.7, 37.9)	27.9 (22.5, 33.8)	− 2.95 (− 3.69, − 2.21)	877.7 (725.4, 1057.8)	519.1 (423.7, 634.7)	− 4.38 (− 5.12, − 3.63)
South Asia	1.8 (1.4, 2.1)	2.3 (1.9, 2.8)	1.32 (1.21, 1.43)	48.6 (40.2, 57.4)	61.7 (51.1, 74.2)	1.17 (1.05, 1.28)
Southeast Asia	4.3 (3.5, 5.2)	6.6 (5.0, 8.7)	1.31 (1.15, 1.47)	119.8 (98.6, 143.0)	158.5 (120.4, 205.7)	0.75 (0.58, 0.92)
Australasia	0.4 (0.3, 0.5)	2.7 (1.9, 3.9)	5.14 (4.55, 5.73)	8.7 (6.6, 11.3)	39.8 (29.6, 53.8)	3.79 (3.45, 4.12)
Caribbean	4.5 (3.4, 5.9)	3.7 (2.6, 5.1)	− 1.03 (− 1.87, − 0.18)	118.4 (90.1, 152.3)	93.3 (65.1, 128.2)	− 1.20 (− 2.08, − 0.32)
Central Europe	2.3 (1.9, 2.9)	3.6 (2.6, 4.8)	− 0.05 (− 0.40, 0.31)	61.4 (49.2, 76.2)	82.5 (61.1, 112.3)	− 0.57 (− 0.93, − 0.20)
Eastern Europe	0.5 (0.4, 0.5)	1.6 (1.3, 2.0)	3.32 (3.00, 3.65)	12.3 (10.5, 14.3)	41.7 (33.7, 51.6)	3.14 (2.76, 3.53)
Western Europe	1.1 (0.8, 1.3)	5.8 (4.4, 7.7)	4.04 (3.76, 4.32)	20.0 (15.6, 25.2)	62.5 (47.5, 81.2)	1.94 (1.82, 2.06)
Andean Latin America	2.9 (2.3, 3.6)	2.6 (1.9, 3.3)	− 0.66 (− 1.03, − 0.28)	76.3 (61.3, 93.7)	62.3 (45.5, 81.1)	− 0.93 (− 1.32, − 0.53)
Central Latin America	1.9 (1.5, 2.5)	2.1 (1.6, 2.9)	0.40 (0.01, 0.79)	51.9 (40.1, 66.9)	51.3 (38.1, 70.0)	0.00 (− 0.41, 0.41)
Southern Latin America	0.4 (0.3, 0.6)	1.3 (0.9, 2.0)	3.84 (3.68, 4.00)	10.8 (7.7, 14.8)	29.3 (20.5, 40.9)	3.32 (3.16, 3.47)
Tropical Latin America	0.9 (0.8, 1.1)	1.2 (1.0, 1.4)	1.15 (0.93, 1.37)	25.2 (21.8, 28.8)	30.4 (25.9, 35.4)	0.85 (0.64, 1.06)
North Africa and Middle East	3.1 (2.5, 3.9)	4.8 (3.6, 6.2)	2.54 (2.31, 2.78)	83.6 (66.3, 103.4)	103.4 (77.1, 135.3)	1.79 (1.61, 1.98)
High-income North America	0.9 (0.8, 1.1)	5.7 (4.4, 7.4)	6.07 (5.59, 6.56)	16.2 (14.0, 18.5)	68.8 (56.2, 84.0)	4.77 (4.35, 5.20)
Oceania	6.1 (4.7, 7.7)	6.0 (4.6, 7.8)	1.33 (1.26, 1.40)	167.6 (131.2, 207.9)	159.3 (123.6, 205.3)	1.21 (1.14, 1.29)
Central Sub-Saharan Africa	1.2 (0.8, 1.6)	0.7 (0.5, 1.0)	0.46 (0.31, 0.61)	34.1 (24.6, 46.3)	20.0 (13.6, 29.2)	0.40 (0.26, 0.55)
Eastern Sub-Saharan Africa	1.0 (0.7, 1.4)	0.6 (0.5, 0.9)	0.64 (0.45, 0.84)	28.2 (21.2, 38.0)	17.7 (12.9, 23.8)	0.60 (0.39, 0.81)
Southern Sub-Saharan Africa	4.4 (2.9, 7.6)	5.4 (4.5, 6.5)	0.99 (0.32, 1.66)	126.1 (84.7, 219.3)	155.3 (130.2, 185.2)	0.99 (0.23, 1.76)
Western Sub-Saharan Africa	2.8 (2.2, 3.4)	1.7 (1.3, 2.1)	0.68 (0.58, 0.78)	75.9 (60.3, 93.5)	44.7 (34.7, 55.8)	0.60 (0.49, 0.70)

CI confidence interval, DALY disability-adjusted life year, EAPC estimated annual percentage change, HBV hepatitis B virus, SDI sociodemographic index, T2DM type 2 diabetes mellitus, UI uncertainty interval

**Fig. 3:**
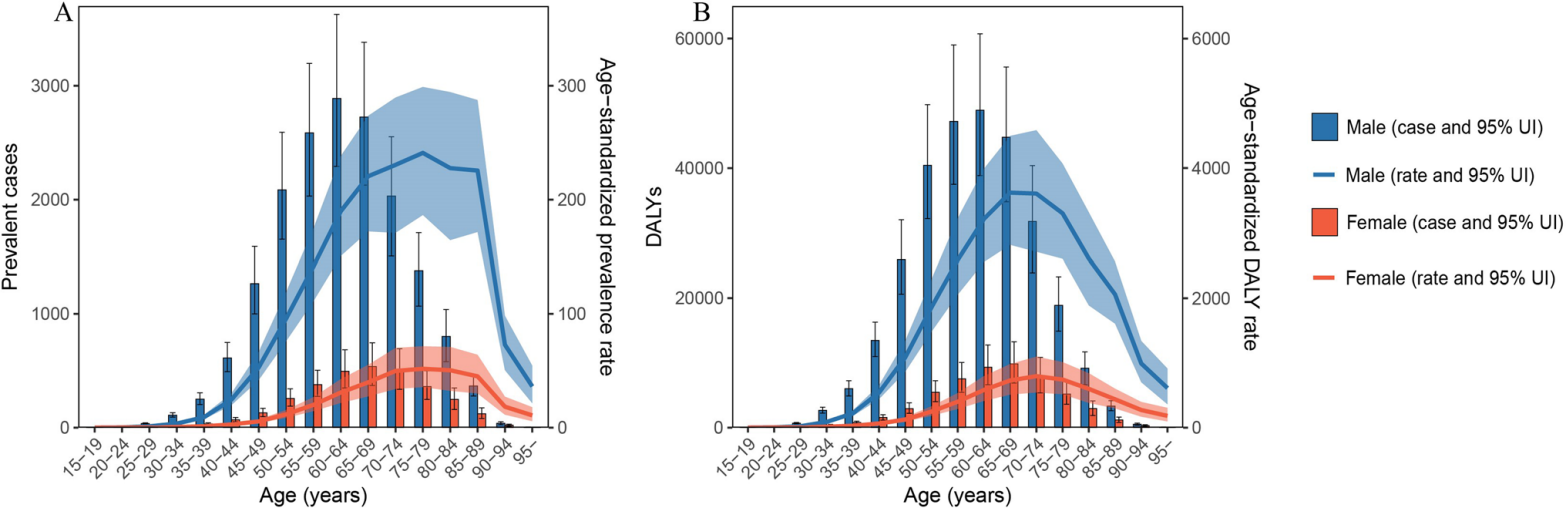
The global numbers and age-standardized rates (per 10,000,000 individuals) for the prevalence (**A**) and DALYs (**B**) of liver cancer due to HBV–T2DM comorbidity in 2019, stratified by age and sex. DALY disability-adjusted life year, HBV hepatitis B virus, T2DM type 2 diabetes mellitus

Upon stratification by the SDI, the middle SDI region had the highest age-standardized prevalence (15.2; 95% UI, 12.5 to 18.3), DALY (301.8; 95% UI, 250.1 to 361.3), and mortality (10.2; 95% UI, 8.5 to 12.3) rates of liver cancer (Table 1 and Table S5). The age-standardized prevalence, DALY, and mortality rates of liver cancer due to the comorbidity, stratified by region for men and women, are presented in Table S6 and Table S7, respectively. Notably, across all regions, men consistently exhibited higher age-standardized prevalence, DALY, and mortality rates of liver cancer than women. The age-standardized prevalence, DALYs, and mortality rates of liver cancer attributable to the comorbidity among adults aged 60 years and older are presented in Table S8. The disease burden of liver cancer attributable to the comorbidity among these older adults is about 10 times higher than that in the general population. The prevalence rate of liver cancer peaked at ages 75–79, the DALY rate peaked at ages 65–69, and the mortality rate peaked at ages 85–89 (Fig. 3 and Figure S6). Upon stratification by sex and age, men had consistently higher prevalence, DALY, and mortality rates of liver cancer across all age groups (Fig. 3 and Figure S6). The middle SDI region had the highest number of prevalent cases, DALYs, and deaths (Table S4). Upon stratification by sex and age group, men consistently had higher numbers of prevalent cases, DALYs, and deaths than women, whereas older adults aged 60–64 years had the highest prevalent cases and DALYs across all age groups (Fig. 3 and Figure S6).

### Trends of Global Burden of Liver Cancer Due to Comorbidity from 1990 to 2019

**Fig. 4:**
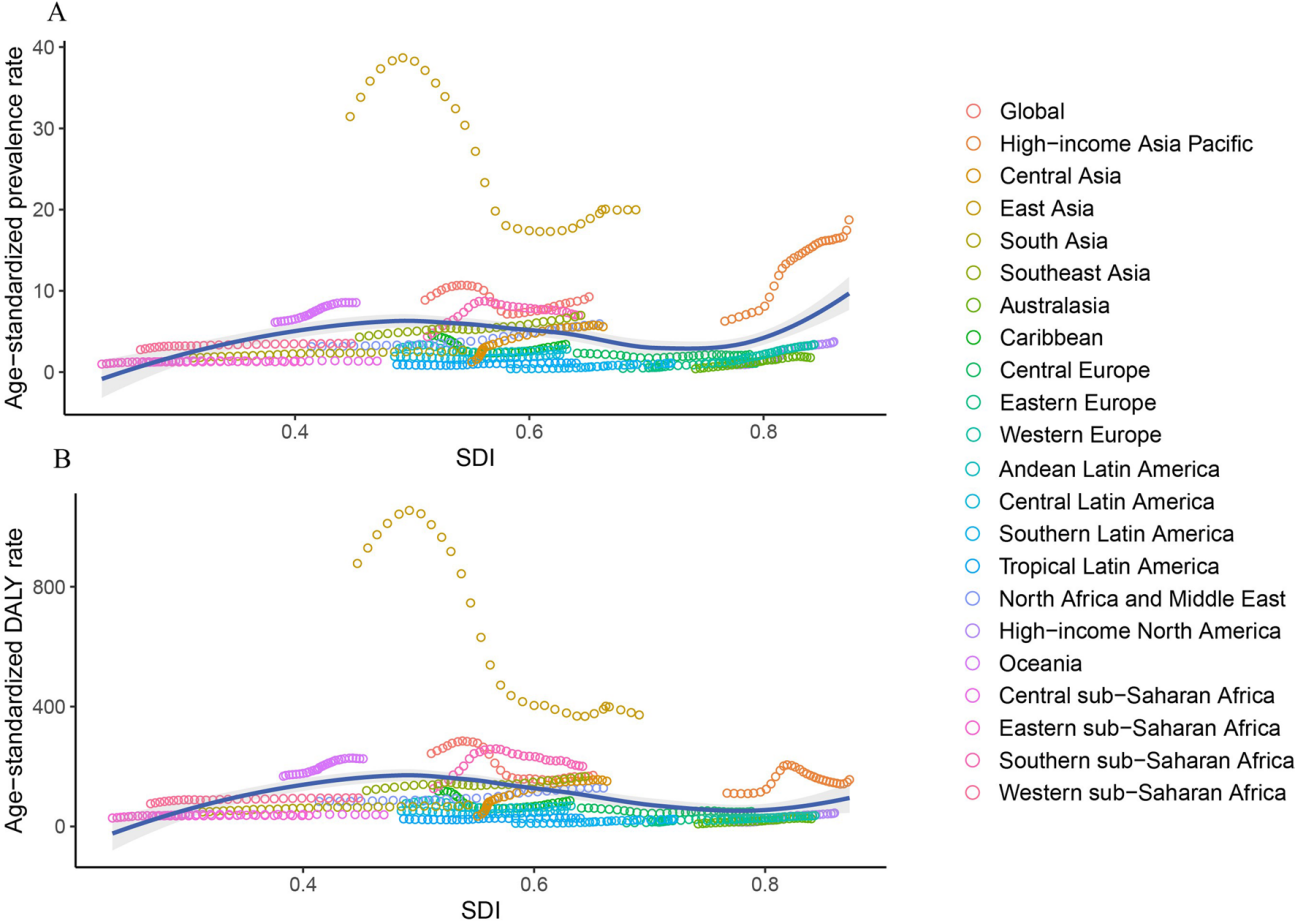
The age-standardized prevalence (**A**) and DALY (**B**) rates (per 10,000,000 individuals) of liver cancer due to HBV–T2DM comorbidity in the 21 GBD regions by SDI during 1990–2019. Each dot represents the disease burden for a year in that region. The blue line, a locally weighted scatterplot smoothing smoother, presents the expected global values based on the SDI values. DALY disability-adjusted life year, GBD global disease of burden, HBV hepatitis B virus, SDI sociodemographic index, T2DM type 2 diabetes mellitus

From 1990 to 2019, 16 out of the 21 GBD regions exhibited an increasing trend of age-standardized prevalence and DALY rates of liver cancer attributable to HBV–T2DM comorbidity (Table 1 and Figure S7). In terms of age-standardized prevalence rates, the regions experiencing the largest annual increases were high-income North America (EAPC = 6.07; 95% UI, 5.59 to 6.56), Australasia (EAPC = 5.14; 95% UI, 4.55 to 5.73), and Central Asia (EAPC = 4.72; 95% UI, 4.08 to 5.37). The regions with the largest annual decreases were East Asia (EAPC =  − 2.95; 95% UI, − 3.69 to − 2.21), the Caribbean (EAPC =  − 1.03; 95% UI, − 1.87 to − 0.18), and Andean Latin America (EAPC =  − 0.66; 95% UI, − 1.03 to − 0.28). For age-standardized DALY rates, the regions with the largest annual increases were high-income North America (EAPC = 4.77; 95% UI, 4.35 to 5.20), Central Asia (EAPC = 4.70; 95% UI, 4.03 to 5.37), and Australasia (EAPC = 3.79; 95% UI, 3.45 to 4.12). The regions experiencing the largest annual decreases in DALY rates were East Asia (EAPC =  − 4.38; 95% UI, − 5.12 to − 3.63), the Caribbean (EAPC =  − 1.20; 95% UI, − 2.08 to − 0.32), and Andean Latin America (EAPC =  − 0.93; 95% UI, − 1.32 to − 0.53). A scatter plot of the SDI value and ASRs stratified by region is shown in Fig. 4. The age-standardized prevalence and DALY rates in most regions showed an increasing trend except for East Asia (Fig. 4). The country-level trends of age-standardized prevalence and DALY rates of liver cancer are shown in Table S3, Figs. 1B, and 2B. Out of 204 countries, 151 (74.02%) exhibited a rising trend in age-standardized prevalence, and 137 (67.16%) showed an increasing trend in age-standardized DALY rates over the last three decades. From 1990 to 2019, the global number of prevalent cases and DALYs of liver cancer attributable to HBV–T2DM comorbidity increased by 171.95% (95% CI 112.66 to 239.31) and 79.98% (95% CI 41.58 to 124.38), respectively (Table S4). Among the 21 regions, Australasia, Central Asia, and high-income North America exhibited the three most substantial relative increases in both prevalent cases and DALYs. The relative changes in prevalent cases and DALYs from 1990 to 2019 in the 204 countries and territories are presented in Table S4, Figure S2B, and Figure S3B. The trends in age-standardized mortality rates and the number of death cases from 1990 to 2019 are similar to those of DALYs, as presented in Table S5, Figure S4B, Figure S5B, and Figure S8.

**Fig. 5:**
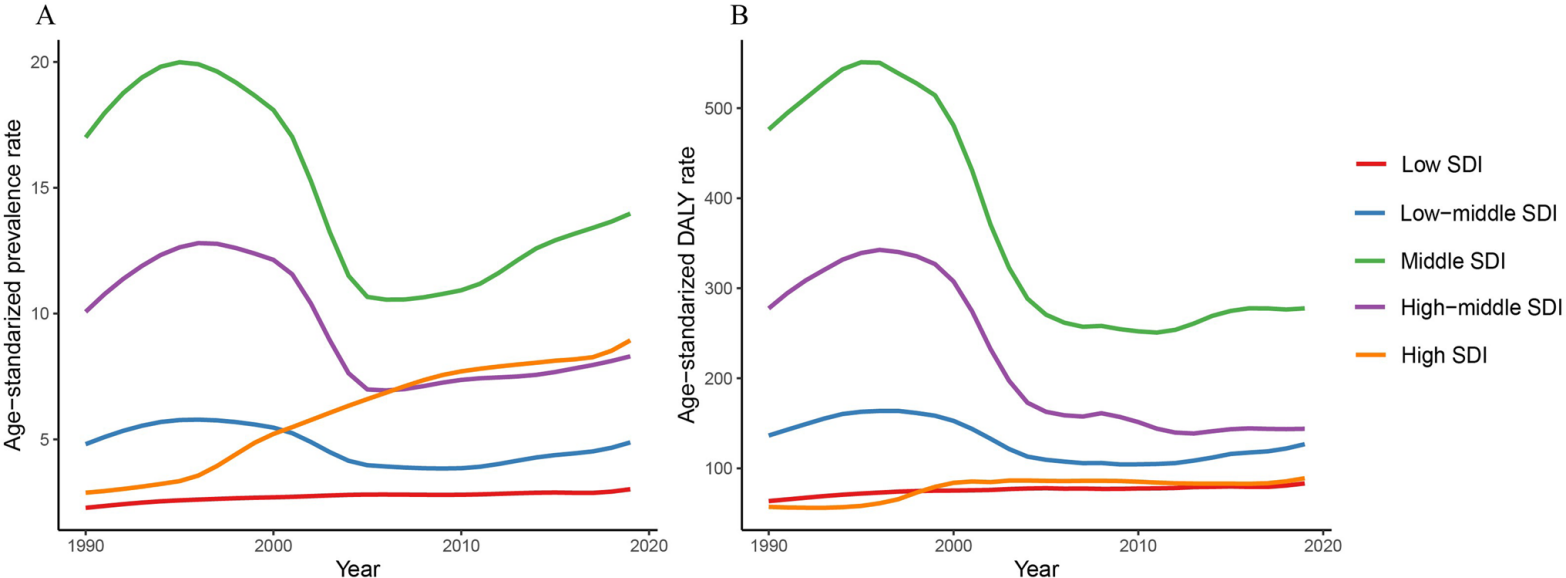
The trends in the age-standardized prevalence (**A**) and DALY (**B**) rates (per 10,000,000 individuals) of liver cancer due to HBV–T2DM comorbidity from 1990 to 2019, stratified by the SDI level. DALY disability-adjusted life year, HBV hepatitis B virus, T2DM type 2 diabetes mellitus

In the stratified results, the high SDI region had the largest annual increases in both the age-standardized prevalence rates (EAPC = 4.27; 95% UI, 3.78 to 4.77), DALY rates (EAPC = 1.55; 95% UI, 1.10 to 2.01), and mortality rates (EAPC = 2.01; 95% UI, 1.57 to 2.45) (Table 1 and Table S5). Figure 5 presents the trends of the ASRs from 1990 to 2019 stratified by the SDI region. A dramatic increase in age-standardized prevalence rates was observed in the high SDI region. In the low–middle, middle, and high-middle SDI regions, the age-standardized prevalence and DALY rates increased from 1990 to 1995, plunged during the next decade, and showed a slight increase after 2005. Similar to the ASR results, the high SDI region had the largest relative increases in terms of the number of prevalent cases (563.94%; 95% CI 425.54 to 717.93) and DALYs (230.03%; 95% CI 165.11 to 303.59) (Table S4). The trends in age-standardized mortality rates and the number of death cases from 1990 to 2019 stratified by the SDI region are similar to those of DALYs, as presented in Table S4, Table S5, and Figure S9.

## Discussion

The disease burden of liver cancer due to HBV–T2DM comorbidity exhibited substantial variation by region. It is noteworthy that the majority of GBD regions (16 out of 21) showed an increasing trend in age-standardized prevalence and DALY rates of liver cancer due to this comorbidity over the past three decades. In regions with high HBV endemicity, the escalating burden of liver cancer due to HBV–T2DM comorbidity could potentially become a considerable public health challenge if the growing trend continues. In regions with low HBV endemicity, comorbid T2DM could become an important factor affecting the prognosis of individuals with HBV infection. Additionally, men and the elderly are critical population groups requiring interventions to alleviate the burden of liver cancer due to HBV–T2DM comorbidity.

High-income North America had the largest annual increases in the age-standardized prevalence and DALY rates of liver cancer due to HBV–T2DM comorbidity. This could be explained by the increase in the prevalence of T2DM and the aging of the HBV-infected population in North America [9, 33]. The prevalence of T2DM in the American population increased from 7% in 2001 to 10% in 2020, perhaps due to the rise in unhealthy lifestyles, poor dietary habits, obesity rates, and the extended life expectancy of people living with T2DM [34]. Although the prevalence of HBV is relatively low in North America, the existing HBV-infected population is rapidly aging [8, 9]. The aging of the HBV-infected American population increases the risk of developing this comorbidity, as T2DM is one of the most common age-associated NCDs [9, 35]. Therefore, comorbid T2DM could potentially impose a significant disease burden on the existing HBV-infected population in North America. This finding highlights the importance of implementing T2DM prevention strategies among individuals with HBV infection in North America. Moreover, healthcare professionals should develop customized treatment and management approaches for those experiencing the dual challenge of HBV–T2DM comorbidity to mitigate their disease progression.

East Asia had the most remarkable decreases in the age-standardized prevalence and DALY rates of liver cancer due to HBV–T2DM comorbidity; however, it remained the region with the highest DALY rate in 2019. In 1990, East Asia reported almost half (45%) of all global HBV-related deaths [36]. Most of the HBV carriers in East Asia were infected through perinatal transmission or during early childhood [37]. Over the past three decades, most countries in East Asia have made remarkable progress in HBV control [36, 37]. For example, the prevalence of HBV among children aged 5 years has decreased to less than 0.5% in China [38]. This progression is primarily attributed to the implementation of timely birth-dose vaccines and increased HBV vaccination coverage among children [36, 37, 39]. Therefore, the disease burden of HBV-related liver cancer showed a decreasing trend during this period. However, a slight increase in the age-standardized prevalence of liver cancer due to comorbid T2DM was observed among HBV-infected individuals after 2005. This finding is consistent with the rising trends in the prevalence of T2DM in East Asia over the past decade [33]. This result indicates that comorbid T2DM could potentially increase the burden of liver cancer in the HBV-infected population in East Asia. Given the large number of HBV-infected adults in this region, healthcare professionals should develop a two-pronged approach that simultaneously addresses HBV control and T2DM management. Tailored strategies are imperative to fulfil the growing needs of individuals affected by both HBV infection and T2DM, with the aim of mitigating the disease burden of liver cancer in East Asia.

In addition, the burden of liver cancer due to the comorbidity also varied across SDIs, with the middle SDI region showing the largest disease burden. Previous studies have shown that the middle SDI region still has a relatively high prevalence of HBV infection [36]. Liu et al. reported a rapid increase in the burden of T2DM in the middle SDI region from 1990 to 2019 [24]. Given the synergistic interaction between HBV infection and T2DM, HBV–T2DM comorbidity could become a major public health concern in the middle SDI region. The high SDI region showed a consistent increasing trend in the disease burden of liver cancer due to the comorbidity. This aligns with findings observed in the high-income North American region. In contrast, regions with low-middle, middle, and high-middle SDI levels experienced a plunge in disease burden from 2000 to 2005, followed by a slow increase afterwards. The reduction in the burden of liver cancer due to HBV–T2DM comorbidity was likely caused by the decreased prevalence of HBV in these regions [36]. However, the slight increase in this burden after 2005 warrants attention from healthcare professionals, as this could be attributable to the rising vulnerability of the HBV-infected population to NCDs due to aging, as well as T2DM-related changes in environmental factors and lifestyles in these countries [24]. These results indicate that, in addition to prevention strategies targeting HBV control, liver cancer in the middle SDI region should be prevented by advocating early lifestyle changes to reduce the risk of T2DM among HBV-infected individuals.

In addition to regional differences, the disease burden of liver cancer due to HBV–T2DM comorbidity was high in men as well as in middle-aged and older adults. Our findings are consistent with previous epidemiological studies. A cohort study conducted in Taiwan involving 2,099 patients with HBV–T2DM comorbidity found that the hazard ratio of developing HCC between men and women was 2.60 (95% CI 1.59 to 4.56) [40]. This finding is also supported by another study using a health insurance database showing a more than twofold risk of developing HCC in men than in women among patients with HBV–T2DM comorbidity (hazard ratio = 2.25, 95% CI 1.89 to 2.68) [41]. A previous study suggested that men were at a higher risk of developing liver cancer after HBV infection due to the immune-suppressive effect of male hormones such as androgen [42]. Moreover, evidence has shown that the prevalence of T2DM is higher in middle-aged men than in women, perhaps because men are more insulin-resistant after puberty and women have a higher obesity threshold for developing T2DM [43]. Thus, sex-specific interventions targeting T2DM development need to be implemented for HBV-infected men. We found that the burden of liver cancer attributable to comorbid T2DM is higher in older adults aged 60 and above compared to the general population. The underlying rationales may be ascribed to the increased risk of T2DM in older adults due to age-related insulin resistance and impaired pancreatic islet function [44]. Given the rapid aging of the population with HBV infection, our findings emphasize the importance for regular screening and effective management of T2DM among older adults living with HBV.

Given the increased disease burden of liver cancer attributable to comorbid T2DM among people living with HBV, it is of utmost importance to implement primary, secondary and tertiary prevention strategies against HBV–T2DM comorbidity. For primary prevention, HBV vaccination should be prioritized for those living with T2DM who have not yet been vaccinated. For example, the U.S. Centers for Disease Control and Prevention has recommended hepatitis B vaccination for unvaccinated people with T2DM [15]. Moreover, the blood glucose meters and injection equipment used by patients with T2DM should be disinfected regularly. Meanwhile, effective T2DM prevention strategies targeting lifestyle changes, including regular exercise, a healthy diet, and smoking cessation, should be advocated and implemented among the HBV-infected population [45]. For secondary prevention, regular screening of T2DM should be readily available for individuals with HBV infection to ensure early detection. For tertiary prevention, customized interventions such as self-care skill development workshops and support groups should be designed and provided to those with the comorbidity to reduce the risk of developing liver cancer.

Our study has some limitations. First, although we obtained pooled parameters for estimating PAFs from large meta-analyses, a more precise estimation could be achieved by using region-, sex-, and age-specific parameters. Future researchers are encouraged to perform such an estimation when more specific subgroup parameters are available. Second, the accuracy of our estimates depends on the quality and quantity of the data from the GBD 2019 database, which is subject to the potential underreporting and misdiagnosis of liver cancer, especially in low-income regions. Third, we only conducted a descriptive analysis of the disease burden, and the effectiveness of strategies for preventing the comorbidity was not examined. Modelling studies are thus warranted in the future to evaluate the effectiveness of different strategies or interventions targeting HBV–T2DM comorbidity. Fourth, we were unable to control for individual confounding factors due to the lack of individual-level data in the GBD 2019 database. Future cohort studies could explore the burden of liver cancer attributable to the comorbidity and risk factors at the individual level.

## Conclusion

Our study estimated the global burden and trends of liver cancer attributable to comorbid T2DM among people living with HBV. The burden of liver cancer attributable to comorbid T2DM varies significantly across regions. Most regions have exhibited a pronounced increasing trend over the past three decades, particularly in high SDI regions. With the growing burden of T2DM and the rapidly aging population with HBV, our findings reveal the hidden threat of HBV–T2DM comorbidity and emphasize the need for two-pronged interventions targeting both HBV infection and T2DM management.

### Supplementary Information

Below is the link to the electronic supplementary material.

Supplementary file1 (PDF 24578 KB)

## Data Availability

The datasets used in this study are publicly available (https://ghdx.healthdata.org/gbd-2019).
