# Supplementary material for: Global Burden and Trends of Primary Liver Cancer Attributable to Comorbid Type 2 Diabetes Mellitus Among People Living with Hepatitis B: An Observational Trend Study from 1990 to 2019
Source: J Epidemiol Glob Health. 2024 May 7;14(2):398–410. doi: 10.1007/s44197-024-00237-1 (PMC11176116; doi:10.1007/s44197-024-00237-1)
Supplement: Supplementary file 1 — Supplementary file1 (PDF 24578 KB) [file 44197_2024_237_MOESM1_ESM.pdf]

Journal of Epidemiology and Global Health

Supplementary material

Global burden and trends of primary liver cancer attributable to comorbid type 2 diabetes mellitus among people living with hepatitis B: an observational trend study from 1990 to 2019

Jinzhao Xie, Xiao Lin, Xiaoyan Fan, Xu Wang, Deng Pan, Jinghua Li, Yuantao Hao, Yusheng Jie, Lei Zhang, Jing Gu*

* Corresponding author:
Jing Gu
Professor
School of Public Health & Global Health Institute
Sun Yat-sen University
Email: gujing5@mail.sysu.edu.cn

Contents

Supplementary Methods 4

Table S1. Countries included in various SDI categories 6

Table S2. PAF of HBV–T2DM comorbidity on liver cancer in 1990 and 2019 7

Figure S1. PAF of HBV–T2DM comorbidity on liver cancer in 2019 stratified by country (A), SDI (B), and age and sex (C). HBV hepatitis B virus, PAF population attributable fraction, SDI socio-demographic index, T2DM type 2 diabetes mellitus 13

Table S3. Age-standardized prevalence, DALY, and mortality rates (per 10,000,000 individuals) of liver cancer due to HBV–T2DM comorbidity in 1990 and 2019 and EAPCs from 1990 to 2019, stratified by country 14

Table S4. Prevalent cases, DALYs, and deaths of liver cancer due to HBV–T2DM comorbidity in 1990 and 2019 and relative changes between 1990 and 2019, stratified by SDI, region, and country 33

Figure S2. Prevalent cases (A) of liver cancer due to HBV–T2DM comorbidity in the 204 countries and territories in 2019. Relative changes in the number of prevalent cases (B) of liver cancer due to HBV–T2DM comorbidity in the 204 countries and territories between 1990 and 2019. HBV hepatitis B virus, T2DM type 2 diabetes mellitus 57

Figure S3. DALYs (A) of liver cancer due to HBV–T2DM comorbidity in the 204 countries and territories in 2019. Relative changes in DALYs (B) of liver cancer due to HBV–T2DM comorbidity in the 204 countries and territories between 1990 and 2019. DALY disability-adjusted life year, HBV hepatitis B virus, T2DM type 2 diabetes mellitus 58

Table S5. Age-standardized mortality rates (per 10,000,000 individuals) for liver cancer due to HBV–T2DM comorbidity in 1990 and 2019 and the EAPCs from 1990 to 2019, stratified by SDI and region 59

Figure S4. The age-standardized mortality (A) rates (per 10,000,000 individuals) of liver cancer due to HBV–T2DM comorbidity in 204 countries and territories in 2019; EAPCs in the age-standardized mortality (B) rates of liver cancer due to HBV–T2DM comorbidity in 204 countries and territories from 1990 to 2019. EAPC estimated annual percentage change, HBV hepatitis B virus, T2DM type 2 diabetes mellitus 60

Figure S5. Deaths (A) of liver cancer due to HBV–T2DM comorbidity in the 204 countries and territories in 2019. Relative changes in deaths (B) of liver cancer due to HBV–T2DM comorbidity in the 204 countries and territories between 1990 and 2019. HBV hepatitis B virus, T2DM type 2 diabetes

mellitus 61

Table S6. Age-standardized prevalence, DALY, and mortality rates (per 10,000,000 individuals) for liver cancer due to HBV–T2DM comorbidity in 1990 and 2019 among men and the EAPCs from 1990 to 2019, stratified by SDI and region 62

Table S7. Age-standardized prevalence, DALY, and mortality rates (per 10,000,000 individuals) for liver cancer due to HBV–T2DM comorbidity in 1990 and 2019 among women and the EAPCs from 1990 to 2019, stratified by SDI and region 65

Table S8. Age-standardized prevalence, DALY, and mortality rates (per 10,000,000 individuals) for liver cancer due to HBV–T2DM comorbidity in 1990 and 2019 among adults aged 60 years and over and the EAPCs from 1990 to 2019, stratified by SDI and region.. 68

Figure S6. The global numbers and age-standardized rates (per 10,000,000 individuals) for the deaths of liver cancer due to HBV–T2DM comorbidity in 2019, stratified by age and sex. HBV hepatitis B virus, T2DM type 2 diabetes mellitus 72

Figure S7. EAPCs of the age-standardized prevalence (A), DALY (B), and mortality (C) rates (per 10,000,000 individuals) of liver cancer due to HBV–T2DM comorbidity from 1990 to 2019 for the 21 GBD regions. ASR age-standardized rate, DALY disability-adjusted life year, EAPC estimated annual percentage change, GBD global disease of burden, HBV hepatitis B virus, T2DM type 2 diabetes mellitus 73

Figure. S8. The age-standardized mortality rates (per 10,000,000 individuals) of liver cancer due to HBV–T2DM comorbidity in the 21 GBD regions by SDI during 1990–2019. Each dot represents the disease burden for a year in that region. The blue line, a locally weighted scatterplot smoothing smoother, presents the expected global values based on the SDI values. GBD global disease of burden, HBV hepatitis B virus, SDI sociodemographic index, T2DM type 2 diabetes mellitus... 74

Figure S9. The trends in the age-standardized mortality rates (per 10,000,000 individuals) of liver cancer due to HBV–T2DM comorbidity from 1990 to 2019, stratified by the SDI level. HBV hepatitis B virus, T2DM type 2 diabetes mellitus... 75

Reference 76

Supplementary Methods

1. The definitions for hepatitis B virus (HBV) infection, type 2 diabetes mellitus (T2DM), and primary liver cancer in this study.

1.1 HBV infection

HBV infection is defined as an infection of the liver caused by the hepatitis B virus, characterized by a positive test for hepatitis B surface antigen (HBsAg) in the blood [1, 2].

1.2 T2DM

In the Global Burden of Disease (GBD) 2019 study, T2DM is defined as a condition characterized by a fasting plasma glucose level of at least 7 mmol/L (126 mg/dL) or individuals who are currently treated with drugs or insulin to manage their blood glucose levels [3].

1.3 Primary liver cancer

In the GBD 2019 study, primary liver cancer is defined as malignant neoplasms originating in the liver's parenchymal cells. This category encompasses various histological types, including hepatocellular carcinoma (HCC), which is the most prevalent, along with other specified and unspecified liver cancer types. The corresponding International Classification of Diseases 10th revision (ICD-10) codes for primary liver cancer in the GBD 2019 are C22.0–C22.8 [4].

2. The search strategy for the GBD 2019 database

Search strategy for the GBD 2019 database

Variable	Search strategy
GBD Estimate	Cause of death or injury
Measure	Prevalence; Disability-Adjusted Life Years (DALYs); Deaths
Metric	Number; Rate
Cause	Liver cancer due to hepatitis B; Diabetes mellitus type 2
Location	Global; All GBD regions; All countries and territories; Low SDI; Low-middle SDI; Middle SDI; High-middle SDI; High SDI
Ages	All ages; Age-standardized; 15-19 years; 20-24 years; 25-29 years; 30-34 years; 35-39 years; 40-44 years; 45-49 years; 50-54 years; 55-59 years; 60-64 years; 65-69 years; 70-74 years; 75-79 years; 80-84 years; 85-89 years; 90-94 years; 95+ years
Sex	Both; Male; Female
Year	1990; 1991; 1992; 1993; 1994; 1995; 1996; 1997; 1998; 1999; 2000; 2001; 2002; 2003; 2004; 2005; 2006; 2007; 2008; 2009; 2010; 2011; 2012; 2013; 2014; 2015; 2016; 2017; 2018; 2019

GBD global burden of disease, SDI sociodemographic index.

3. The search strategy to estimate the relative risk (RR) in Equation 1.

We conducted a systematic search of Medline and Web of Science using the terms "(liver cancer OR liver neoplasms OR hepatocellular carcinoma OR HCC) AND (hepatitis B virus OR HBV) AND (diabetes mellitus OR T2DM) AND (meta-analysis)" Our search was confined to studies published from 1 January 2013 to 24 August 2023, with a focus on meta-analyses and those written in English.

From the initial search, we identified a total of 19 articles. We excluded 16 articles that were not related to the RR in Equation 1. Our review revealed two articles that provided the estimates for the RR in Equation 1 [5, 6]. Ultimately, we selected the article that covered a broader range of studies to estimate the RR in Equation 1 [5].

Table S1. Countries included in various SDI categories

SDI category	SDI range	Country
Low SDI	0 to < 0.455	Afghanistan, Benin, Burkina Faso, Burundi, Central African Republic, Chad, Cote d'Ivoire, Democratic Republic of the Congo, Eritrea, Ethiopia, Gambia, Guinea, Guinea-Bissau, Haiti, Liberia, Madagascar, Malawi, Mali, Mozambique, Nepal, Niger, Pakistan, Papua New Guinea, Rwanda, Senegal, Sierra Leone, Solomon Islands, Somalia, South Sudan, Syrian Arab Republic, Togo, Uganda, United Republic of Tanzania, Yemen
Low-middle SDI	0.455 to < 0.608	Angola, Bangladesh, Belize, Bhutan, Bolivia (Plurinational State of), Cabo Verde, Cambodia, Cameroon, Comoros, Congo, Democratic People's Republic of Korea, Djibouti, Dominican Republic, El Salvador, Eswatini, Ghana, Guatemala, Honduras, India, Kenya, Kiribati, Kyrgyzstan, Lao People's Democratic Republic, Lesotho, Maldives, Marshall Islands, Mauritania, Micronesia (Federated States of), Mongolia, Morocco, Myanmar, Nicaragua, Nigeria, Palestine, Sao Tome and Principe, Sudan, Tajikistan, Timor-Leste, Tuvalu, Vanuatu, Venezuela (Bolivarian Republic of), Zambia, Zimbabwe
Middle SDI	0.608 to < 0.690	Albania, Algeria, Armenia, Azerbaijan, Botswana, Brazil, China, Colombia, Costa Rica, Cuba, Ecuador, Egypt, Equatorial Guinea, Fiji, Gabon, Grenada, Guyana, Indonesia, Iran (Islamic Republic of), Iraq, Jamaica, Mexico, Namibia, Nauru, Panama, Paraguay, Peru, Philippines, Saint Lucia, Saint Vincent and the Grenadines, Samoa, South Africa, Suriname, Thailand, Tokelau, Tonga, Tunisia, Turkmenistan, Uzbekistan, Viet Nam
High-middle SDI	0.690 to < 0.805	American Samoa, Antigua and Barbuda, Argentina, Bahamas, Bahrain, Barbados, Belarus, Bosnia and Herzegovina, Bulgaria, Chile, Cook Islands, Croatia, Dominica, Georgia, Greece, Greenland, Hungary, Israel, Italy, Jordan, Kazakhstan, Lebanon, Libya, Malaysia, Malta, Mauritius, Montenegro, Niue, North Macedonia, Northern Mariana Islands, Oman, Palau, Poland, Portugal, Republic of Moldova, Romania, Russian Federation, Saint Kitts and Nevis, Saudi Arabia, Serbia, Seychelles, Spain, Sri Lanka, Trinidad and Tobago, Turkey, Ukraine, United States Virgin Islands, Uruguay
High SDI	0.805 to 1	Andorra, Australia, Austria, Belgium, Bermuda, Brunei Darussalam, Canada, Cyprus, Czechia, Denmark, Estonia, Finland, France, Germany, Guam, Iceland, Ireland, Japan, Kuwait, Latvia, Lithuania, Luxembourg, Monaco, Netherlands, New Zealand, Norway, Puerto Rico, Qatar, Republic of Korea, San Marino, Singapore, Slovakia, Slovenia, Sweden, Switzerland, Taiwan (Province of China), United Arab Emirates, United Kingdom, United States of America

SDI sociodemographic index.

Table S2. PAF of HBV–T2DM comorbidity on liver cancer in 1990 and 2019

Location	PAF 1990<br>% (95% UI)	PAF 2019<br>% (95% UI)
**Global**	1.31 (1.20, 1.43)	2.64 (2.43, 2.87)
**SDI**		
Low SDI	0.74 (0.67, 0.83)	1.20 (1.08, 1.34)
Low-middle SDI	1.01 (0.91, 1.11)	2.26 (2.05, 2.49)
Middle SDI	1.19 (1.08, 1.31)	2.71 (2.49, 2.94)
High-middle SDI	1.52 (1.39, 1.66)	3.05 (2.79, 3.32)
High SDI	2.04 (1.87, 2.20)	4.11 (3.79, 4.42)
**Region**		
High-income Asia Pacific	1.65 (1.50, 1.80)	3.27 (2.98, 3.58)
Central Asia	0.95 (0.87, 1.05)	2.22 (2.04, 2.41)
East Asia	1.38 (1.24, 1.53)	2.94 (2.70, 3.21)
South Asia	1.09 (0.98, 1.22)	2.54 (2.29, 2.81)
Southeast Asia	0.88 (0.81, 0.97)	2.17 (2.00, 2.36)
Australasia	1.04 (0.94, 1.15)	2.44 (2.19, 2.71)
Caribbean	1.91 (1.78, 2.06)	3.76 (3.49, 4.05)
Central Europe	2.03 (1.86, 2.20)	4.33 (3.95, 4.68)
Eastern Europe	1.23 (1.11, 1.36)	2.04 (1.86, 2.25)
Western Europe	2.21 (2.02, 2.40)	4.28 (3.90, 4.68)
Andean Latin America	0.69 (0.63, 0.75)	1.81 (1.66, 1.96)
Central Latin America	1.73 (1.60, 1.87)	3.84 (3.55, 4.11)
Southern Latin America	1.24 (1.14, 1.34)	2.84 (2.57, 3.09)
Tropical Latin America	1.35 (1.23, 1.46)	2.57 (2.34, 2.80)
North Africa and Middle East	0.96 (0.87, 1.05)	2.52 (2.30, 2.77)
High-income North America	2.39 (2.18, 2.59)	4.78 (4.45, 5.13)
Oceania	1.85 (1.68, 2.03)	3.55 (3.26, 3.89)
Central Sub-Saharan Africa	0.79 (0.71, 0.88)	1.18 (1.06, 1.31)
Eastern Sub-Saharan Africa	0.51 (0.46, 0.57)	0.70 (0.63, 0.78)
Southern Sub-Saharan Africa	0.94 (0.85, 1.04)	2.02 (1.85, 2.20)
Western Sub-Saharan Africa	0.50 (0.45, 0.56)	0.73 (0.65, 0.81)
**Country**		
Afghanistan	1.37 (1.23, 1.52)	1.71 (1.51, 1.93)
Albania	0.64 (0.57, 0.72)	2.16 (1.93, 2.41)
Algeria	1.02 (0.92, 1.14)	3.11 (2.79, 3.48)
American Samoa	3.25 (2.97, 3.60)	8.11 (7.46, 8.85)
Andorra	1.15 (1.01, 1.30)	3.05 (2.70, 3.42)
Angola	0.80 (0.72, 0.90)	1.10 (0.98, 1.22)
Antigua and Barbuda	2.27 (2.09, 2.47)	4.55 (4.14, 4.98)
Argentina	1.31 (1.21, 1.42)	2.63 (2.37, 2.88)
Armenia	1.22 (1.12, 1.34)	3.30 (3.02, 3.62)
Australia	1.07 (0.96, 1.18)	2.48 (2.22, 2.77)
Austria	1.40 (1.26, 1.54)	3.16 (2.80, 3.49)

Location	PAF 1990<br>% (95% UI)	PAF 2019<br>% (95% UI)
Azerbaijan	0.97 (0.87, 1.06)	2.56 (2.29, 2.86)
Bahamas	1.68 (1.52, 1.85)	3.94 (3.59, 4.32)
Bahrain	1.54 (1.38, 1.70)	5.84 (5.33, 6.43)
Bangladesh	0.77 (0.70, 0.85)	1.90 (1.75, 2.08)
Barbados	2.78 (2.58, 2.97)	5.33 (4.91, 5.77)
Belarus	1.30 (1.17, 1.43)	1.91 (1.69, 2.12)
Belgium	1.77 (1.57, 1.96)	3.32 (2.97, 3.67)
Belize	0.96 (0.88, 1.04)	2.31 (2.10, 2.53)
Benin	0.51 (0.46, 0.57)	0.87 (0.78, 0.97)
Bermuda	1.76 (1.60, 1.93)	4.03 (3.64, 4.43)
Bhutan	0.72 (0.64, 0.81)	2.00 (1.82, 2.20)
Bolivia (Plurinational State of)	0.81 (0.73, 0.90)	1.75 (1.58, 1.93)
Bosnia and Herzegovina	1.56 (1.40, 1.73)	5.48 (4.97, 6.03)
Botswana	0.77 (0.68, 0.85)	1.77 (1.59, 1.96)
Brazil	1.36 (1.24, 1.48)	2.58 (2.35, 2.82)
Brunei Darussalam	2.25 (2.09, 2.45)	4.77 (4.41, 5.18)
Bulgaria	2.20 (2.00, 2.41)	4.16 (3.77, 4.58)
Burkina Faso	0.59 (0.53, 0.66)	0.84 (0.76, 0.93)
Burundi	0.57 (0.51, 0.64)	0.69 (0.62, 0.78)
Cabo Verde	0.80 (0.72, 0.89)	1.92 (1.73, 2.13)
Cambodia	0.54 (0.49, 0.60)	1.99 (1.80, 2.19)
Cameroon	0.47 (0.42, 0.53)	0.85 (0.76, 0.94)
Canada	0.86 (0.77, 0.93)	2.59 (2.27, 2.90)
Central African Republic	0.89 (0.80, 1.00)	1.35 (1.21, 1.50)
Chad	0.56 (0.49, 0.62)	0.72 (0.64, 0.79)
Chile	1.22 (1.10, 1.35)	3.47 (3.14, 3.84)
China	1.38 (1.24, 1.54)	2.94 (2.70, 3.21)
Colombia	1.53 (1.38, 1.69)	3.44 (3.14, 3.75)
Comoros	0.53 (0.47, 0.58)	0.98 (0.89, 1.09)
Congo	0.94 (0.85, 1.05)	1.56 (1.40, 1.74)
Cook Islands	2.37 (2.16, 2.60)	7.19 (6.64, 7.79)
Costa Rica	1.43 (1.30, 1.57)	3.69 (3.32, 4.05)
Croatia	2.27 (2.07, 2.48)	4.67 (4.21, 5.17)
Cuba	2.44 (2.24, 2.67)	4.91 (4.48, 5.39)
Cyprus	2.52 (2.28, 2.77)	3.91 (3.55, 4.29)
Czechia	2.86 (2.59, 3.15)	6.78 (6.18, 7.37)
Cote d'Ivoire	0.45 (0.40, 0.51)	0.93 (0.84, 1.02)
Democratic People's Republic of Korea	1.11 (1.00, 1.25)	2.46 (2.21, 2.71)
Democratic Republic of the Congo	0.76 (0.68, 0.85)	1.17 (1.04, 1.30)
Denmark	1.26 (1.12, 1.39)	2.87 (2.60, 3.14)
Djibouti	0.42 (0.37, 0.47)	0.97 (0.87, 1.08)
Dominica	2.47 (2.27, 2.70)	5.76 (5.28, 6.29)

Location	PAF 1990<br>% (95% UI)	PAF 2019<br>% (95% UI)
Dominican Republic	0.68 (0.62, 0.74)	1.87 (1.71, 2.04)
Ecuador	0.83 (0.76, 0.91)	2.26 (2.06, 2.45)
Egypt	0.66 (0.60, 0.73)	1.94 (1.74, 2.16)
El Salvador	1.10 (1.01, 1.20)	3.27 (2.99, 3.54)
Equatorial Guinea	0.89 (0.80, 0.99)	1.13 (1.01, 1.26)
Eritrea	0.49 (0.44, 0.56)	0.84 (0.74, 0.94)
Estonia	1.47 (1.32, 1.62)	2.87 (2.60, 3.16)
Eswatini	0.70 (0.62, 0.78)	1.57 (1.42, 1.72)
Ethiopia	0.59 (0.52, 0.66)	0.55 (0.48, 0.61)
Fiji	2.50 (2.28, 2.76)	6.55 (6.09, 7.06)
Finland	2.25 (2.07, 2.44)	4.77 (4.31, 5.22)
France	1.00 (0.91, 1.09)	2.05 (1.83, 2.27)
Gabon	1.15 (1.03, 1.27)	1.93 (1.74, 2.15)
Gambia	0.40 (0.35, 0.45)	0.83 (0.75, 0.92)
Georgia	1.53 (1.38, 1.69)	4.32 (3.95, 4.70)
Germany	3.04 (2.74, 3.39)	5.61 (5.06, 6.10)
Ghana	0.57 (0.51, 0.64)	1.22 (1.10, 1.35)
Greece	1.71 (1.54, 1.88)	3.81 (3.39, 4.20)
Greenland	0.45 (0.39, 0.53)	2.21 (1.96, 2.47)
Grenada	2.24 (2.07, 2.43)	4.79 (4.40, 5.21)
Guam	1.74 (1.58, 1.95)	4.01 (3.66, 4.42)
Guatemala	1.02 (0.93, 1.12)	2.83 (2.60, 3.04)
Guinea	0.53 (0.47, 0.59)	0.81 (0.73, 0.89)
Guinea-Bissau	0.55 (0.49, 0.61)	0.89 (0.80, 0.99)
Guyana	1.88 (1.71, 2.05)	4.41 (4.06, 4.77)
Haiti	1.61 (1.45, 1.78)	2.74 (2.47, 3.02)
Honduras	1.26 (1.13, 1.39)	2.68 (2.42, 2.97)
Hungary	2.38 (2.14, 2.64)	4.64 (4.20, 5.07)
Iceland	1.11 (0.98, 1.25)	2.88 (2.58, 3.19)
India	1.15 (1.02, 1.29)	2.74 (2.47, 3.04)
Indonesia	0.75 (0.68, 0.83)	1.83 (1.65, 2.01)
Iran (Islamic Republic of)	0.79 (0.70, 0.89)	2.78 (2.50, 3.07)
Iraq	1.34 (1.21, 1.48)	2.58 (2.36, 2.81)
Ireland	0.75 (0.67, 0.84)	2.71 (2.42, 3.03)
Israel	1.30 (1.17, 1.43)	2.58 (2.34, 2.82)
Italy	2.57 (2.30, 2.84)	4.82 (4.30, 5.32)
Jamaica	1.71 (1.58, 1.84)	4.14 (3.80, 4.49)
Japan	1.80 (1.62, 1.99)	3.18 (2.86, 3.51)
Jordan	1.02 (0.93, 1.12)	2.38 (2.15, 2.61)
Kazakhstan	1.39 (1.26, 1.53)	2.87 (2.60, 3.16)
Kenya	0.39 (0.34, 0.43)	0.72 (0.64, 0.81)
Kiribati	2.23 (2.03, 2.46)	4.41 (4.02, 4.87)

Location	PAF 1990<br>% (95% UI)	PAF 2019<br>% (95% UI)
Kuwait	1.56 (1.39, 1.73)	3.91 (3.47, 4.43)
Kyrgyzstan	0.74 (0.66, 0.82)	1.24 (1.11, 1.39)
Lao People's Democratic Republic	0.98 (0.88, 1.07)	2.01 (1.82, 2.24)
Latvia	1.43 (1.30, 1.57)	3.01 (2.73, 3.30)
Lebanon	1.54 (1.40, 1.70)	3.58 (3.23, 3.98)
Lesotho	0.78 (0.70, 0.88)	1.60 (1.44, 1.77)
Liberia	0.85 (0.77, 0.95)	1.16 (1.04, 1.29)
Libya	1.12 (1.00, 1.24)	3.74 (3.34, 4.17)
Lithuania	1.25 (1.14, 1.37)	2.34 (2.12, 2.56)
Luxembourg	1.18 (1.06, 1.31)	4.37 (3.96, 4.82)
Madagascar	0.52 (0.46, 0.58)	0.73 (0.66, 0.82)
Malawi	0.62 (0.55, 0.69)	0.89 (0.79, 0.99)
Malaysia	1.22 (1.13, 1.32)	2.70 (2.44, 2.97)
Maldives	0.68 (0.61, 0.75)	1.70 (1.52, 1.88)
Mali	0.53 (0.48, 0.59)	0.75 (0.67, 0.83)
Malta	2.27 (2.05, 2.50)	4.96 (4.49, 5.42)
Marshall Islands	2.43 (2.19, 2.70)	6.90 (6.30, 7.64)
Mauritania	0.43 (0.38, 0.48)	0.69 (0.62, 0.77)
Mauritius	1.93 (1.79, 2.07)	6.62 (6.07, 7.12)
Mexico	2.08 (1.91, 2.25)	4.37 (4.02, 4.69)
Micronesia (Federated States of)	1.43 (1.30, 1.57)	4.58 (4.19, 5.05)
Monaco	1.90 (1.69, 2.12)	3.84 (3.46, 4.27)
Mongolia	0.37 (0.32, 0.42)	0.87 (0.77, 1.00)
Montenegro	1.97 (1.78, 2.16)	4.48 (4.05, 4.90)
Morocco	0.97 (0.86, 1.08)	3.01 (2.70, 3.34)
Mozambique	0.58 (0.52, 0.64)	0.81 (0.73, 0.89)
Myanmar	1.13 (1.02, 1.25)	2.49 (2.31, 2.69)
Namibia	0.89 (0.80, 0.99)	1.37 (1.23, 1.52)
Nauru	1.47 (1.33, 1.63)	3.09 (2.80, 3.42)
Nepal	0.81 (0.72, 0.91)	2.08 (1.87, 2.31)
Netherlands	1.57 (1.41, 1.71)	2.73 (2.44, 3.01)
New Zealand	0.94 (0.82, 1.06)	2.22 (2.01, 2.43)
Nicaragua	1.11 (1.02, 1.23)	2.81 (2.58, 3.05)
Niger	0.21 (0.18, 0.24)	0.46 (0.41, 0.51)
Nigeria	0.49 (0.43, 0.55)	0.56 (0.50, 0.63)
Niue	3.63 (3.31, 3.99)	8.77 (8.10, 9.52)
North Macedonia	1.86 (1.67, 2.04)	4.89 (4.44, 5.37)
Northern Mariana Islands	1.82 (1.64, 2.05)	6.07 (5.53, 6.66)
Norway	2.30 (2.07, 2.54)	3.49 (3.14, 3.85)
Oman	0.89 (0.80, 1.00)	1.97 (1.74, 2.24)
Pakistan	1.02 (0.91, 1.14)	1.77 (1.59, 1.99)
Palau	2.45 (2.24, 2.70)	7.68 (7.05, 8.46)

Location	PAF 1990<br>% (95% UI)	PAF 2019<br>% (95% UI)
Palestine	0.98 (0.89, 1.09)	2.13 (1.92, 2.32)
Panama	1.45 (1.32, 1.60)	3.55 (3.23, 3.86)
Papua New Guinea	1.76 (1.59, 1.95)	3.18 (2.89, 3.50)
Paraguay	0.87 (0.78, 0.95)	2.00 (1.82, 2.20)
Peru	0.59 (0.53, 0.64)	1.59 (1.44, 1.74)
Philippines	0.90 (0.82, 0.99)	1.54 (1.41, 1.67)
Poland	2.14 (1.95, 2.33)	4.23 (3.84, 4.61)
Portugal	2.48 (2.22, 2.74)	5.45 (4.92, 6.01)
Puerto Rico	2.97 (2.72, 3.26)	7.08 (6.52, 7.65)
Qatar	1.57 (1.38, 1.78)	4.38 (3.93, 4.92)
Republic of Korea	1.19 (1.09, 1.29)	3.44 (3.16, 3.75)
Republic of Moldova	1.52 (1.37, 1.66)	2.90 (2.63, 3.20)
Romania	1.45 (1.30, 1.62)	3.09 (2.76, 3.42)
Russian Federation	1.13 (1.01, 1.24)	1.94 (1.75, 2.13)
Rwanda	0.54 (0.49, 0.61)	0.77 (0.69, 0.85)
Saint Kitts and Nevis	2.51 (2.30, 2.73)	4.51 (4.12, 4.96)
Saint Lucia	2.64 (2.43, 2.87)	6.22 (5.73, 6.74)
Saint Vincent and the Grenadines	2.19 (2.02, 2.38)	5.48 (5.05, 5.94)
Samoa	1.84 (1.68, 2.01)	3.98 (3.63, 4.38)
San Marino	1.53 (1.36, 1.73)	3.41 (3.05, 3.81)
Sao Tome and Principe	0.71 (0.63, 0.79)	1.16 (1.03, 1.29)
Saudi Arabia	1.16 (1.06, 1.28)	3.22 (2.88, 3.63)
Senegal	0.78 (0.70, 0.86)	1.38 (1.24, 1.53)
Serbia	2.36 (2.13, 2.61)	4.87 (4.39, 5.29)
Seychelles	1.51 (1.37, 1.66)	4.88 (4.43, 5.36)
Sierra Leone	0.32 (0.28, 0.35)	0.57 (0.51, 0.63)
Singapore	2.03 (1.86, 2.19)	3.56 (3.22, 3.90)
Slovakia	1.69 (1.52, 1.87)	3.30 (2.97, 3.63)
Slovenia	2.01 (1.82, 2.21)	3.74 (3.39, 4.11)
Solomon Islands	1.31 (1.18, 1.46)	3.15 (2.88, 3.46)
Somalia	0.51 (0.45, 0.57)	0.69 (0.62, 0.78)
South Africa	1.02 (0.92, 1.13)	2.24 (2.05, 2.43)
South Sudan	0.50 (0.45, 0.56)	0.75 (0.67, 0.84)
Spain	2.56 (2.35, 2.76)	4.45 (4.06, 4.93)
Sri Lanka	1.35 (1.23, 1.47)	4.89 (4.49, 5.31)
Sudan	0.88 (0.78, 0.98)	1.83 (1.64, 2.06)
Suriname	2.05 (1.86, 2.24)	5.06 (4.66, 5.49)
Sweden	1.96 (1.74, 2.19)	3.32 (2.96, 3.67)
Switzerland	2.00 (1.81, 2.19)	3.38 (3.04, 3.79)
Syrian Arab Republic	0.96 (0.87, 1.07)	2.91 (2.60, 3.23)
Taiwan (Province of China)	1.49 (1.39, 1.61)	3.62 (3.43, 3.82)
Tajikistan	0.70 (0.64, 0.78)	1.76 (1.58, 1.94)

Location	PAF 1990<br>% (95% UI)	PAF 2019<br>% (95% UI)
Thailand	1.01 (0.92, 1.11)	3.14 (2.88, 3.43)
Timor-Leste	0.53 (0.47, 0.59)	1.50 (1.36, 1.65)
Togo	0.31 (0.27, 0.35)	0.67 (0.60, 0.75)
Tokelau	2.22 (2.02, 2.44)	4.78 (4.37, 5.27)
Tonga	1.92 (1.75, 2.11)	3.91 (3.59, 4.27)
Trinidad and Tobago	2.99 (2.72, 3.23)	6.46 (5.97, 7.03)
Tunisia	1.31 (1.18, 1.45)	4.21 (3.79, 4.66)
Turkey	1.11 (1.01, 1.21)	2.64 (2.38, 2.89)
Turkmenistan	0.72 (0.64, 0.80)	1.97 (1.78, 2.16)
Tuvalu	2.32 (2.11, 2.56)	4.87 (4.42, 5.37)
Uganda	0.57 (0.50, 0.63)	0.82 (0.73, 0.92)
Ukraine	1.46 (1.30, 1.63)	2.27 (2.05, 2.51)
United Arab Emirates	1.11 (0.97, 1.25)	3.59 (3.14, 4.08)
United Kingdom	2.37 (2.15, 2.62)	5.46 (4.97, 6.01)
United Republic of Tanzania	0.36 (0.31, 0.40)	0.69 (0.62, 0.76)
United States of America	2.55 (2.33, 2.77)	5.02 (4.68, 5.38)
United States Virgin Islands	1.96 (1.77, 2.17)	5.85 (5.33, 6.46)
Uruguay	0.58 (0.52, 0.65)	2.20 (1.96, 2.42)
Uzbekistan	0.62 (0.56, 0.69)	1.93 (1.76, 2.10)
Vanuatu	1.46 (1.31, 1.63)	3.35 (3.05, 3.68)
Venezuela (Bolivarian Republic of)	1.34 (1.22, 1.46)	3.56 (3.27, 3.86)
Viet Nam	0.78 (0.71, 0.86)	2.12 (1.94, 2.30)
Yemen	0.53 (0.47, 0.60)	1.11 (0.98, 1.25)
Zambia	0.53 (0.47, 0.59)	0.77 (0.69, 0.86)
Zimbabwe	0.72 (0.64, 0.79)	1.46 (1.31, 1.61)

T2DM type 2 diabetes mellitus, HBV hepatitis B virus, PAF population attributable fraction, SDI socio-demographic index, UI uncertainty interval.

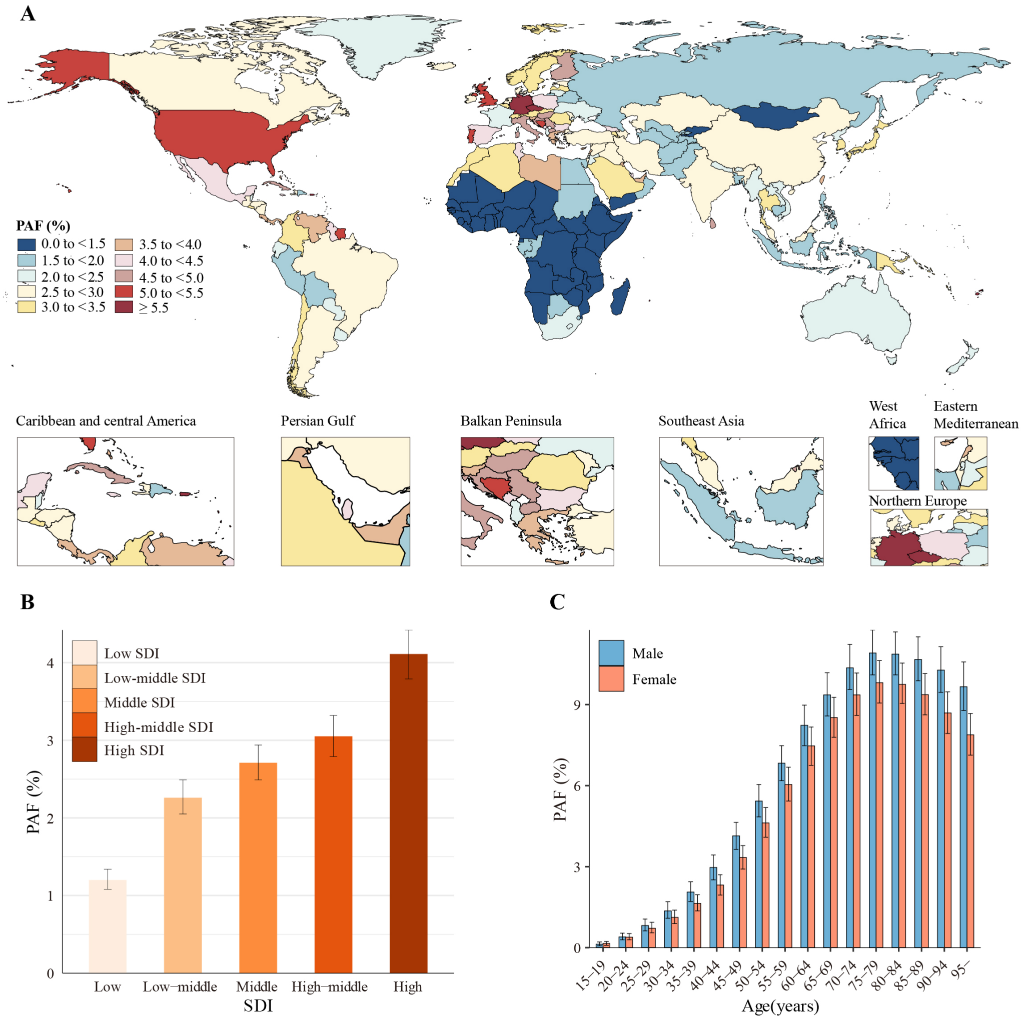

Figure S1. PAF of HBV–T2DM comorbidity on liver cancer in 2019 stratified by country (A), SDI (B), and age and sex (C). HBV hepatitis B virus, PAF population attributable fraction, SDI socio-demographic index, T2DM type 2 diabetes mellitus

Table S3. Age-standardized prevalence, DALY, and mortality rates (per 10,000,000 individuals) of liver cancer due to HBV–T2DM comorbidity in 1990 and 2019 and EAPCs from 1990 to 2019, stratified by country

Country	Age-standardized prevalence rate			Age-standardized DALY rate			Age-standardized mortality rate		
	1990 No. (95% UI)	2019 No. (95% UI)	EAPC % (95% CI)	1990 No. (95% UI)	2019 No. (95% UI)	EAPC % (95% CI)	1990 No. (95% UI)	2019 No. (95% UI)	EAPC % (95% CI)
Afghanistan	9.9 (7.0, 13.7)	6.8 (4.7, 9.4)	1.58 (1.44, 1.72)	286.4 (201.7, 392.5)	190.1 (133.0, 268.3)	1.49 (1.35, 1.64)	9.2 (6.5, 12.8)	6.2 (4.3, 8.5)	1.51 (1.37, 1.65)
Albania	3.2 (2.4, 4.2)	4.1 (2.7, 6.1)	-1.61 (-2.20, -1.02)	83.8 (63.0, 108.8)	99.8 (65.1, 147.5)	-1.96 (-2.58, -1.33)	3.0 (2.2, 4.0)	3.5 (2.3, 5.3)	-2.08 (-2.74, -1.42)
Algeria	1.2 (0.9, 1.6)	2.7 (1.9, 3.8)	3.28 (3.12, 3.45)	31.8 (22.8, 42.3)	56.7 (39.7, 78.9)	2.53 (2.34, 2.71)	1.2 (0.8, 1.6)	2.1 (1.4, 3.0)	2.67 (2.46, 2.88)
American Samoa	16.5 (11.5, 21.9)	33.4 (25.4, 44.2)	3.07 (2.76, 3.38)	450.1 (310.9, 596.8)	886.3 (670.6, 1179.2)	2.99 (2.68, 3.30)	15.1 (10.5, 19.9)	29.0 (22.1, 37.7)	2.91 (2.59, 3.22)
Andorra	1.9 (1.2, 3.0)	8.3 (5.2, 12.4)	3.52 (3.33, 3.72)	35.0 (21.0, 53.7)	93.9 (58.4, 143.6)	1.79 (1.71, 1.87)	1.3 (0.8, 2.0)	3.4 (2.1, 5.3)	1.79 (1.68, 1.91)
Angola	1.2 (0.8, 1.7)	0.7 (0.4, 0.9)	0.48 (0.43, 0.52)	34.0 (23.3, 48.0)	18.3 (12.3, 26.4)	0.40 (0.33, 0.47)	1.1 (0.8, 1.6)	0.6 (0.4, 0.9)	0.39 (0.32, 0.46)
Antigua and Barbuda	7.0 (5.2, 9.3)	3.5 (2.5, 4.9)	-3.42 (-4.39, -2.43)	182.5 (138.4, 240.5)	84.6 (61.5, 116.5)	-3.58 (-4.56, -2.59)	6.4 (4.6, 8.6)	3.1 (2.2, 4.4)	-3.41 (-4.39, -2.42)
Argentina	0.4 (0.3, 0.5)	1.1 (0.7, 1.6)	3.62 (3.49, 3.75)	9.3 (6.5, 13.1)	24.4 (17.2, 34.1)	3.27 (3.13, 3.41)	0.3 (0.2, 0.5)	0.9 (0.6, 1.3)	3.49 (3.33, 3.64)
Armenia	0.4 (0.3, 0.5)	5.0 (3.4, 7.1)	11.24 (9.58, 12.93)	9.6 (7.2, 12.5)	126.6 (86.0, 177.2)	11.28 (9.55, 13.03)	0.3 (0.2, 0.5)	4.6 (3.1, 6.7)	11.32 (9.58, 13.10)
Australia	0.4 (0.3, 0.5)	2.5 (1.6, 3.8)	5.16 (4.44, 5.89)	8.3 (6.0, 11.3)	39.3 (27.9, 55.2)	3.92 (3.51, 4.33)	0.3 (0.2, 0.4)	1.4 (0.9, 2.0)	4.05 (3.62, 4.48)

Country	Age-standardized prevalence rate			Age-standardized DALY rate			Age-standardized mortality rate		
	1990<br>No. (95% UI)	2019<br>No. (95% UI)	EAPC<br>% (95% CI)	1990<br>No. (95% UI)	2019<br>No. (95% UI)	EAPC<br>% (95% CI)	1990<br>No. (95% UI)	2019<br>No. (95% UI)	EAPC<br>% (95% CI)
Austria	0.4 (0.3, 0.6)	2.4 (1.5, 3.6)	4.17 (3.51, 4.83)	7.8 (5.4, 10.9)	26.2 (17.5, 39.1)	2.26 (1.82, 2.71)	0.3 (0.2, 0.4)	1.0 (0.6, 1.5)	2.74 (2.29, 3.19)
Azerbaijan	0.3 (0.2, 0.4)	2.6 (1.7, 3.7)	8.41 (6.12, 10.76)	8.2 (6.1, 10.9)	66.7 (45.2, 95.5)	8.53 (6.05, 11.06)	0.3 (0.2, 0.4)	2.4 (1.6, 3.6)	8.62 (6.18, 11.11)
Bahamas	6.6 (4.9, 8.8)	4.0 (2.9, 5.6)	-2.43 (-3.27, -1.58)	178.6 (134.7, 235.8)	105.1 (75.2, 145.1)	-2.54 (-3.41, -1.66)	5.9 (4.3, 8.0)	3.4 (2.4, 4.8)	-2.52 (-3.38, -1.66)
Bahrain	6.5 (4.7, 9.0)	11.5 (7.9, 16.5)	1.21 (0.65, 1.78)	161.5 (118.1, 221.1)	196.5 (136.4, 281.2)	-0.23 (-0.91, 0.44)	6.5 (4.6, 9.1)	8.2 (5.6, 11.8)	-0.05 (-0.70, 0.60)
Bangladesh	1.5 (1.1, 2.1)	1.3 (0.9, 1.8)	-0.91 (-1.12, -0.70)	43.7 (31.5, 59.3)	34.4 (24.3, 47.7)	-1.18 (-1.44, -0.92)	1.2 (0.9, 1.7)	1.1 (0.7, 1.6)	-0.66 (-0.87, -0.46)
Barbados	2.2 (1.4, 3.3)	3.7 (2.7, 5.2)	-0.03 (-0.28, 0.23)	57.5 (36.4, 84.6)	88.8 (63.1, 123.3)	-0.34 (-0.63, -0.06)	2.0 (1.2, 3.0)	3.2 (2.3, 4.6)	-0.18 (-0.44, 0.09)
Belarus	0.4 (0.3, 0.5)	1.2 (0.8, 1.9)	3.53 (2.70, 4.37)	8.8 (6.6, 11.7)	27.4 (16.6, 42.4)	3.33 (2.29, 4.38)	0.3 (0.2, 0.4)	0.9 (0.6, 1.4)	3.04 (2.09, 3.99)
Belgium	0.6 (0.5, 0.8)	3.4 (2.2, 4.9)	3.80 (3.60, 4.00)	12.7 (9.2, 17.0)	42.8 (30.4, 60.1)	2.14 (1.87, 2.41)	0.5 (0.3, 0.6)	1.6 (1.1, 2.2)	2.12 (1.80, 2.43)
Belize	3.1 (2.3, 4.2)	2.1 (1.5, 2.9)	-0.99 (-1.45, -0.52)	83.2 (62.4, 107.5)	54.8 (40.5, 73.8)	-1.09 (-1.56, -0.61)	2.9 (2.1, 3.9)	1.9 (1.4, 2.6)	-0.99 (-1.47, -0.52)
Benin	4.0 (2.9, 5.4)	2.0 (1.4, 2.8)	-0.02 (-0.23, 0.18)	110.4 (80.5, 147.2)	54.4 (37.1, 76.1)	-0.05 (-0.28, 0.18)	4.0 (2.9, 5.3)	1.9 (1.3, 2.7)	-0.05 (-0.27, 0.18)
Bermuda	3.2 (2.3, 4.3)	2.8 (1.9, 3.8)	-2.44 (-3.54, -1.32)	80.0 (58.3, 107.1)	50.8 (35.1, 70.9)	-3.55 (-4.72, -2.36)	2.9 (2.1, 4.1)	2.0 (1.3, 2.8)	-3.29 (-4.43, -2.13)

Country	Age-standardized prevalence rate			Age-standardized DALY rate			Age-standardized mortality rate		
	1990 No. (95% UI)	2019 No. (95% UI)	EAPC % (95% CI)	1990 No. (95% UI)	2019 No. (95% UI)	EAPC % (95% CI)	1990 No. (95% UI)	2019 No. (95% UI)	EAPC % (95% CI)
Bhutan	1.0 (0.6, 1.6)	1.6 (0.9, 2.5)	2.33 (2.22, 2.43)	27.7 (15.7, 45.4)	41.7 (24.2, 65.5)	2.06 (1.96, 2.17)	0.9 (0.5, 1.5)	1.5 (0.9, 2.3)	2.49 (2.36, 2.62)
Bolivia (Plurinational State of)	3.6 (2.6, 4.9)	3.5 (2.4, 4.9)	0.56 (0.46, 0.67)	96.9 (69.2, 131.0)	89.5 (61.8, 123.8)	0.34 (0.23, 0.44)	3.6 (2.6, 4.9)	3.6 (2.5, 5.0)	0.63 (0.53, 0.73)
Bosnia and Herzegovina	2.8 (2.0, 3.7)	10.1 (6.7, 15.0)	3.27 (2.82, 3.71)	73.1 (54.0, 96.8)	243.3 (162.6, 357.7)	2.89 (2.45, 3.33)	2.6 (1.9, 3.5)	9.3 (6.1, 13.9)	3.19 (2.71, 3.68)
Botswana	0.6 (0.2, 1.6)	1.0 (0.7, 1.5)	2.54 (1.67, 3.43)	17.0 (7.1, 45.3)	29.2 (18.9, 43.9)	2.56 (1.57, 3.55)	0.5 (0.2, 1.4)	0.9 (0.6, 1.3)	2.38 (1.40, 3.37)
Brazil	0.9 (0.8, 1.1)	1.2 (1.0, 1.4)	1.15 (0.93, 1.38)	25.4 (22.0, 29.0)	30.8 (26.3, 35.7)	0.85 (0.64, 1.06)	0.8 (0.7, 1.0)	1.1 (0.9, 1.3)	1.10 (0.88, 1.32)
Brunei Darussalam	23.0 (17.0, 30.4)	32.0 (24.0, 41.5)	2.59 (1.97, 3.21)	574.4 (427.4, 751.6)	687.2 (516.9, 880.7)	2.02 (1.41, 2.64)	20.7 (15.2, 27.4)	23.4 (17.5, 30.5)	1.87 (1.21, 2.53)
Bulgaria	2.9 (2.1, 3.8)	4.9 (3.3, 7.1)	0.29 (-0.14, 0.72)	76.6 (57.2, 100.8)	126.0 (84.9, 184.6)	0.12 (-0.38, 0.61)	2.6 (1.9, 3.4)	4.1 (2.8, 6.1)	0.07 (-0.43, 0.58)
Burkina Faso	1.6 (1.1, 2.1)	0.8 (0.5, 1.0)	-0.07 (-0.23, 0.08)	43.3 (31.3, 56.7)	20.8 (14.6, 28.6)	-0.09 (-0.28, 0.10)	1.6 (1.1, 2.1)	0.7 (0.5, 1.0)	-0.25 (-0.41, -0.08)
Burundi	1.2 (0.8, 1.8)	0.6 (0.3, 1.0)	-0.64 (-0.83, -0.44)	36.5 (23.8, 52.5)	16.4 (9.6, 27.4)	-0.81 (-1.03, -0.59)	1.2 (0.8, 1.7)	0.5 (0.3, 0.9)	-0.71 (-0.94, -0.49)
Cabo Verde	0.7 (0.5, 0.8)	11.3 (8.5, 14.6)	9.11 (6.48, 11.80)	17.4 (13.7, 21.7)	298.9 (225.2, 393.0)	9.32 (6.43, 12.30)	0.7 (0.5, 0.8)	10.3 (7.8, 13.4)	8.78 (6.08, 11.55)
Cambodia	5.0 (3.7, 6.6)	6.9 (4.9, 9.6)	1.71 (1.57, 1.85)	148.5 (111.3, 194.3)	191.0 (136.7, 264.8)	1.49 (1.34, 1.65)	4.3 (3.2, 5.8)	5.9 (4.1, 8.2)	1.67 (1.51, 1.82)

Country	Age-standardized prevalence rate			Age-standardized DALY rate			Age-standardized mortality rate		
	1990 No. (95% UI)	2019 No. (95% UI)	EAPC % (95% CI)	1990 No. (95% UI)	2019 No. (95% UI)	EAPC % (95% CI)	1990 No. (95% UI)	2019 No. (95% UI)	EAPC % (95% CI)
Cameroon	0.3 (0.2, 0.5)	0.3 (0.2, 0.4)	1.62 (1.18, 2.05)	9.3 (6.0, 14.8)	7.5 (5.0, 10.8)	1.65 (1.19, 2.11)	0.3 (0.2, 0.5)	0.3 (0.2, 0.4)	1.49 (1.02, 1.97)
Canada	0.2 (0.1, 0.3)	1.8 (1.1, 2.7)	6.37 (6.17, 6.58)	3.2 (2.3, 4.6)	19.4 (13.5, 28.1)	4.43 (4.13, 4.73)	0.1 (0.1, 0.2)	0.7 (0.5, 1.0)	4.67 (4.38, 4.96)
Central African Republic	1.8 (1.1, 2.8)	1.2 (0.7, 1.9)	0.22 (-0.06, 0.51)	55.2 (34.0, 82.4)	34.1 (19.2, 55.9)	0.07 (-0.22, 0.36)	1.8 (1.1, 2.6)	1.1 (0.6, 1.8)	0.17 (-0.15, 0.49)
Chad	3.8 (2.6, 5.1)	2.0 (1.4, 2.7)	0.85 (0.64, 1.05)	104.0 (72.7, 141.2)	54.0 (38.0, 73.8)	0.77 (0.54, 1.01)	3.7 (2.6, 5.0)	2.0 (1.4, 2.7)	0.87 (0.62, 1.11)
Chile	0.7 (0.5, 1.0)	2.1 (1.4, 3.2)	3.46 (3.20, 3.73)	18.7 (13.5, 26.1)	43.2 (30.3, 60.3)	2.67 (2.42, 2.92)	0.7 (0.5, 0.9)	1.7 (1.1, 2.4)	2.94 (2.69, 3.20)
China	32.2 (26.2, 38.8)	28.1 (22.6, 34.3)	-3.02 (-3.79, -2.24)	898.7 (743.5, 1084.7)	522.6 (424.0, 642.1)	-4.45 (-5.20, -3.69)	28.0 (23.3, 33.7)	16.9 (13.8, 20.7)	-4.26 (-4.98, -3.54)
Colombia	1.6 (1.2, 2.1)	1.8 (1.1, 2.6)	-0.17 (-0.58, 0.24)	42.7 (32.1, 56.4)	40.6 (25.9, 61.0)	-0.77 (-1.18, -0.35)	1.5 (1.1, 2.0)	1.5 (1.0, 2.3)	-0.38 (-0.75, 0.00)
Comoros	1.1 (0.5, 2.0)	0.9 (0.5, 1.5)	-0.02 (-0.23, 0.20)	29.9 (12.1, 57.2)	24.2 (13.6, 42.0)	-0.07 (-0.32, 0.18)	1.0 (0.5, 1.9)	0.8 (0.5, 1.4)	-0.01 (-0.19, 0.18)
Congo	2.0 (1.3, 3.0)	1.1 (0.7, 1.7)	-0.77 (-0.95, -0.60)	58.5 (37.2, 89.9)	30.8 (19.8, 47.3)	-0.92 (-1.13, -0.70)	1.9 (1.2, 2.8)	1.0 (0.7, 1.6)	-0.75 (-0.96, -0.55)
Cook Islands	24.8 (18.7, 32.2)	48.7 (36.0, 65.2)	1.52 (1.42, 1.62)	659.1 (496.7, 863.9)	1131.5 (823.2, 1514.0)	1.13 (0.99, 1.26)	22.2 (16.6, 28.9)	37.2 (27.5, 49.4)	0.98 (0.87, 1.09)
Costa Rica	2.3 (1.7, 3.1)	3.5 (2.3, 5.3)	0.68 (0.10, 1.25)	58.9 (43.1, 78.8)	79.3 (51.7, 119.3)	0.19 (-0.39, 0.78)	2.0 (1.4, 2.9)	2.7 (1.7, 4.2)	0.21 (-0.36, 0.78)

Country	Age-standardized prevalence rate			Age-standardized DALY rate			Age-standardized mortality rate		
	1990 No. (95% UI)	2019 No. (95% UI)	EAPC % (95% CI)	1990 No. (95% UI)	2019 No. (95% UI)	EAPC % (95% CI)	1990 No. (95% UI)	2019 No. (95% UI)	EAPC % (95% CI)
Croatia	1.5 (1.1, 2.0)	5.1 (3.4, 7.5)	2.72 (2.33, 3.12)	35.1 (25.8, 47.0)	93.2 (61.9, 138.1)	1.39 (0.85, 1.93)	1.3 (0.9, 1.7)	3.5 (2.3, 5.2)	1.45 (0.91, 2.00)
Cuba	5.0 (3.8, 6.6)	3.3 (2.3, 4.8)	-3.43 (-4.87, -1.96)	127.6 (97.7, 164.9)	72.8 (50.1, 104.6)	-3.96 (-5.48, -2.41)	4.6 (3.4, 6.1)	2.8 (1.9, 4.1)	-3.57 (-5.10, -2.02)
Cyprus	1.2 (0.9, 1.8)	3.0 (2.1, 4.3)	1.80 (1.59, 2.01)	29.5 (20.3, 42.1)	39.5 (26.9, 56.5)	-0.65 (-0.81, -0.48)	1.1 (0.7, 1.6)	1.5 (1.0, 2.2)	-0.41 (-0.61, -0.21)
Czechia	2.4 (1.7, 3.2)	4.5 (3.0, 6.6)	0.14 (-0.07, 0.36)	61.8 (45.7, 83.8)	99.5 (67.4, 145.4)	-0.54 (-0.77, -0.31)	2.2 (1.6, 3.0)	3.7 (2.5, 5.4)	-0.34 (-0.56, -0.11)
Cote d'Ivoire	4.7 (3.3, 6.3)	2.2 (1.5, 3.1)	-1.23 (-1.59, -0.86)	131.2 (92.2, 175.8)	58.8 (39.4, 84.0)	-1.26 (-1.67, -0.85)	4.7 (3.3, 6.3)	2.1 (1.4, 3.0)	-1.20 (-1.60, -0.80)
Democratic People's Republic of Korea	13.1 (9.6, 17.9)	17.4 (11.9, 24.3)	-0.04 (-0.16, 0.08)	369.0 (265.0, 503.3)	471.4 (319.2, 664.8)	-0.19 (-0.33, -0.05)	11.3 (8.2, 15.1)	14.3 (10.0, 19.9)	-0.18 (-0.31, -0.05)
Democratic Republic of the Congo	1.1 (0.7, 1.5)	0.6 (0.4, 1.0)	0.59 (0.41, 0.77)	30.9 (21.2, 43.0)	18.7 (12.2, 28.1)	0.57 (0.39, 0.75)	1.0 (0.7, 1.4)	0.6 (0.4, 0.9)	0.42 (0.23, 0.60)
Denmark	0.5 (0.4, 0.8)	3.3 (2.1, 4.9)	4.99 (4.71, 5.28)	6.5 (4.6, 9.1)	36.4 (25.0, 51.2)	4.50 (4.18, 4.83)	0.2 (0.2, 0.3)	1.3 (0.9, 1.9)	4.63 (4.30, 4.95)
Djibouti	1.2 (0.6, 2.3)	1.0 (0.6, 1.8)	1.05 (0.83, 1.26)	32.7 (17.7, 64.1)	27.7 (14.9, 49.4)	1.02 (0.79, 1.25)	1.1 (0.6, 2.1)	1.0 (0.5, 1.7)	1.13 (0.92, 1.35)
Dominica	7.3 (5.4, 9.9)	5.3 (3.6, 7.5)	-2.00 (-2.65, -1.35)	195.6 (144.2, 263.9)	138.9 (94.4, 194.3)	-2.13 (-2.83, -1.42)	6.7 (4.8, 9.1)	4.8 (3.2, 6.9)	-2.02 (-2.74, -1.30)
Dominican Republic	1.5 (1.1, 1.9)	2.7 (1.6, 4.6)	2.86 (2.19, 3.54)	41.0 (30.8, 53.1)	73.2 (42.0, 123.7)	2.69 (1.97, 3.42)	1.3 (1.0, 1.8)	2.4 (1.4, 4.0)	2.82 (2.14, 3.51)

Country	Age-standardized prevalence rate			Age-standardized DALY rate			Age-standardized mortality rate		
	1990<br>No. (95% UI)	2019<br>No. (95% UI)	EAPC<br>% (95% CI)	1990<br>No. (95% UI)	2019<br>No. (95% UI)	EAPC<br>% (95% CI)	1990<br>No. (95% UI)	2019<br>No. (95% UI)	EAPC<br>% (95% CI)
Ecuador	2.1 (1.6, 2.5)	3.4 (2.4, 4.6)	2.53 (2.30, 2.75)	54.1 (43.7, 64.9)	81.4 (57.6, 109.8)	2.08 (1.85, 2.32)	2.0 (1.6, 2.5)	3.4 (2.4, 4.6)	2.52 (2.28, 2.77)
Egypt	2.9 (1.9, 4.3)	5.6 (3.4, 9.0)	4.10 (3.66, 4.53)	81.2 (54.3, 119.7)	144.0 (86.0, 227.9)	3.75 (3.37, 4.13)	2.6 (1.7, 3.8)	4.7 (2.8, 7.5)	3.89 (3.49, 4.30)
El Salvador	1.3 (0.9, 1.7)	1.1 (0.7, 1.7)	-0.57 (-1.14, 0.02)	34.8 (26.0, 46.1)	27.8 (17.5, 42.6)	-0.96 (-1.63, -0.29)	1.1 (0.8, 1.6)	1.0 (0.6, 1.5)	-0.57 (-1.14, 0.01)
Equatorial Guinea	1.4 (0.9, 2.2)	0.9 (0.5, 1.5)	1.18 (0.94, 1.43)	42.9 (28.0, 63.6)	25.3 (13.3, 41.3)	0.92 (0.63, 1.21)	1.3 (0.9, 2.0)	0.9 (0.5, 1.4)	1.29 (1.03, 1.55)
Eritrea	1.2 (0.7, 1.9)	0.8 (0.5, 1.2)	0.31 (0.09, 0.53)	35.9 (22.1, 56.7)	21.9 (13.0, 34.3)	0.21 (-0.01, 0.42)	1.2 (0.7, 1.9)	0.7 (0.4, 1.1)	0.26 (0.02, 0.49)
Estonia	0.7 (0.5, 0.9)	2.7 (1.8, 4.0)	1.78 (1.13, 2.44)	17.8 (13.3, 23.6)	54.4 (34.8, 80.8)	0.85 (-0.02, 1.72)	0.6 (0.4, 0.8)	1.9 (1.2, 2.9)	1.21 (0.36, 2.07)
Eswatini	3.8 (2.1, 8.3)	13.0 (3.1, 25.0)	7.29 (5.62, 8.99)	109.8 (60.7, 239.7)	383.5 (89.5, 744.5)	7.56 (5.74, 9.41)	3.6 (1.9, 7.6)	11.7 (2.9, 22.1)	7.33 (5.52, 9.17)
Ethiopia	0.9 (0.6, 1.3)	0.3 (0.2, 0.5)	-1.27 (-1.48, -1.06)	25.4 (16.8, 37.9)	9.1 (6.7, 12.2)	-1.59 (-1.82, -1.36)	0.8 (0.6, 1.2)	0.3 (0.2, 0.4)	-1.23 (-1.45, -1.00)
Fiji	13.1 (9.7, 17.0)	21.4 (15.4, 29.6)	2.56 (2.19, 2.92)	360.7 (270.1, 467.8)	583.2 (417.7, 803.9)	2.55 (2.14, 2.96)	12.2 (9.0, 16.0)	19.3 (13.8, 26.6)	2.38 (2.02, 2.73)
Finland	1.3 (0.9, 1.8)	8.0 (5.3, 12.2)	4.95 (4.71, 5.20)	19.3 (13.8, 26.3)	64.4 (45.6, 90.2)	2.77 (2.52, 3.02)	0.7 (0.5, 0.9)	2.4 (1.6, 3.5)	3.11 (2.89, 3.33)
France	0.7 (0.5, 1.0)	3.4 (2.2, 5.1)	3.52 (3.28, 3.76)	14.7 (10.3, 20.6)	43.3 (29.3, 62.3)	1.42 (1.20, 1.64)	0.5 (0.3, 0.7)	1.6 (1.0, 2.3)	1.49 (1.24, 1.73)

Country	Age-standardized prevalence rate			Age-standardized DALY rate			Age-standardized mortality rate		
	1990	2019	EAPC	1990	2019	EAPC	1990	2019	EAPC
	No. (95% UI)	No. (95% UI)	% (95% CI)	No. (95% UI)	No. (95% UI)	% (95% CI)	No. (95% UI)	No. (95% UI)	% (95% CI)
Gabon	1.5 (1.0, 2.1)	1.4 (0.8, 2.3)	1.03 (0.76, 1.29)	43.1 (27.9, 61.8)	39.7 (23.1, 62.8)	0.88 (0.58, 1.18)	1.3 (0.9, 1.9)	1.3 (0.8, 2.1)	1.09 (0.77, 1.41)
Gambia	18.4 (13.0, 25.3)	18.5 (12.8, 26.3)	2.13 (1.94, 2.31)	522.1 (367.7, 719.0)	523.3 (357.1, 741.7)	2.16 (1.94, 2.38)	17.1 (12.1, 23.1)	17.0 (11.9, 23.6)	2.14 (1.94, 2.35)
Georgia	0.4 (0.3, 0.5)	4.4 (3.1, 6.2)	6.63 (5.13, 8.16)	9.9 (7.3, 13.4)	120.9 (84.0, 170.3)	7.05 (5.58, 8.54)	0.4 (0.3, 0.5)	3.8 (2.6, 5.4)	6.50 (5.12, 7.90)
Germany	0.9 (0.7, 1.3)	6.0 (3.9, 8.8)	3.82 (3.37, 4.27)	17.1 (12.7, 23.4)	60.2 (42.3, 86.0)	1.83 (1.62, 2.05)	0.6 (0.4, 0.8)	2.3 (1.5, 3.3)	2.07 (1.84, 2.29)
Ghana	3.9 (2.8, 5.4)	3.6 (2.6, 5.0)	1.69 (1.32, 2.07)	107.8 (77.0, 150.2)	99.2 (69.1, 135.0)	1.64 (1.27, 2.02)	3.8 (2.7, 5.3)	3.5 (2.4, 4.7)	1.67 (1.29, 2.04)
Greece	1.2 (1.0, 1.5)	7.6 (5.4, 10.6)	4.57 (4.32, 4.82)	23.0 (17.9, 28.3)	108.1 (83.7, 136.7)	3.54 (3.36, 3.73)	1.0 (0.7, 1.2)	4.3 (3.2, 5.6)	3.36 (3.17, 3.54)
Greenland	0.5 (0.3, 0.7)	2.0 (1.2, 3.0)	4.34 (4.24, 4.43)	13.3 (8.9, 19.9)	48.8 (30.5, 75.6)	3.94 (3.81, 4.07)	0.4 (0.3, 0.6)	1.7 (1.0, 2.5)	4.21 (4.11, 4.31)
Grenada	7.8 (5.8, 10.3)	3.9 (2.8, 5.4)	-3.11 (-4.11, -2.10)	213.4 (161.1, 278.2)	100.1 (71.7, 136.4)	-3.30 (-4.35, -2.25)	7.2 (5.3, 9.6)	3.6 (2.5, 4.9)	-3.18 (-4.17, -2.17)
Guam	5.3 (4.2, 6.8)	16.7 (12.8, 21.1)	4.34 (4.15, 4.54)	139.0 (109.5, 176.0)	429.4 (332.3, 545.8)	4.39 (4.13, 4.65)	4.8 (3.8, 6.1)	13.4 (10.2, 17.0)	3.94 (3.67, 4.22)
Guatemala	3.7 (2.6, 5.1)	2.3 (1.5, 3.5)	-0.98 (-2.17, 0.24)	103.3 (75.9, 138.6)	61.7 (40.8, 90.6)	-1.42 (-2.80, -0.03)	3.4 (2.4, 4.8)	2.1 (1.3, 3.1)	-1.35 (-2.66, -0.03)
Guinea	16.2 (12.4, 20.8)	14.5 (9.6, 20.0)	2.22 (2.12, 2.32)	449.2 (340.0, 578.5)	401.8 (264.6, 558.3)	2.21 (2.10, 2.32)	16.0 (12.1, 20.6)	14.1 (9.4, 19.2)	2.18 (2.06, 2.29)

Country	Age-standardized prevalence rate			Age-standardized DALY rate			Age-standardized mortality rate		
	1990<br>No. (95% UI)	2019<br>No. (95% UI)	EAPC<br>% (95% CI)	1990<br>No. (95% UI)	2019<br>No. (95% UI)	EAPC<br>% (95% CI)	1990<br>No. (95% UI)	2019<br>No. (95% UI)	EAPC<br>% (95% CI)
Guinea-Bissau	5.3 (3.6, 7.8)	2.7 (1.9, 3.9)	0.22 (0.13, 0.31)	149.9 (100.4, 221.5)	74.6 (50.5, 107.7)	0.14 (0.03, 0.25)	5.2 (3.5, 7.4)	2.6 (1.8, 3.6)	0.16 (0.05, 0.27)
Guyana	6.4 (4.7, 8.7)	3.9 (2.6, 5.7)	-1.67 (-2.31, -1.03)	178.8 (132.6, 241.1)	108.1 (72.3, 155.8)	-1.62 (-2.27, -0.97)	5.9 (4.2, 8.1)	3.6 (2.4, 5.2)	-1.52 (-2.17, -0.86)
Haiti	5.3 (3.1, 8.5)	3.4 (1.9, 5.8)	-0.41 (-0.69, -0.13)	148.5 (88.3, 229.7)	93.3 (51.7, 155.0)	-0.45 (-0.72, -0.17)	5.2 (3.1, 8.2)	3.3 (1.9, 5.7)	-0.38 (-0.64, -0.13)
Honduras	6.0 (2.1, 10.2)	7.1 (2.8, 12.1)	1.67 (1.58, 1.77)	163.7 (59.2, 270.4)	183.5 (73.9, 316.1)	1.49 (1.36, 1.62)	5.6 (1.9, 9.7)	6.8 (2.6, 11.7)	1.89 (1.74, 2.03)
Hungary	2.8 (2.1, 3.8)	3.2 (2.2, 4.5)	-0.75 (-1.65, 0.17)	74.4 (55.7, 99.1)	73.2 (50.8, 103.8)	-1.28 (-2.32, -0.23)	2.6 (1.9, 3.5)	2.7 (1.8, 3.9)	-1.09 (-2.10, -0.07)
Iceland	0.5 (0.4, 0.7)	3.2 (2.2, 4.5)	5.15 (4.88, 5.42)	8.8 (6.3, 12.3)	33.6 (23.8, 46.7)	3.42 (3.28, 3.56)	0.3 (0.2, 0.4)	1.2 (0.8, 1.8)	3.51 (3.36, 3.66)
India	1.9 (1.5, 2.3)	2.7 (2.1, 3.3)	1.43 (1.32, 1.54)	52.1 (41.9, 62.3)	71.2 (57.9, 87.3)	1.30 (1.18, 1.41)	1.8 (1.5, 2.2)	2.5 (2.0, 3.1)	1.25 (1.12, 1.38)
Indonesia	0.7 (0.6, 0.8)	0.9 (0.7, 1.1)	0.47 (0.05, 0.88)	19.2 (15.9, 23.0)	22.4 (17.4, 27.9)	0.21 (-0.20, 0.61)	0.6 (0.5, 0.7)	0.8 (0.6, 1.0)	0.62 (0.23, 1.02)
Iran (Islamic Republic of)	2.8 (2.3, 3.3)	5.2 (4.4, 6.1)	2.02 (1.39, 2.65)	68.4 (56.9, 80.0)	94.9 (81.5, 109.3)	1.05 (0.45, 1.66)	2.7 (2.2, 3.1)	3.7 (3.2, 4.3)	1.07 (0.51, 1.64)
Iraq	4.5 (3.1, 6.2)	6.4 (4.3, 9.0)	2.82 (2.46, 3.18)	121.0 (84.0, 167.3)	141.0 (95.5, 201.4)	2.17 (1.85, 2.49)	4.2 (2.9, 5.8)	5.1 (3.5, 7.1)	2.31 (1.95, 2.67)
Ireland	0.2 (0.1, 0.2)	2.7 (1.7, 4.2)	9.89 (9.38, 10.39)	3.7 (2.7, 5.2)	29.5 (20.6, 42.0)	7.16 (6.75, 7.57)	0.1 (0.1, 0.2)	1.1 (0.8, 1.6)	7.40 (6.98, 7.81)

Country	Age-standardized prevalence rate			Age-standardized DALY rate			Age-standardized mortality rate		
	1990	2019	EAPC	1990	2019	EAPC	1990	2019	EAPC
	No. (95% UI)	No. (95% UI)	% (95% CI)	No. (95% UI)	No. (95% UI)	% (95% CI)	No. (95% UI)	No. (95% UI)	% (95% CI)
Israel	0.7 (0.5, 0.9)	1.9 (1.2, 2.8)	2.84 (2.51, 3.16)	15.1 (10.6, 21.2)	29.1 (20.4, 41.3)	1.39 (1.10, 1.68)	0.6 (0.4, 0.8)	1.1 (0.7, 1.5)	1.31 (1.04, 1.59)
Italy	2.3 (1.9, 2.7)	7.8 (5.8, 10.3)	1.61 (1.25, 1.97)	39.6 (33.7, 46.7)	69.5 (57.8, 83.2)	-1.00 (-1.22, -0.78)	1.4 (1.2, 1.7)	2.5 (2.1, 3.1)	-0.95 (-1.18. -0.73)
Jamaica	2.9 (2.2, 3.8)	3.3 (2.3, 4.6)	1.18 (0.34, 2.03)	76.2 (58.1, 98.4)	84.0 (57.4, 117.3)	0.78 (-0.11, 1.68)	2.7 (2.0, 3.5)	2.9 (2.0, 4.1)	0.75 (-0.09, 1.60)
Japan	5.0 (4.4, 5.8)	12.5 (9.9, 15.8)	-0.29 (-0.66, 0.09)	70.5 (61.7, 80.5)	84.3 (72.1, 98.8)	-3.22 (-3.75, -2.68)	2.3 (2.0, 2.6)	3.3 (2.8, 4.0)	-2.53 (-3.06, -1.98)
Jordan	2.9 (2.1, 4.0)	2.4 (1.7, 3.3)	0.11 (-0.11, 0.33)	73.6 (53.7, 100.0)	46.5 (32.6, 64.7)	-0.92 (-1.21, -0.63)	2.7 (1.9, 3.8)	1.8 (1.3, 2.6)	-0.65 (-0.09, -0.40)
Kazakhstan	1.6 (1.2, 2.2)	4.5 (3.2, 6.3)	-0.05 (-1.32, 1.24)	43.6 (32.3, 57.9)	118.6 (83.7, 165.6)	-0.16 (-1.52, 1.21)	1.4 (1.0, 2.0)	4.0 (2.8, 5.7)	0.09 (-1.21, 1.41)
Kenya	0.7 (0.4, 1.2)	0.6 (0.4, 0.9)	0.87 (0.37, 1.37)	18.5 (11.6, 33.3)	17.3 (11.0, 26.0)	1.29 (0.78, 1.80)	0.6 (0.4, 1.1)	0.6 (0.4, 0.9)	1.23 (0.71, 1.76)
Kiribati	29.5 (21.5, 39.4)	30.1 (21.7, 40.2)	0.99 (0.89, 1.10)	877.4 (638.0, 1161.5)	894.5 (646.3, 1205.0)	0.98 (0.89, 1.08)	26.6 (19.7, 35.7)	26.3 (19.0, 35.4)	0.86 (0.78, 0.95)
Kuwait	3.8 (2.9, 5.0)	5.0 (3.3, 7.0)	1.74 (1.57, 1.92)	88.4 (68.1, 114.0)	71.6 (47.5, 102.5)	0.30 (-0.05, 0.64)	3.2 (2.3, 4.2)	2.9 (1.9, 4.2)	0.68 (0.33, 1.03)
Kyrgyzstan	0.3 (0.2, 0.4)	0.7 (0.5, 1.0)	4.14 (3.72, 4.56)	8.5 (6.2, 11.3)	19.5 (13.8, 27.0)	4.38 (3.85, 4.92)	0.3 (0.2, 0.4)	0.7 (0.5, 1.0)	4.32 (3.83, 4.82)
Lao People's Democratic Republic	8.0 (5.0, 11.9)	5.8 (3.9, 8.1)	-0.17 (-0.28, -0.07)	231.4 (144.4, 345.2)	157.7 (106.0, 223.3)	-0.36 (-0.47, -0.25)	7.6 (4.8, 11.0)	5.3 (3.6, 7.5)	-0.26 (-0.37, -0.15)

Country	Age-standardized prevalence rate			Age-standardized DALY rate			Age-standardized mortality rate		
	1990 No. (95% UI)	2019 No. (95% UI)	EAPC % (95% CI)	1990 No. (95% UI)	2019 No. (95% UI)	EAPC % (95% CI)	1990 No. (95% UI)	2019 No. (95% UI)	EAPC % (95% CI)
Latvia	0.5 (0.4, 0.7)	2.0 (1.4, 2.9)	1.94 (0.97, 2.92)	13.3 (9.9, 18.0)	47.5 (32.7, 67.8)	1.46 (0.37, 2.56)	0.5 (0.3, 0.6)	1.6 (1.1, 2.3)	1.57 (0.53, 2.63)
Lebanon	4.0 (3.0, 5.3)	10.5 (7.4, 15.0)	3.50 (3.21, 3.80)	101.5 (77.0, 130.7)	138.9 (96.8, 195.8)	0.99 (0.83, 1.15)	3.8 (2.8, 4.8)	5.2 (3.6, 7.4)	1.03 (0.91, 1.16)
Lesotho	2.9 (1.5, 6.7)	9.6 (3.3, 16.6)	6.27 (5.42, 7.13)	81.9 (42.5, 192.9)	285.9 (95.2, 492.4)	6.49 (5.56, 7.44)	2.6 (1.4, 6.1)	9.0 (3.1, 15.2)	6.36 (5.39, 7.33)
Liberia	5.2 (3.8, 7.1)	2.8 (1.8, 4.4)	0.33 (0.10, 0.55)	143.6 (104.0, 192.4)	75.9 (49.3, 119.3)	0.23 (-0.04, 0.51)	5.2 (3.8, 6.9)	2.8 (1.8, 4.2)	0.27 (0.02, 0.51)
Libya	4.8 (3.3, 6.7)	8.2 (5.5, 12.0)	2.36 (2.25, 2.46)	126.0 (85.8, 173.9)	186.1 (122.9, 276.7)	1.76 (1.68, 1.83)	4.3 (3.0, 6.0)	6.4 (4.2, 9.3)	1.74 (1.67, 1.81)
Lithuania	0.5 (0.4, 0.7)	2.2 (1.5, 3.2)	2.12 (1.70, 2.55)	12.4 (9.3, 16.3)	42.4 (28.2, 61.7)	1.50 (0.94, 2.05)	0.4 (0.3, 0.6)	1.4 (0.9, 2.1)	1.56 (1.04, 2.08)
Luxembourg	0.4 (0.3, 0.6)	4.1 (2.6, 6.0)	6.87 (6.40, 7.34)	8.9 (6.3, 12.4)	48.9 (32.2, 72.4)	4.75 (4.45, 5.04)	0.3 (0.2, 0.5)	1.8 (1.1, 2.7)	4.97 (4.67, 5.27)
Madagascar	1.1 (0.7, 1.9)	0.6 (0.3, 1.0)	-0.20 (-0.30, -0.09)	31.2 (20.0, 55.3)	16.2 (9.6, 26.9)	-0.20 (-0.30, -0.09)	1.0 (0.6, 1.8)	0.5 (0.3, 0.9)	-0.14 (-0.27, 0.00)
Malawi	1.4 (0.9, 2.2)	0.7 (0.5, 1.0)	-1.09 (-1.54, -0.65)	39.8 (26.4, 62.8)	19.0 (12.6, 27.2)	-1.29 (-1.85, -0.74)	1.3 (0.8, 2.1)	0.7 (0.4, 1.0)	-1.00 (-1.52, -0.47)
Malaysia	7.0 (5.5, 8.5)	10.4 (7.4, 13.8)	1.90 (1.63, 2.18)	185.0 (147.0, 224.9)	244.2 (174.4, 324.4)	1.56 (1.27, 1.86)	6.7 (5.3, 8.3)	9.1 (6.5, 12.0)	1.58 (1.25, 1.92)
Maldives	4.7 (2.9, 7.7)	4.3 (3.1, 5.8)	0.66 (0.29, 1.03)	125.6 (77.2, 205.8)	93.2 (66.4, 124.2)	-0.16 (-0.49, 0.17)	4.7 (2.9, 7.5)	3.5 (2.5, 4.8)	-0.10 (-0.37, 0.17)

Country	Age-standardized prevalence rate			Age-standardized DALY rate			Age-standardized mortality rate		
	1990<br>No. (95% UI)	2019<br>No. (95% UI)	EAPC<br>% (95% CI)	1990<br>No. (95% UI)	2019<br>No. (95% UI)	EAPC<br>% (95% CI)	1990<br>No. (95% UI)	2019<br>No. (95% UI)	EAPC<br>% (95% CI)
Mali	6.9 (4.9, 9.0)	4.5 (2.8, 6.5)	1.08 (0.98, 1.18)	196.7 (141.9, 257.1)	125.0 (80.3, 183.5)	0.99 (0.88, 1.11)	6.3 (4.5, 8.5)	4.1 (2.6, 5.9)	1.08 (0.97, 1.18)
Malta	0.6 (0.4, 0.8)	2.9 (2.1, 4.2)	3.39 (3.06, 3.72)	12.5 (8.9, 17.6)	39.9 (28.0, 56.2)	1.79 (1.48, 2.11)	0.5 (0.3, 0.7)	1.4 (1.0, 2.1)	1.74 (1.41, 2.06)
Marshall Islands	34.0 (24.2, 47.9)	40.4 (27.0, 58.4)	1.41 (1.23, 1.59)	958.3 (688.9, 1330.2)	1140.7 (768.2, 1631.8)	1.34 (1.17, 1.51)	32.2 (22.5, 45.6)	37.1 (24.7, 54.4)	1.26 (1.09, 1.43)
Mauritania	3.2 (2.4, 4.1)	1.4 (0.9, 2.0)	-1.18 (-1.36, -0.99)	88.1 (65.8, 113.0)	37.2 (24.4, 52.0)	-1.36 (-1.55, -1.17)	3.1 (2.3, 4.0)	1.4 (1.0, 2.0)	-1.14 (-1.32, -0.97)
Mauritius	2.0 (1.5, 2.5)	5.0 (3.3, 7.0)	4.04 (3.42, 4.67)	51.7 (40.5, 64.7)	113.0 (76.3, 158.4)	3.70 (3.07, 4.34)	1.8 (1.4, 2.3)	4.3 (2.8, 6.1)	3.79 (3.20, 4.39)
Mexico	1.2 (1.0, 1.4)	2.0 (1.5, 2.5)	1.81 (1.62, 2.00)	31.3 (26.5, 37.0)	47.8 (38.0, 59.3)	1.55 (1.37, 1.73)	1.1 (0.9, 1.3)	1.8 (1.4, 2.2)	1.64 (1.45, 1.83)
Micronesia (Federated States of)	16.7 (11.4, 23.1)	27.1 (16.7, 40.7)	2.37 (2.08, 2.67)	471.8 (320.1, 653.9)	750.9 (448.7, 1156.3)	2.29 (1.98, 2.60)	15.5 (10.5, 21.5)	24.3 (15.1, 35.4)	2.23 (1.95, 2.51)
Monaco	0.8 (0.5, 1.2)	9.7 (6.4, 13.8)	6.85 (5.96, 7.75)	13.7 (9.1, 20.1)	110.7 (71.1, 160.8)	5.35 (4.56, 6.14)	0.5 (0.3, 0.7)	3.9 (2.5, 5.8)	5.48 (4.67, 6.29)
Mongolia	15.1 (10.5, 20.9)	24.9 (16.5, 35.8)	3.02 (2.60, 3.44)	415.7 (291.5, 575.8)	666.0 (443.5, 965.0)	2.87 (2.44, 3.30)	14.2 (10.0, 19.8)	24.6 (16.5, 35.6)	3.26 (2.80, 3.72)
Montenegro	3.2 (2.2, 4.4)	6.9 (4.7, 9.9)	1.28 (0.87, 1.68)	82.4 (58.2, 112.9)	164.5 (112.8, 234.4)	0.95 (0.52, 1.38)	2.8 (1.9, 3.9)	5.7 (3.8, 8.2)	1.11 (0.68, 1.55)
Morocco	1.2 (0.8, 1.6)	2.3 (1.6, 3.3)	2.46 (2.28, 2.63)	29.7 (21.1, 41.1)	54.8 (36.9, 78.0)	2.23 (2.07, 2.40)	1.2 (0.8, 1.6)	2.2 (1.5, 3.2)	2.29 (2.08, 2.50)

Country	Age-standardized prevalence rate			Age-standardized DALY rate			Age-standardized mortality rate		
	1990	2019	EAPC	1990	2019	EAPC	1990	2019	EAPC
	No. (95% UI)	No. (95% UI)	% (95% CI)	No. (95% UI)	No. (95% UI)	% (95% CI)	No. (95% UI)	No. (95% UI)	% (95% CI)
Mozambique	1.0 (0.6, 1.7)	1.2 (0.8, 1.8)	3.50 (3.25, 3.76)	26.9 (17.0, 47.8)	34.1 (21.3, 48.7)	3.59 (3.34, 3.84)	1.0 (0.6, 1.7)	1.2 (0.8, 1.7)	3.43 (3.16, 3.70)
Myanmar	1.9 (1.2, 2.8)	3.6 (2.5, 4.9)	2.53 (2.39, 2.67)	51.3 (33.3, 78.5)	93.9 (67.1, 128.7)	2.39 (2.17, 2.61)	1.9 (1.2, 2.9)	3.4 (2.4, 4.7)	2.34 (2.15, 2.53)
Namibia	1.1 (0.6, 2.3)	1.6 (1.1, 2.3)	3.00 (2.41, 3.60)	31.5 (16.0, 65.8)	45.4 (29.4, 64.7)	2.98 (2.33, 3.63)	1.1 (0.6, 2.2)	1.5 (1.0, 2.1)	2.87 (2.22, 3.53)
Nauru	17.0 (11.4, 23.2)	15.9 (10.4, 23.5)	1.16 (0.86, 1.45)	478.6 (318.9, 661.9)	440.0 (283.6, 659.2)	1.14 (0.84, 1.45)	15.6 (10.6, 21.1)	14.2 (9.6, 20.3)	1.11 (0.75, 1.47)
Nepal	0.7 (0.5, 1.0)	1.0 (0.6, 1.6)	1.87 (1.70, 2.04)	19.3 (12.9, 27.9)	27.1 (17.0, 42.5)	1.71 (1.54, 1.88)	0.7 (0.4, 1.0)	1.0 (0.6, 1.6)	1.97 (1.80, 2.14)
Netherlands	0.4 (0.3, 0.5)	2.6 (1.7, 3.8)	5.55 (5.29, 5.80)	6.4 (4.7, 8.9)	26.5 (19.3, 36.1)	3.30 (3.17, 3.44)	0.2 (0.2, 0.3)	0.9 (0.7, 1.3)	3.42 (3.28, 3.56)
New Zealand	0.6 (0.5, 0.7)	4.0 (3.0, 5.3)	4.96 (4.67, 5.25)	10.3 (8.7, 12.3)	42.0 (35.3, 50.2)	3.11 (2.71, 3.50)	0.3 (0.3, 0.4)	1.4 (1.1, 1.6)	3.28 (2.89, 3.66)
Nicaragua	1.5 (1.1, 2.1)	1.8 (1.2, 2.6)	1.46 (0.90, 2.02)	41.0 (29.5, 56.2)	42.9 (28.4, 62.3)	1.18 (0.59, 1.77)	1.4 (0.9, 2.0)	1.5 (1.0, 2.3)	1.40 (0.93, 1.87)
Niger	0.2 (0.1, 0.3)	0.1 (0.1, 0.2)	2.08 (1.77, 2.38)	5.6 (4.1, 7.5)	4.0 (2.6, 5.5)	2.01 (1.70, 2.32)	0.2 (0.1, 0.3)	0.1 (0.1, 0.2)	2.07 (1.79, 2.35)
Nigeria	1.3 (1.0, 1.8)	0.7 (0.5, 0.9)	0.39 (0.26, 0.51)	36.1 (26.6, 47.3)	18.2 (13.4, 24.0)	0.21 (0.06, 0.37)	1.3 (1.0, 1.7)	0.7 (0.6, 1.0)	0.56 (0.47, 0.65)
Niue	19.0 (13.8, 25.1)	37.6 (26.1, 51.8)	1.68 (1.60, 1.75)	522.3 (379.0, 690.1)	942.7 (648.5, 1322.8)	1.32 (1.24, 1.40)	16.9 (12.4, 22.2)	31.0 (22.1, 42.4)	1.38 (1.31, 1.46)

Country	Age-standardized prevalence rate			Age-standardized DALY rate			Age-standardized mortality rate		
	1990<br>No. (95% UI)	2019<br>No. (95% UI)	EAPC<br>% (95% CI)	1990<br>No. (95% UI)	2019<br>No. (95% UI)	EAPC<br>% (95% CI)	1990<br>No. (95% UI)	2019<br>No. (95% UI)	EAPC<br>% (95% CI)
North Macedonia	4.7 (3.4, 6.4)	10.7 (7.2, 16.0)	1.47 (1.14, 1.80)	125.0 (90.6, 167.1)	261.7 (175.4, 384.0)	1.15 (0.79, 1.50)	4.4 (3.1, 6.0)	9.6 (6.4, 14.2)	1.36 (0.99, 1.73)
Northern Mariana Islands	11.4 (8.2, 15.1)	33.0 (24.8, 43.3)	3.16 (3.02, 3.30)	297.3 (211.7, 397.1)	769.7 (576.4, 1013.2)	2.84 (2.68, 3.01)	9.8 (7.0, 13.0)	25.9 (19.5, 33.6)	2.93 (2.76, 3.10)
Norway	0.7 (0.6, 0.8)	3.1 (2.3, 3.9)	3.58 (3.32, 3.84)	12.3 (10.5, 14.4)	34.0 (27.8, 41.9)	1.76 (1.63, 1.89)	0.4 (0.3, 0.5)	1.2 (0.9, 1.4)	1.91 (1.76, 2.05)
Oman	3.6 (2.3, 5.3)	4.7 (3.3, 6.5)	3.25 (3.02, 3.47)	93.2 (58.2, 137.7)	87.8 (60.8, 123.4)	2.11 (1.84, 2.39)	3.2 (2.0, 4.7)	3.2 (2.2, 4.4)	2.29 (2.02, 2.56)
Pakistan	1.0 (0.7, 1.4)	1.0 (0.8, 1.3)	1.89 (1.65, 2.13)	29.2 (19.9, 40.6)	29.6 (22.9, 38.3)	1.88 (1.63, 2.13)	0.9 (0.6, 1.3)	0.9 (0.7, 1.2)	1.84 (1.59, 2.08)
Palau	21.0 (13.6, 30.0)	52.4 (37.8, 71.0)	2.28 (2.11, 2.46)	588.6 (383.3, 842.8)	1393.7 (1001.4, 1893.6)	2.16 (2.00, 2.32)	18.7 (12.1, 26.6)	42.6 (30.9, 57.2)	2.00 (1.86, 2.14)
Palestine	6.3 (4.2, 9.3)	5.2 (3.8, 7.1)	1.29 (1.06, 1.52)	158.4 (106.4, 230.8)	113.5 (83.4, 151.0)	0.84 (0.66, 1.03)	6.0 (4.0, 8.7)	4.3 (3.1, 5.9)	0.82 (0.65, 0.98)
Panama	1.5 (1.0, 2.0)	1.9 (1.2, 2.9)	1.06 (0.65, 1.47)	38.7 (28.3, 52.2)	46.2 (29.8, 68.8)	0.73 (0.34, 1.13)	1.3 (0.9, 1.8)	1.6 (1.0, 2.3)	0.84 (0.47, 1.22)
Papua New Guinea	1.9 (1.3, 2.6)	2.2 (1.5, 3.0)	1.89 (1.83, 1.95)	49.6 (35.2, 69.0)	55.9 (38.8, 78.0)	1.81 (1.75, 1.88)	2.0 (1.4, 2.7)	2.3 (1.6, 3.2)	1.91 (1.85, 1.97)
Paraguay	0.6 (0.4, 0.9)	0.8 (0.5, 1.2)	1.31 (0.77, 1.85)	16.8 (11.9, 23.3)	19.6 (12.3, 29.6)	1.14 (0.54, 1.74)	0.6 (0.4, 0.8)	0.7 (0.4, 1.1)	1.41 (0.85, 1.97)
Peru	2.9 (2.2, 3.7)	1.9 (1.3, 2.7)	-2.27 (-2.93, -1.61)	75.6 (59.1, 95.0)	46.3 (31.3, 64.7)	-2.55 (-3.24, -1.85)	2.8 (2.1, 3.6)	1.7 (1.2, 2.5)	-2.43 (-3.12, -1.74)

Country	Age-standardized prevalence rate			Age-standardized DALY rate			Age-standardized mortality rate		
	1990 No. (95% UI)	2019 No. (95% UI)	EAPC % (95% CI)	1990 No. (95% UI)	2019 No. (95% UI)	EAPC % (95% CI)	1990 No. (95% UI)	2019 No. (95% UI)	EAPC % (95% CI)
Philippines	8.3 (6.1, 10.5)	4.7 (3.6, 6.1)	-1.77 (-2.04, -1.49)	237.7 (174.7, 299.4)	131.6 (103.4, 165.6)	-1.90 (-2.19, -1.60)	7.4 (5.4, 9.5)	4.1 (3.2, 5.2)	-1.85 (-2.15, -1.56)
Poland	3.2 (2.8, 3.7)	1.8 (1.4, 2.3)	-3.43 (-4.96, -1.88)	84.5 (73.1, 97.5)	43.1 (33.8, 55.1)	-3.78 (-5.32, -2.21)	3.1 (2.7, 3.7)	1.6 (1.3, 2.1)	-3.72 (-5.26, -2.15)
Portugal	0.6 (0.4, 0.9)	6.1 (3.9, 9.4)	5.66 (5.10, 6.22)	15.5 (11.2, 21.2)	103.2 (71.4, 147.6)	4.22 (3.66, 4.79)	0.5 (0.4, 0.7)	3.4 (2.4, 5.0)	4.33 (3.77, 4.89)
Puerto Rico	5.8 (4.3, 7.6)	6.4 (4.1, 9.4)	-0.16 (-0.67, 0.36)	143.1 (107.9, 187.5)	132.0 (85.3, 194.5)	-0.95 (-1.56, -0.34)	5.3 (3.8, 7.1)	4.8 (3.0, 7.0)	-0.99 (-1.63, -0.35)
Qatar	21.5 (14.3, 31.9)	28.2 (17.6, 43.7)	3.15 (2.91, 3.40)	516.8 (346.2, 762.6)	408.1 (254.8, 636.9)	0.79 (0.48, 1.10)	21.4 (13.9, 32.3)	19.1 (11.9, 29.5)	1.32 (1.03, 1.61)
Republic of Korea	12.4 (9.9, 15.4)	94.1 (73.3, 119.7)	6.59 (5.04, 8.17)	311.3 (248.6, 388.3)	816.3 (675.3, 961.2)	2.27 (0.72, 3.84)	10.5 (8.3, 12.9)	30.2 (24.8, 35.9)	2.70 (1.20, 4.22)
Republic of Moldova	0.5 (0.4, 0.7)	1.4 (1.0, 2.0)	-0.40 (-2.36, 1.60)	14.3 (10.6, 19.1)	35.1 (24.8, 49.1)	-0.88 (-2.90, 1.18)	0.5 (0.3, 0.7)	1.2 (0.8, 1.8)	-0.43 (-2.36, 1.53)
Romania	0.6 (0.4, 0.8)	2.3 (1.6, 3.4)	3.67 (3.15, 4.18)	15.6 (11.5, 20.7)	57.9 (39.5, 83.1)	3.34 (2.83, 3.84)	0.5 (0.4, 0.7)	1.9 (1.3, 2.8)	3.27 (2.78, 3.77)
Russian Federation	0.5 (0.4, 0.6)	1.6 (1.2, 2.0)	2.88 (2.61, 3.14)	13.8 (11.8, 16.0)	41.0 (31.9, 53.1)	2.72 (2.39, 3.05)	0.4 (0.4, 0.5)	1.3 (1.0, 1.7)	2.83 (2.52, 3.14)
Rwanda	1.7 (1.1, 2.6)	0.9 (0.6, 1.4)	-0.88 (-1.18, -0.59)	49.4 (32.0, 75.3)	26.4 (17.0, 38.4)	-1.06 (-1.42, -0.70)	1.6 (1.1, 2.4)	0.9 (0.6, 1.2)	-1.04 (-1.41, -0.67)
Saint Kitts and Nevis	12.1 (9.1, 15.5)	5.1 (3.4, 7.2)	-4.34 (-5.43, -3.24)	325.1 (249.6, 418.1)	125.0 (82.7, 175.9)	-4.60 (-5.72, -3.47)	11.1 (8.2, 14.5)	4.6 (3.1, 6.4)	-4.34 (-5.42, -3.24)

Country	Age-standardized prevalence rate			Age-standardized DALY rate			Age-standardized mortality rate		
	1990<br>No. (95% UI)	2019<br>No. (95% UI)	EAPC<br>% (95% CI)	1990<br>No. (95% UI)	2019<br>No. (95% UI)	EAPC<br>% (95% CI)	1990<br>No. (95% UI)	2019<br>No. (95% UI)	EAPC<br>% (95% CI)
Saint Lucia	7.0 (5.3, 9.2)	3.8 (2.7, 5.2)	-3.01 (-4.01, -2.00)	189.5 (145.1, 246.3)	95.5 (67.8, 130.4)	-3.23 (-4.27, -2.18)	6.5 (4.8, 8.6)	3.4 (2.4, 4.7)	-3.10 (-4.14, -2.06)
Saint Vincent and the Grenadines	7.6 (5.7, 10.0)	5.0 (3.6, 6.6)	-2.29 (-3.05, -1.52)	206.3 (158.5, 267.1)	130.6 (94.8, 174.5)	-2.23 (-2.97, -1.49)	7.0 (5.2, 9.4)	4.4 (3.2, 6.0)	-2.25 (-3.01, -1.47)
Samoa	11.0 (7.9, 14.6)	12.1 (8.7, 16.6)	1.16 (1.07, 1.24)	299.9 (215.9, 402.6)	324.5 (228.4, 447.2)	1.07 (0.98, 1.15)	10.0 (7.2, 13.1)	10.6 (7.7, 14.3)	1.00 (0.94, 1.06)
San Marino	0.5 (0.4, 0.7)	3.0 (1.9, 4.4)	5.06 (4.84, 5.28)	8.8 (6.0, 12.9)	33.7 (19.0, 56.6)	3.35 (3.08, 3.61)	0.3 (0.2, 0.5)	1.2 (0.7, 2.0)	3.18 (2.93, 3.43)
Sao Tome and Principe	1.7 (1.2, 2.2)	1.5 (1.0, 2.2)	1.55 (1.41, 1.68)	47.9 (32.9, 62.3)	42.4 (26.4, 61.1)	1.41 (1.25, 1.58)	1.6 (1.2, 2.1)	1.4 (0.9, 2.0)	1.41 (1.28, 1.55)
Saudi Arabia	5.3 (3.5, 7.5)	7.2 (4.9, 10.2)	1.72 (1.57, 1.87)	151.3 (97.6, 214.9)	126.2 (84.5, 183.8)	-0.16 (-0.40, 0.08)	5.6 (3.6, 8.0)	5.0 (3.3, 7.2)	0.13 (-0.11, 0.38)
Senegal	2.3 (1.8, 2.9)	1.7 (1.2, 2.3)	1.38 (1.13, 1.63)	61.9 (47.7, 77.0)	45.0 (31.5, 60.7)	1.27 (0.99, 1.56)	2.3 (1.8, 2.9)	1.7 (1.2, 2.2)	1.21 (0.94, 1.48)
Serbia	3.0 (2.1, 4.1)	6.6 (4.3, 9.9)	0.71 (0.32, 1.10)	77.5 (55.9, 104.2)	138.2 (89.5, 210.2)	-0.06 (-0.50, 0.38)	2.7 (1.9, 3.8)	5.1 (3.3, 7.6)	0.17 (-0.28, 0.62)
Seychelles	6.8 (5.1, 9.0)	11.6 (8.5, 15.5)	1.52 (1.37, 1.66)	186.9 (142.4, 243.2)	290.1 (213.8, 387.0)	1.11 (0.94, 1.27)	6.3 (4.7, 8.2)	9.9 (7.2, 13.2)	1.17 (1.02, 1.32)
Sierra Leone	1.8 (1.2, 2.4)	1.3 (0.9, 1.8)	1.44 (1.34, 1.54)	48.7 (32.7, 66.3)	34.9 (24.0, 50.0)	1.41 (1.31, 1.51)	1.8 (1.2, 2.4)	1.2 (0.9, 1.8)	1.36 (1.27, 1.45)
Singapore	15.7 (13.3, 18.3)	44.5 (32.3, 58.9)	2.45 (2.06, 2.85)	320.9 (275.6, 367.7)	378.8 (304.6, 456.5)	-0.85 (-1.29, -0.41)	12.3 (10.2, 14.4)	16.6 (12.9, 20.3)	-0.28 (-0.71, 0.15)

Country	Age-standardized prevalence rate			Age-standardized DALY rate			Age-standardized mortality rate		
	1990 No. (95% UI)	2019 No. (95% UI)	EAPC % (95% CI)	1990 No. (95% UI)	2019 No. (95% UI)	EAPC % (95% CI)	1990 No. (95% UI)	2019 No. (95% UI)	EAPC % (95% CI)
Slovakia	1.9 (1.4, 2.5)	2.9 (1.9, 4.2)	-0.77 (-1.14, -0.40)	48.4 (36.5, 64.0)	62.2 (40.5, 91.1)	-1.44 (-1.84, -1.04)	1.6 (1.2, 2.2)	2.2 (1.4, 3.3)	-1.28 (-1.7, -0.86)
Slovenia	1.3 (0.9, 2.0)	5.7 (3.6, 8.6)	3.01 (2.46, 3.56)	33.6 (22.3, 50.2)	112.5 (70.8, 166.8)	1.93 (1.30, 2.56)	1.2 (0.8, 1.7)	4.2 (2.7, 6.4)	2.25 (1.63, 2.87)
Solomon Islands	10.1 (7.1, 13.6)	9.7 (7.3, 12.7)	1.44 (1.34, 1.54)	290.4 (205.9, 385.6)	271.7 (203.4, 356.8)	1.37 (1.25, 1.48)	9.1 (6.3, 12.3)	8.6 (6.5, 11.2)	1.40 (1.29, 1.51)
Somalia	1.5 (0.8, 2.8)	0.8 (0.5, 1.5)	0.80 (0.70, 0.90)	42.9 (23.6, 82.3)	23.3 (13.4, 44.6)	0.80 (0.69, 0.92)	1.4 (0.8, 2.8)	0.8 (0.5, 1.5)	0.83 (0.71, 0.94)
South Africa	3.9 (2.5, 6.9)	4.9 (4.1, 5.9)	1.00 (0.26, 1.75)	113.5 (74.1, 199.7)	141.0 (118.4, 166.3)	0.82 (0.02, 1.63)	3.4 (2.1, 6.0)	4.3 (3.6, 5.1)	0.97 (0.17, 1.77)
South Sudan	1.1 (0.6, 2.3)	0.7 (0.4, 1.3)	0.65 (0.58, 0.72)	31.6 (17.3, 63.8)	18.9 (9.8, 35.9)	0.61 (0.52, 0.71)	1.1 (0.6, 2.2)	0.7 (0.3, 1.3)	0.75 (0.66, 0.83)
Spain	1.1 (0.8, 1.5)	6.4 (4.1, 9.5)	3.84 (2.96, 4.72)	24.0 (17.5, 32.4)	70.4 (48.6, 100.2)	1.26 (0.74, 1.79)	0.8 (0.6, 1.2)	2.5 (1.7, 3.6)	1.28 (0.75, 1.81)
Sri Lanka	1.8 (1.4, 2.3)	6.0 (3.9, 8.7)	4.61 (4.11, 5.10)	47.7 (36.7, 60.6)	131.3 (86.0, 190.6)	3.99 (3.45, 4.54)	1.8 (1.3, 2.3)	4.8 (3.1, 7.1)	4.09 (3.53, 4.66)
Sudan	2.3 (1.4, 3.6)	2.4 (1.3, 4.0)	2.31 (2.20, 2.42)	60.9 (35.7, 95.0)	60.0 (32.3, 97.5)	2.05 (1.95, 2.16)	2.3 (1.3, 3.6)	2.3 (1.3, 3.8)	2.14 (2.04, 2.24)
Suriname	5.9 (4.4, 7.7)	4.0 (2.7, 5.6)	-1.22 (-1.96, -0.47)	160.8 (121.5, 210.0)	105.6 (73.1, 148.5)	-1.56 (-2.38, -0.73)	5.4 (3.9, 7.2)	3.6 (2.5, 5.1)	-1.45 (-2.23, -0.66)
Sweden	0.2 (0.2, 0.3)	0.9 (0.7, 1.2)	3.59 (2.97, 4.22)	6.0 (4.7, 7.5)	17.5 (13.7, 22.6)	2.79 (2.17, 3.41)	0.2 (0.2, 0.3)	0.6 (0.5, 0.8)	2.84 (2.24, 3.44)

Country	Age-standardized prevalence rate			Age-standardized DALY rate			Age-standardized mortality rate		
	1990 No. (95% UI)	2019 No. (95% UI)	EAPC % (95% CI)	1990 No. (95% UI)	2019 No. (95% UI)	EAPC % (95% CI)	1990 No. (95% UI)	2019 No. (95% UI)	EAPC % (95% CI)
Switzerland	0.8 (0.6, 1.2)	6.0 (3.7, 9.2)	4.10 (3.28, 4.92)	12.6 (8.9, 17.7)	54.0 (36.8, 76.3)	2.55 (1.95, 3.15)	0.4 (0.3, 0.6)	2.0 (1.3, 2.9)	2.81 (2.23, 3.40)
Syrian Arab Republic	4.0 (2.8, 5.6)	5.3 (3.6, 7.8)	1.02 (0.85, 1.20)	104.5 (74.7, 144.9)	110.6 (74.5, 159.6)	0.06 (-0.19, 0.31)	3.7 (2.5, 5.2)	4.1 (2.7, 5.9)	0.18 (-0.06, 0.41)
Taiwan (Province of China)	14.1 (12.0, 16.1)	23.2 (16.7, 31.8)	-1.22 (-2.64, 0.23)	357.7 (309.0, 405.3)	320.5 (233.7, 426.3)	-3.35 (-4.18, -2.52)	11.1 (9.4, 12.9)	11.1 (8.0, 15.1)	-2.89 (-3.71, -2.07)
Tajikistan	0.2 (0.2, 0.3)	1.2 (0.8, 1.7)	7.76 (7.38, 8.15)	6.2 (4.4, 8.4)	32.8 (22.8, 45.6)	8.11 (7.60, 8.61)	0.2 (0.1, 0.3)	1.1 (0.7, 1.6)	8.05 (7.72, 8.39)
Thailand	15.6 (12.4, 19.2)	38.4 (26.4, 54.5)	1.10 (0.82, 1.37)	429.6 (338.6, 529.3)	889.3 (610.3, 1258.0)	0.45 (0.17, 0.73)	14.2 (11.1, 17.7)	29.9 (20.4, 42.3)	0.59 (0.32, 0.85)
Timor-Leste	4.1 (2.6, 6.4)	4.1 (2.2, 6.2)	0.98 (0.64, 1.31)	114.9 (71.6, 179.3)	108.6 (57.6, 168.3)	0.85 (0.49, 1.21)	3.9 (2.4, 6.0)	3.8 (2.1, 5.9)	1.06 (0.75, 1.37)
Togo	2.7 (2.0, 3.6)	1.6 (1.2, 2.3)	-0.09 (-0.34, 0.16)	75.1 (55.3, 101.2)	45.1 (31.7, 62.8)	-0.08 (-0.37, 0.21)	2.7 (1.9, 3.6)	1.6 (1.1, 2.2)	-0.14 (-0.44, 0.16)
Tokelau	13.3 (8.9, 18.7)	20.5 (13.2, 29.4)	1.63 (1.58, 1.68)	369.6 (249.4, 522.1)	541.2 (346.7, 778.8)	1.46 (1.41, 1.51)	12.3 (8.2, 17.2)	17.9 (11.6, 25.5)	1.43 (1.38, 1.49)
Tonga	43.2 (28.1, 60.0)	57.5 (39.0, 81.3)	1.67 (1.55, 1.79)	1183.9 (774.2, 1628.4)	1550.8 (1044.5, 2192.5)	1.64 (1.49, 1.79)	39.2 (25.7, 54.4)	50.4 (34.1, 70.6)	1.53 (1.39, 1.67)
Trinidad and Tobago	8.1 (6.1, 10.6)	4.6 (3.0, 6.9)	-3.19 (-4.19, -2.17)	217.6 (164.6, 283.9)	118.5 (77.2, 173.6)	-3.44 (-4.47, -2.40)	7.7 (5.6, 10.2)	4.2 (2.7, 6.2)	-3.42 (-4.43, -2.41)
Tunisia	1.3 (0.9, 1.8)	2.9 (1.8, 4.5)	2.60 (2.52, 2.67)	32.8 (22.5, 46.4)	55.4 (33.1, 85.7)	1.50 (1.45, 1.55)	1.2 (0.8, 1.7)	2.0 (1.2, 3.1)	1.53 (1.47, 1.59)

Country	Age-standardized prevalence rate			Age-standardized DALY rate			Age-standardized mortality rate		
	1990 No. (95% UI)	2019 No. (95% UI)	EAPC % (95% CI)	1990 No. (95% UI)	2019 No. (95% UI)	EAPC % (95% CI)	1990 No. (95% UI)	2019 No. (95% UI)	EAPC % (95% CI)
Turkey	3.2 (2.4, 4.1)	4.5 (3.3, 6.1)	2.29 (1.57, 3.01)	83.2 (63.1, 106.4)	81.7 (60.1, 109.8)	0.91 (0.32, 1.51)	3.0 (2.3, 3.9)	3.2 (2.3, 4.3)	1.21 (0.60, 1.81)
Turkmenistan	0.4 (0.3, 0.5)	3.3 (2.3, 4.7)	9.58 (8.10, 11.09)	8.8 (6.6, 11.7)	91.8 (63.2, 130.7)	9.99 (8.43, 11.57)	0.3 (0.2, 0.4)	2.7 (1.9, 3.9)	9.16 (7.74, 10.59)
Tuvalu	17.3 (12.1, 23.8)	24.0 (16.6, 34.4)	1.45 (1.39, 1.50)	488.3 (344.1, 672.7)	669.9 (457.0, 964.4)	1.43 (1.37, 1.49)	16.1 (11.3, 22.4)	21.5 (15.0, 30.8)	1.28 (1.21, 1.35)
Uganda	1.9 (1.3, 2.7)	1.5 (1.0, 2.2)	2.08 (1.74, 2.42)	55.1 (37.8, 78.3)	42.4 (28.4, 61.6)	2.02 (1.67, 2.36)	1.8 (1.2, 2.6)	1.3 (0.9, 1.9)	1.90 (1.58, 2.23)
Ukraine	0.3 (0.3, 0.3)	1.9 (1.5, 2.3)	5.94 (5.10, 6.78)	7.2 (6.2, 8.4)	45.7 (35.8, 57.1)	6.01 (5.04, 6.99)	0.3 (0.2, 0.3)	1.5 (1.1, 1.8)	5.33 (4.46, 6.21)
United Arab Emirates	5.3 (2.5, 10.8)	6.9 (2.9, 16.4)	1.94 (1.62, 2.26)	137.2 (65.4, 282.5)	171.4 (69.7, 408.0)	1.83 (1.56, 2.11)	5.0 (2.3, 10.4)	6.2 (2.4, 15.1)	1.79 (1.45, 2.14)
United Kingdom	0.8 (0.7, 0.9)	8.4 (6.5, 10.7)	8.49 (8.03, 8.94)	12.6 (10.6, 14.9)	79.9 (67.0, 94.5)	6.30 (5.93, 6.68)	0.4 (0.4, 0.5)	2.9 (2.4, 3.5)	6.51 (6.13, 6.89)
United Republic of Tanzania	0.5 (0.3, 0.7)	0.4 (0.3, 0.6)	2.40 (2.17, 2.62)	12.9 (9.1, 18.0)	11.6 (7.8, 16.8)	2.27 (2.03, 2.51)	0.4 (0.3, 0.6)	0.4 (0.3, 0.6)	2.35 (2.10, 2.61)
United States of America	1.0 (0.9, 1.2)	6.3 (4.8, 8.2)	6.08 (5.57, 6.60)	18.1 (15.8, 20.7)	76.4 (62.2, 93.1)	4.82 (4.37, 5.27)	0.6 (0.5, 0.7)	2.7 (2.2, 3.3)	5.03 (4.56, 5.51)
United States Virgin Islands	2.9 (2.0, 4.0)	4.4 (3.0, 6.2)	-0.36 (-0.99, 0.28)	78.2 (54.1, 105.0)	108.9 (75.8, 155.5)	-0.59 (-1.23, 0.06)	2.7 (1.8, 3.7)	3.9 (2.7, 5.4)	-0.32 (-0.99, 0.35)
Uruguay	0.1 (0.1, 0.2)	1.1 (0.8, 1.7)	6.33 (5.93, 6.73)	3.7 (2.7, 5.2)	25.8 (18.5, 35.8)	6.01 (5.58, 6.44)	0.1 (0.1, 0.2)	0.9 (0.6, 1.3)	6.03 (5.61, 6.45)

Country	Age-standardized prevalence rate			Age-standardized DALY rate			Age-standardized mortality rate		
	1990<br>No. (95% UI)	2019<br>No. (95% UI)	EAPC<br>% (95% CI)	1990<br>No. (95% UI)	2019<br>No. (95% UI)	EAPC<br>% (95% CI)	1990<br>No. (95% UI)	2019<br>No. (95% UI)	EAPC<br>% (95% CI)
Uzbekistan	0.2 (0.2, 0.3)	3.2 (2.3, 4.5)	13.09 (11.47, 14.73)	6.0 (4.5, 7.9)	87.0 (62.0, 120.6)	13.52 (11.90, 15.16)	0.2 (0.2, 0.3)	2.9 (2.0, 4.1)	13.08 (11.53, 14.65)
Vanuatu	14.4 (8.8, 22.1)	17.7 (11.3, 26.5)	2.00 (1.90, 2.09)	402.1 (251.1, 621.6)	498.2 (315.0, 746.7)	1.98 (1.84, 2.12)	13.7 (8.3, 21.4)	16.3 (10.6, 24.2)	1.87 (1.74, 2.01)
Venezuela (Bolivarian Republic)	2.6 (1.8, 3.7)	1.4 (0.9, 2.1)	-1.67 (-2.90, -0.43)	69.1 (49.1, 94.7)	33.4 (21.3, 51.4)	-2.09 (-3.44, -0.72)	2.4 (1.6, 3.4)	1.2 (0.8, 1.9)	-1.87 (-3.21, -0.51)
Viet Nam	2.2 (1.5, 3.1)	2.3 (1.6, 3.3)	-0.19 (-0.40, 0.03)	59.5 (40.3, 81.5)	54.5 (37.1, 76.2)	-0.61 (-0.85, -0.36)	2.2 (1.5, 3.0)	2.1 (1.4, 3.0)	-0.4 (-0.63, -0.18)
Yemen	1.4 (0.8, 2.3)	1.1 (0.7, 1.7)	1.59 (1.47, 1.71)	37.2 (21.8, 60.7)	29.0 (18.2, 44.0)	1.41 (1.29, 1.53)	1.4 (0.8, 2.2)	1.1 (0.7, 1.7)	1.52 (1.41, 1.63)
Zambia	0.9 (0.6, 1.4)	0.6 (0.4, 0.9)	0.67 (0.19, 1.14)	25.0 (16.5, 38.2)	17.2 (11.6, 24.4)	0.56 (0.03, 1.09)	0.9 (0.6, 1.4)	0.6 (0.4, 0.8)	0.52 (-0.02, 1.06)
Zimbabwe	8.2 (5.5, 13.9)	7.4 (4.8, 11.3)	0.47 (-0.13, 1.07)	227.9 (152.1, 392.1)	211.5 (140.0, 327.6)	0.75 (0.00, 1.50)	7.5 (5.0, 12.7)	6.6 (4.3, 10.0)	0.53 (-0.25, 1.31)

CI confidence interval, DALY disability-adjusted life year, T2DM type 2 diabetes mellitus, EAPC estimated annual percentage change, HBV hepatitis B virus, SDI socio-demographic index, UI uncertainty interval.

Table S4. Prevalent cases, DALYs, and deaths of liver cancer due to HBV–T2DM comorbidity in 1990 and 2019 and relative changes between 1990 and 2019, stratified by SDI, region, and country

Location	Prevalent cases			DALYs			Deaths		
	1990 No. (95% UI)	2019 No. (95% UI)	Relative change % (95% CI)	1990 No. (95% UI)	2019 No. (95% UI)	Relative change % (95% CI)	1990 No. (95% UI)	2019 No. (95% UI)	Relative change % (95% CI)
**Global**	3048.2 (2563.4, 3577.1)	8289.7 (7040.7, 9625.1)	171.95 (112.66, 239.31)	84978.6 (72598.2, 99598.3)	152945.2 (129587.1, 178641.4)	79.98 (41.58, 124.38)	2495.1 (2128.5, 2916.8)	5058.6 (4270.4, 5902.6)	102.74 (59.44, 152.53)
**SDI**									
Low SDI	33.4 (27.3, 40.6)	105.2 (84.3, 128.6)	214.70 (131.48, 316.97)	971.2 (793.9, 1164.0)	3042.6 (2453.6, 3707.2)	213.29 (132.63, 311.27)	29.3 (23.6, 35.7)	89.6 (71.0, 110.0)	205.91 (122.45, 308.93)
Low-middle SDI	212.2 (182.2, 247.2)	619.7 (532.3, 717.3)	191.98 (132.23, 259.37)	6207.6 (5297.1, 7239.4)	16443.9 (14094.4, 18871.6)	164.90 (110.68, 225.87)	179.2 (153.8, 207.6)	523.7 (448.2, 606.8)	192.21 (132.75, 259.56)
Middle SDI	1416.1 (1170.7, 1688.4)	4075.2 (3355.5, 4905.8)	187.78 (117.41, 272.25)	40887.8 (34400.2, 48814.7)	81762.8 (67565.8, 97873.1)	99.97 (52.37, 156.38)	1173.9 (985.3, 1406.2)	2660.3 (2211.3, 3189.8)	126.63 (72.31, 191.02)
High-middle SDI	1087.1 (898.5, 1296.2)	2215.9 (1780.1, 2683.6)	103.83 (52.48, 166.51)	30064.0 (24823.8, 35749.7)	38370.7 (31628.7, 46502.9)	27.63 (-3.75, 65.18)	903.5 (748.3, 1073.2)	1270.4 (1047.9, 1533.4)	40.62 (6.42, 81.41)
High SDI	338.2 (296.7, 383.9)	2245.5 (1867.7, 2671.0)	563.94 (425.54, 717.93)	6647.5 (5846.5, 7574.9)	21938.5 (18539.9, 25655.8)	230.03 (165.11, 303.59)	217.1 (187.6, 251.5)	865.1 (719.4, 1023.0)	298.44 (212.85, 398.85)
**Regions**									

Location	Prevalent cases			DALYs			Deaths		
	1990 No. (95% UI)	2019 No. (95% UI)	Relative change % (95% CI)	1990 No. (95% UI)	2019 No. (95% UI)	Relative change % (95% CI)	1990 No. (95% UI)	2019 No. (95% UI)	Relative change % (95% CI)
High-income Asia Pacific	154.0 (135.5, 174.2)	1170.1 (941.6, 1421.9)	659.64 (485.86, 855.79)	2735.4 (2389.6, 3116.8)	9383.7 (7938.6, 10948.4)	243.05 (175.37, 320.46)	87.2 (75.9, 99.7)	380.1 (317.6, 445.9)	335.86 (246.63, 438.32)
Central Asia	4.7 (3.5, 6.3)	42.9 (30.6, 57.5)	815.90 (473.96, 1307.28)	127.5 (94.9, 172.7)	1191.3 (856.2, 1589.7)	834.49 (489.31, 1329.35)	4.1 (3.0, 5.6)	36.7 (26.2, 49.3)	792.24 (455.24, 1288.25)
East Asia	2589.0 (2119.7, 3125.7)	5849.1 (4717.0, 7108.3)	125.92 (67.46, 197.25)	73939.9 (61224.1, 89126.0)	108747.6 (88445.7, 133364.2)	47.08 (9.32, 93.09)	2143.4 (1778.3, 2574.4)	3535.9 (2874.6, 4302.6)	64.97 (23.31, 115.44)
South Asia	74.7 (61.6, 88.6)	358.2 (293.4, 432.5)	379.27 (261.54, 520.43)	2159.4 (1806.8, 2530.4)	9815.4 (8140.1, 11734.5)	354.54 (249.28, 479.98)	63.3 (52.0, 75.2)	313.6 (257.2, 379.4)	395.63 (273.05, 542.63)
Southeast Asia	81.1 (67.3, 96.1)	446.1 (336.3, 586.2)	450.02 (278.29, 647.89)	2343.6 (1950.4, 2750.4)	10887.4 (8270.3, 14099.0)	364.56 (224.75, 523.94)	68.6 (55.9, 82.3)	356.8 (264.4, 471.5)	419.87 (250.23, 618.03)
Australasia	1.1 (0.8, 1.4)	11.2 (7.9, 16.0)	957.69 (527.46, 1535.75)	22.0 (16.5, 28.6)	163.1 (118.8, 224.1)	640.56 (364.89, 1010.77)	0.7 (0.5, 1.0)	6.2 (4.4, 8.5)	733.77 (406.10, 1201.73)
Caribbean	9.7 (7.4, 12.4)	19.1 (13.5, 26.1)	96.49 (23.93, 194.72)	256.8 (196.3, 325.0)	478.8 (333.6, 657.5)	86.49 (15.82, 179.67)	8.7 (6.5, 11.4)	16.6 (11.5, 22.9)	90.70 (16.52, 192.01)
Central Europe	40.5 (31.7, 50.9)	66.8 (47.8, 92.7)	65.01 (2.92, 144.47)	1058.6 (839.3, 1320.7)	1510.2 (1107.4, 2078.4)	42.65 (-8.94, 107.79)	37.9 (29.6, 47.9)	59.4 (42.2, 82.5)	56.80 (-2.83, 133.60)

Location	Prevalent cases			DALYs			Deaths		
	1990 No. (95% UI)	2019 No. (95% UI)	Relative change % (95% CI)	1990 No. (95% UI)	2019 No. (95% UI)	Relative change % (95% CI)	1990 No. (95% UI)	2019 No. (95% UI)	Relative change % (95% CI)
Eastern Europe	15.1 (12.9, 17.7)	50.3 (40.0, 63.0)	233.77 (145.94, 334.09)	399.4 (340.3, 468.5)	1253.1 (998.6, 1569.0)	213.73 (131.68, 307.64)	13.1 (11.1, 15.6)	43.2 (34.3, 54.1)	229.15 (141.98, 332.73)
Western Europe	76.7 (59.1, 97.7)	398.2 (294.2, 541.1)	418.93 (233.99, 662.33)	1433.8 (1098.1, 1827.7)	4423.6 (3263.4, 5869.9)	208.53 (102.69, 349.03)	53.9 (40.9, 70.6)	184.1 (133.9, 249.4)	241.43 (117.10, 410.16)
Andean Latin America	3.9 (3.1, 4.7)	14.7 (10.8, 19.1)	282.99 (162.25, 433.57)	104.8 (86.0, 126.6)	363.0 (267.6, 471.0)	246.25 (138.39, 377.02)	3.5 (2.8, 4.3)	13.7 (9.9, 18.0)	293.94 (163.62, 462.90)
Central Latin America	11.0 (8.6, 14.0)	51.2 (38.6, 70.1)	364.68 (201.00, 579.64)	306.9 (239.8, 388.2)	1267.5 (948.0, 1723.3)	313.03 (168.49, 503.02)	9.3 (7.1, 12.1)	44.3 (33.0, 61.2)	375.42 (200.60, 609.80)
Southern Latin America	1.9 (1.3, 2.6)	10.7 (7.0, 16.1)	472.27 (204.27, 859.92)	47.7 (34.2, 65.5)	233.7 (162.2, 327.0)	389.76 (189.72, 682.90)	1.7 (1.2, 2.4)	9.2 (6.3, 13.2)	441.95 (204.80, 813.70)
Tropical Latin America	6.6 (5.7, 7.5)	30.2 (25.6, 35.3)	357.66 (266.48, 462.48)	186.0 (161.6, 211.2)	761.2 (648.7, 886.0)	309.20 (229.35, 400.65)	5.5 (4.7, 6.3)	26.1 (22.0, 30.7)	375.44 (276.97, 489.99)
North Africa and Middle East	34.8 (27.7, 42.8)	235.5 (178.8, 304.0)	577.38 (367.89, 847.68)	959.1 (769.3, 1185.1)	5298.7 (3960.8, 6818.4)	452.44 (279.70, 675.36)	30.2 (23.7, 37.7)	170.1 (126.6, 222.3)	463.80 (279.82, 702.98)
High-income North America	34.9 (30.4, 40.1)	300.8 (227.7, 393.1)	761.42 (506.51, 1047.26)	607.9 (527.2, 696.3)	3688.1 (2981.4, 4561.0)	506.73 (361.89, 671.97)	21.4 (18.4, 24.9)	139.3 (112.4, 171.5)	549.74 (390.64, 729.65)

Location	Prevalent cases			DALYs			Deaths		
	1990 No. (95% UI)	2019 No. (95% UI)	Relative change % (95% CI)	1990 No. (95% UI)	2019 No. (95% UI)	Relative change % (95% CI)	1990 No. (95% UI)	2019 No. (95% UI)	Relative change % (95% CI)
Oceania	1.3 (1.1, 1.7)	5.1 (4.0, 6.5)	279.81 (166.23, 424.75)	38.2 (30.3, 46.9)	141.0 (110.8, 180.0)	268.76 (160.24, 406.19)	1.1 (0.9, 1.4)	4.3 (3.3, 5.6)	278.81 (160.94, 431.06)
Central Sub-Saharan Africa	1.8 (1.3, 2.4)	5.4 (3.8, 7.7)	205.13 (80.57, 381.65)	54.1 (39.6, 71.8)	165.1 (115.9, 231.9)	205.13 (83.44, 374.49)	1.4 (1.0, 1.9)	4.2 (2.9, 6.1)	195.71 (68.08, 378.26)
Eastern Sub-Saharan Africa	4.3 (3.2, 5.8)	13.4 (9.8, 17.8)	214.97 (100.90, 378.12)	127.4 (95.8, 169.6)	399.3 (291.9, 527.2)	213.40 (102.01, 369.43)	3.7 (2.7, 5.0)	11.3 (8.3, 15.1)	208.13 (94.99, 374.07)
Southern Sub-Saharan Africa	8.5 (5.7, 14.7)	36.6 (30.5, 43.6)	330.24 (172.23, 770.73)	254.6 (174.1, 436.7)	1085.8 (910.7, 1292.4)	326.46 (172.15, 741.80)	7.0 (4.7, 12.2)	30.3 (25.3, 36.2)	334.19 (172.86, 799.11)
Western Sub-Saharan Africa	13.9 (11.0, 17.0)	37.7 (29.5, 47.3)	172.21 (94.21, 270.88)	395.5 (315.2, 482.1)	1076.3 (842.6, 1344.1)	172.15 (95.04, 269.24)	12.4 (9.8, 15.2)	33.2 (25.9, 41.5)	168.33 (91.58, 265.35)
**Country**									
Afghanistan	5.2 (3.6, 7.2)	12.5 (8.7, 17.7)	141.23 (40.95, 292.99)	149.3 (104.5, 205.4)	374.7 (261.1, 533.8)	150.92 (45.45, 307.46)	4.5 (3.2, 6.3)	9.8 (6.8, 13.6)	115.01 (26.91, 250.38)
Albania	0.5 (0.4, 0.7)	1.7 (1.0, 2.5)	222.74 (73.30, 416.28)	13.8 (10.6, 17.6)	38.6 (24.5, 57.8)	180.32 (53.00, 339.89)	0.5 (0.3, 0.6)	1.5 (0.9, 2.2)	226.41 (72.20, 436.78)
Algeria	0.9 (0.7, 1.2)	10.1 (7.0, 14.0)	982.30 (552.73, 1588.75)	24.9 (18.1, 33.0)	215.4 (150.1, 297.1)	763.97 (424.42, 1236.25)	0.8 (0.6, 1.1)	7.3 (5.0, 10.3)	826.42 (444.39, 1382.91)
American Samoa	0.0 (0.0, 0.0)	0.2 (0.1, 0.2)	519.82 (295.36, 845.98)	0.8 (0.6, 1.1)	4.7 (3.6, 6.4)	482.37 (269.31, 793.81)	0.0 (0.0, 0.0)	0.1 (0.1, 0.2)	521.05 (297.99, 845.87)
Andorra	0.0 (0.0, 0.0)	0.1 (0.1, 0.2)	765.46 (319.52,	0.2 (0.1, 0.3)	1.2 (0.8, 1.9)	455.62 (167.62,	0.0 (0.0, 0.0)	0.0 (0.0, 0.1)	505.32 (189.13,

Location	Prevalent cases			DALYs			Deaths		
	1990 No. (95% UI)	2019 No. (95% UI)	Relative change % (95% CI)	1990 No. (95% UI)	2019 No. (95% UI)	Relative change % (95% CI)	1990 No. (95% UI)	2019 No. (95% UI)	Relative change % (95% CI)
			1573.11)			978.69)			1083.28)
Angola	0.3 (0.2, 0.4)	1.1 (0.7, 1.5)	250.74 (95.06, 499.66)	9.4 (6.5, 13.2)	32.5 (21.4, 46.3)	245.34 (93.59, 475.12)	0.2 (0.2, 0.3)	0.8 (0.6, 1.2)	239.84 (88.90, 472.30)
Antigua and Barbuda	0.0 (0.0, 0.0)	0.0 (0.0, 0.1)	21.62 (-25.81, 86.56)	0.8 (0.6, 1.0)	0.9 (0.7, 1.3)	13.86 (-29.39, 72.73)	0.0 (0.0, 0.0)	0.0 (0.0, 0.0)	12.29 (-34.01, 78.22)
Argentina	1.2 (0.8, 1.6)	5.5 (3.6, 8.3)	372.51 (144.45, 715.48)	29.7 (20.7, 42.1)	125.6 (88.5, 176.4)	323.26 (147.69, 597.88)	1.1 (0.7, 1.5)	4.9 (3.4, 7.1)	359.17 (159.18, 676.38)
Armenia	0.1 (0.1, 0.1)	2.0 (1.4, 2.9)	1948.29 (1080.95, 3111.45)	2.5 (1.9, 3.2)	50.5 (33.8, 72.0)	1939.53 (1083.62, 3084.82)	0.1 (0.1, 0.1)	1.9 (1.2, 2.7)	2193.59 (1191.47, 3564.58)
Australia	0.8 (0.6, 1.1)	8.6 (5.4, 13.1)	970.32 (449.31, 1711.94)	17.7 (12.6, 23.8)	136.6 (94.2, 195.6)	669.98 (350.95, 1129.37)	0.6 (0.4, 0.9)	5.2 (3.6, 7.5)	764.03 (386.62, 1353.91)
Austria	0.6 (0.4, 0.8)	3.4 (2.1, 5.2)	475.82 (190.01, 890.24)	11.1 (7.7, 15.6)	38.1 (24.7, 58.2)	244.07 (76.28, 489.54)	0.4 (0.3, 0.6)	1.6 (1.0, 2.4)	294.14 (99.77, 596.65)
Azerbaijan	0.1 (0.1, 0.2)	2.7 (1.8, 4.0)	1835.48 (997.06, 2949.31)	3.5 (2.7, 4.7)	73.5 (49.6, 106.6)	1976.61 (1083.36, 3191.93)	0.1 (0.1, 0.2)	2.3 (1.6, 3.5)	1953.23 (1006.54, 3274.39)
Bahamas	0.1 (0.1, 0.1)	0.2 (0.1, 0.2)	110.66 (30.34, 220.10)	2.3 (1.7, 2.9)	4.6 (3.2, 6.3)	99.75 (24.57, 202.03)	0.1 (0.1, 0.1)	0.1 (0.1, 0.2)	108.80 (27.46, 219.75)
Bahrain	0.1 (0.0, 0.1)	1.4 (1.0, 2.0)	2129.58 (1225.43, 3358.33)	1.7 (1.3, 2.2)	26.9 (18.2, 38.0)	1504.66 (842.25, 2394.64)	0.1 (0.0, 0.1)	0.8 (0.6, 1.2)	1499.11 (827.92, 2434.97)

Location	Prevalent cases			DALYs			Deaths		
	1990<br>No. (95% UI)	2019<br>No. (95% UI)	Relative change<br>% (95% CI)	1990<br>No. (95% UI)	2019<br>No. (95% UI)	Relative change<br>% (95% CI)	1990<br>No. (95% UI)	2019<br>No. (95% UI)	Relative change<br>% (95% CI)
Bangladesh	5.7 (4.0, 7.6)	18.7 (13.2, 26.0)	228.85 (97.79, 418.00)	173.0 (121.6, 236.5)	517.3 (365.8, 718.1)	199.11 (79.61, 377.59)	4.0 (2.9, 5.4)	14.8 (10.3, 21.3)	270.43 (116.20, 492.42)
Barbados	0.1 (0.0, 0.1)	0.2 (0.1, 0.2)	171.02 (50.67, 381.06)	1.6 (1.0, 2.3)	4.0 (2.8, 5.6)	150.06 (40.40, 336.55)	0.1 (0.0, 0.1)	0.2 (0.1, 0.2)	160.01 (40.12, 384.07)
Belarus	0.5 (0.4, 0.7)	1.8 (1.1, 2.8)	230.73 (63.34, 460.46)	13.4 (10.0, 18.2)	38.9 (23.6, 61.4)	190.09 (42.85, 394.43)	0.5 (0.3, 0.6)	1.4 (0.8, 2.2)	190.23 (42.90, 400.89)
Belgium	1.2 (0.9, 1.7)	5.8 (3.7, 8.7)	370.28 (147.00, 687.05)	24.7 (17.6, 33.9)	76.9 (52.4, 110.5)	210.60 (78.92, 404.10)	1.0 (0.7, 1.4)	3.2 (2.1, 4.6)	225.76 (79.89, 455.92)
Belize	0.0 (0.0, 0.0)	0.1 (0.0, 0.1)	277.81 (143.98, 457.69)	0.5 (0.4, 0.6)	1.8 (1.3, 2.4)	272.80 (144.86, 440.34)	0.0 (0.0, 0.0)	0.1 (0.0, 0.1)	259.99 (129.43, 441.90)
Benin	0.4 (0.3, 0.5)	1.2 (0.8, 1.7)	199.23 (74.92, 370.49)	11.5 (8.5, 15.2)	35.1 (23.7, 50.1)	203.69 (75.07, 377.48)	0.4 (0.3, 0.5)	1.0 (0.7, 1.5)	182.00 (69.28, 339.14)
Bermuda	0.0 (0.0, 0.0)	0.0 (0.0, 0.0)	40.78 (-17.47, 123.56)	0.6 (0.4, 0.8)	0.6 (0.4, 0.8)	2.96 (-39.92, 62.76)	0.0 (0.0, 0.0)	0.0 (0.0, 0.0)	23.13 (-30.17, 102.47)
Bhutan	0.0 (0.0, 0.0)	0.1 (0.1, 0.2)	507.75 (177.55, 1214.51)	0.5 (0.3, 0.8)	2.7 (1.6, 4.2)	457.35 (148.73, 1121.00)	0.0 (0.0, 0.0)	0.1 (0.1, 0.1)	563.44 (200.21, 1341.02)
Bolivia (Plurinational State of)	0.7 (0.5, 1.0)	3.3 (2.3, 4.5)	364.18 (176.97, 632.19)	19.7 (14.1, 26.6)	84.8 (58.7, 118.7)	331.46 (155.56, 583.15)	0.7 (0.5, 0.9)	3.2 (2.2, 4.4)	385.78 (186.38, 673.16)
Bosnia and Herzegovina	1.2 (0.9, 1.6)	5.7 (3.7, 8.7)	370.12 (150.59, 675.41)	32.5 (23.9, 43.4)	135.8 (89.0, 205.2)	317.67 (124.60, 586.21)	1.1 (0.8, 1.5)	5.4 (3.5, 8.3)	402.25 (166.01, 732.04)
Botswana	0.0 (0.0, 0.1)	0.2 (0.1, 0.3)	835.85 (-4265.24,	0.6 (0.3, 1.6)	5.7 (3.6, 8.7)	843.09 (-4203.42,	0.0 (0.0, 0.0)	0.2 (0.1, 0.2)	786.9 (-3915.37,

Location	Prevalent cases			DALYs			Deaths		
	1990 No. (95% UI)	2019 No. (95% UI)	Relative change % (95% CI)	1990 No. (95% UI)	2019 No. (95% UI)	Relative change % (95% CI)	1990 No. (95% UI)	2019 No. (95% UI)	Relative change % (95% CI)
			5242.86)			5468.1)			5218.34)
Brazil	6.5 (5.7, 7.4)	29.8 (25.4, 34.8)	357.52 (267.34, 460.93)	183.8 (159.9, 208.3)	751.4 (640.6, 874.4)	308.76 (229.52, 399.30)	5.4 (4.7, 6.2)	25.8 (21.7, 30.2)	375.56 (278.42, 487.96)
Brunei Darussalam	0.1 (0.1, 0.2)	1.3 (1.0, 1.7)	833.30 (525.30, 1247.32)	3.8 (2.9, 4.9)	29.5 (22.3, 38.2)	681.26 (424.16, 1027.65)	0.1 (0.1, 0.1)	0.8 (0.6, 1.1)	670.56 (412.67, 1023.17)
Bulgaria	4.8 (3.4, 6.5)	5.7 (3.8, 8.6)	18.76 (-36.53, 96.23)	126.0 (92.6, 169.4)	141.4 (94.1, 211.4)	12.23 (-39.14, 82.71)	4.4 (3.1, 6.0)	5.2 (3.5, 7.9)	20.29 (-35.66, 99.56)
Burkina Faso	0.4 (0.3, 0.5)	0.9 (0.6, 1.2)	132.38 (42.35, 257.34)	11.2 (8.2, 14.8)	26.7 (18.4, 37.2)	137.81 (43.59, 268.88)	0.4 (0.2, 0.5)	0.7 (0.5, 1.0)	114.03 (30.92, 233.27)
Burundi	0.2 (0.1, 0.2)	0.4 (0.2, 0.6)	104.09 (-10.87, 278.37)	5.2 (3.4, 7.4)	10.5 (6.4, 17.8)	101.04 (-12.72, 272.39)	0.2 (0.1, 0.2)	0.3 (0.2, 0.5)	94.63 (-14.93, 258.48)
Cabo Verde	0.0 (0.0, 0.0)	0.5 (0.4, 0.7)	5250.34 (3539.90, 7469.45)	0.3 (0.2, 0.3)	14.7 (11.0, 19.4)	5625.29 (3756.09, 7998.59)	0.0 (0.0, 0.0)	0.5 (0.4, 0.6)	4561.54 (3045.29, 6601.71)
Cambodia	1.7 (1.2, 2.1)	10.0 (7.1, 13.7)	503.44 (278.28, 806.75)	51.2 (38.5, 66.0)	282.6 (201.2, 388.6)	451.84 (244.92, 730.61)	1.2 (0.9, 1.6)	7.7 (5.4, 10.8)	534.87 (287.84, 876.50)
Cameroon	0.1 (0.1, 0.1)	0.4 (0.3, 0.6)	404.82 (167.42, 841.22)	2.4 (1.6, 3.8)	12.2 (7.9, 17.6)	413.80 (168.89, 875.00)	0.1 (0.0, 0.1)	0.4 (0.2, 0.5)	377.69 (154.56, 812.24)
Canada	0.7 (0.5, 0.9)	9.5 (6.0, 14.7)	1323.90 (614.61, 2353.32)	12.0 (8.4, 17.1)	110.3 (73.8, 163.0)	821.53 (407.05, 1460.38)	0.4 (0.3, 0.6)	4.4 (2.9, 6.7)	961.40 (458.05, 1749.75)

Location	Prevalent cases			DALYs			Deaths		
	1990 No. (95% UI)	2019 No. (95% UI)	Relative change % (95% CI)	1990 No. (95% UI)	2019 No. (95% UI)	Relative change % (95% CI)	1990 No. (95% UI)	2019 No. (95% UI)	Relative change % (95% CI)
Central African Republic	0.2 (0.1, 0.2)	0.4 (0.2, 0.6)	133.96 (-3.44, 354.14)	4.8 (3.0, 7.0)	11.0 (6.1, 18.6)	131.94 (-5.25, 353.38)	0.1 (0.1, 0.2)	0.3 (0.2, 0.5)	131.17 (-2.09, 355.45)
Chad	0.6 (0.4, 0.8)	1.4 (1.0, 1.9)	133.82 (41.88, 272.36)	16.5 (11.6, 22.4)	39.1 (27.3, 53.6)	137.67 (43.63, 276.79)	0.5 (0.4, 0.7)	1.2 (0.8, 1.6)	120.92 (35.20, 248.44)
Chile	0.6 (0.4, 0.9)	5.0 (3.2, 7.7)	711.87 (320.00, 1267.00)	15.8 (11.6, 21.8)	101.5 (70.7, 143.6)	540.80 (275.94, 923.55)	0.5 (0.4, 0.7)	4.0 (2.7, 5.8)	643.54 (317.70, 1137.29)
China	2547.3 (2078.2, 3081.3)	5704.8 (4568.4, 6964.9)	123.96 (65.00, 196.55)	72803.6 (60040.6, 87873.3)	105812.8 (85541.7, 130497.9)	45.34 (7.37, 92.03)	2110.5 (1748.0, 2542.6)	3441.0 (2783.6, 4205.0)	63.04 (21.23, 114.03)
Colombia	2.0 (1.6, 2.6)	9.2 (5.9, 13.9)	352.88 (146.35, 620.81)	56.7 (43.9, 72.3)	213.2 (136.1, 320.5)	275.75 (105.25, 487.82)	1.7 (1.3, 2.3)	8.0 (5.0, 11.9)	368.80 (150.66, 670.83)
Comoros	0.0 (0.0, 0.0)	0.0 (0.0, 0.1)	240.78 (12.14, 1005.07)	0.4 (0.2, 0.8)	1.4 (0.8, 2.4)	237.32 (9.02, 1024.91)	0.0 (0.0, 0.0)	0.0 (0.0, 0.1)	236.92 (13.09, 894.77)
Congo	0.1 (0.1, 0.2)	0.4 (0.2, 0.6)	162.14 (31.83, 389.91)	4.4 (2.8, 6.7)	11.4 (7.4, 17.4)	158.09 (31.36, 377.31)	0.1 (0.1, 0.2)	0.3 (0.2, 0.5)	163.42 (31.95, 394.85)
Cook Islands	0.0 (0.0, 0.0)	0.1 (0.1, 0.2)	345.05 (187.26, 559.82)	0.7 (0.5, 0.9)	2.6 (1.9, 3.5)	277.24 (141.62, 464.85)	0.0 (0.0, 0.0)	0.1 (0.1, 0.1)	310.83 (166.78, 507.88)
Costa Rica	0.3 (0.2, 0.4)	1.8 (1.2, 2.8)	537.44 (240.29, 940.06)	7.6 (5.7, 9.9)	41.4 (26.8, 62.8)	447.62 (195.12, 784.15)	0.2 (0.2, 0.3)	1.4 (0.9, 2.2)	494.49 (210.27, 894.33)
Croatia	1.2 (0.9, 1.7)	3.8 (2.5, 5.8)	214.41 (66.44, 421.72)	29.1 (21.1, 39.5)	68.7 (44.5, 102.9)	136.42 (25.63, 290.93)	1.1 (0.8, 1.4)	2.8 (1.8, 4.2)	165.48 (39.88, 341.14)
Cuba	5.1 (3.9, 6.6)	6.0 (4.0, 8.6)	17.56 (-32.12,	128.4 (98.9,	129.5 (87.9,	0.86 (-41.43,	4.6 (3.4, 6.1)	5.3 (3.5, 7.6)	15.11 (-34.62,

Location	Prevalent cases			DALYs			Deaths		
	1990 No. (95% UI)	2019 No. (95% UI)	Relative change % (95% CI)	1990 No. (95% UI)	2019 No. (95% UI)	Relative change % (95% CI)	1990 No. (95% UI)	2019 No. (95% UI)	Relative change % (95% CI)
			82.97)	164.4)	187.6)	55.77)			82.98)
Cyprus	0.1 (0.1, 0.2)	0.5 (0.4, 0.8)	392.86 (177.35, 745.38)	2.6 (1.8, 3.8)	7.2 (4.9, 10.4)	174.26 (53.49, 366.92)	0.1 (0.1, 0.1)	0.3 (0.2, 0.4)	205.03 (59.58, 447.06)
Czechia	4.0 (2.8, 5.6)	8.3 (5.5, 12.4)	106.92 (10.91, 245.36)	103.6 (75.6, 141.6)	176.3 (117.2, 261.6)	70.27 (-7.01, 178.23)	3.8 (2.7, 5.3)	7.3 (4.7, 10.9)	91.22 (0.99, 222.00)
Cote d'Ivoire	1.0 (0.7, 1.3)	3.0 (2.0, 4.3)	205.70 (75.22, 394.73)	28.7 (20.0, 38.7)	87.0 (57.8, 124.2)	203.65 (74.63, 392.41)	0.8 (0.6, 1.1)	2.5 (1.7, 3.6)	205.49 (78.16, 389.06)
Democratic People's Republic of Korea	21.5 (15.5, 29.6)	58.0 (39.6, 81.7)	169.38 (57.04, 332.71)	615.4 (434.7, 847.4)	1573.3 (1062.5, 2253.9)	155.65 (46.25, 317.26)	17.6 (12.7, 24.0)	47.6 (33.1, 66.1)	170.88 (62.05, 329.43)
Democratic Republic of the Congo	1.1 (0.8, 1.5)	3.4 (2.3, 5.1)	207.64 (66.42, 411.76)	33.8 (23.7, 46.3)	104.8 (69.4, 154.3)	210.26 (68.78, 413.29)	0.9 (0.6, 1.2)	2.6 (1.7, 3.9)	196.97 (56.38, 405.07)
Denmark	0.5 (0.4, 0.8)	2.9 (1.8, 4.5)	440.16 (169.28, 837.49)	6.3 (4.4, 8.8)	33.4 (22.4, 48.1)	425.40 (196.87, 768.36)	0.2 (0.2, 0.4)	1.3 (0.9, 2.0)	454.37 (196.27, 871.84)
Djibouti	0.0 (0.0, 0.0)	0.1 (0.0, 0.1)	755.81 (162.89, 2484.24)	0.3 (0.2, 0.5)	2.3 (1.2, 4.2)	742.13 (155.90, 2393.68)	0.0 (0.0, 0.0)	0.1 (0.0, 0.1)	796.50 (182.46, 2598.13)
Dominica	0.0 (0.0, 0.1)	0.0 (0.0, 0.1)	5.07 (-38.64, 67.89)	1.1 (0.8, 1.5)	1.2 (0.8, 1.7)	3.56 (-38.21, 62.35)	0.0 (0.0, 0.1)	0.0 (0.0, 0.1)	2.42 (-42.02, 70.06)
Dominican Republic	0.4 (0.3, 0.5)	2.7 (1.6, 4.6)	575.71 (184.30, 1048.21)	11.7 (8.9, 14.9)	74.4 (42.9, 125.6)	534.27 (165.58, 978.05)	0.3 (0.3, 0.4)	2.3 (1.4, 3.9)	600.98 (204.75, 1084.98)
Ecuador	0.7 (0.6, 0.8)	5.3 (3.7, 7.1)	648.34 (388.26, 952.03)	19.3 (15.9, 22.8)	128.0 (90.3, 171.8)	564.68 (337.05, 832.13)	0.6 (0.5, 0.8)	5.0 (3.5, 6.9)	678.68 (395.23, 1012.93)

Location	Prevalent cases			DALYs			Deaths		
	1990 No. (95% UI)	2019 No. (95% UI)	Relative change % (95% CI)	1990 No. (95% UI)	2019 No. (95% UI)	Relative change % (95% CI)	1990 No. (95% UI)	2019 No. (95% UI)	Relative change % (95% CI)
Egypt	6.0 (4.1, 8.8)	43.7 (26.2, 69.4)	623.00 (238.46, 1216.28)	175.4 (120.2, 251.7)	1151.1 (683.0, 1789.9)	556.11 (215.71, 1070.58)	5.0 (3.4, 7.4)	34.3 (20.5, 55.3)	580.31 (209.18, 1159.33)
El Salvador	0.3 (0.2, 0.3)	0.7 (0.4, 1.0)	147.56 (31.79, 302.70)	7.5 (5.7, 9.6)	16.3 (10.4, 24.8)	118.41 (16.57, 249.83)	0.2 (0.2, 0.3)	0.6 (0.4, 0.9)	167.84 (37.99, 349.83)
Equatorial Guinea	0.0 (0.0, 0.0)	0.1 (0.0, 0.1)	240.56 (47.20, 545.03)	0.6 (0.4, 0.9)	2.0 (1.1, 3.2)	227.35 (37.54, 512.15)	0.0 (0.0, 0.0)	0.1 (0.0, 0.1)	217.13 (35.51, 508.91)
Eritrea	0.1 (0.0, 0.1)	0.3 (0.2, 0.5)	302.50 (78.29, 700.92)	2.2 (1.4, 3.5)	8.6 (5.0, 13.7)	283.82 (68.15, 672.33)	0.1 (0.0, 0.1)	0.2 (0.1, 0.4)	285.10 (74.56, 661.11)
Estonia	0.2 (0.1, 0.2)	0.6 (0.4, 0.9)	250.16 (87.75, 478.92)	4.3 (3.2, 5.9)	11.3 (7.1, 17.0)	160.52 (37.32, 332.94)	0.1 (0.1, 0.2)	0.5 (0.3, 0.7)	204.30 (61.38, 408.38)
Eswatini	0.1 (0.0, 0.1)	1.0 (0.2, 1.9)	1553.78 (23.41, 6306.05)	1.8 (1.0, 3.9)	30.4 (6.9, 60.2)	1597.78 (6.54, 6534.94)	0.0 (0.0, 0.1)	0.8 (0.2, 1.6)	1527.34 (46.98, 6336.78)
Ethiopia	1.0 (0.7, 1.6)	1.7 (1.3, 2.3)	67.36 (-1.67, 194.02)	31.9 (20.9, 48.8)	50.1 (37.3, 66.5)	57.17 (-8.12, 177.46)	0.9 (0.6, 1.3)	1.5 (1.1, 2.0)	67.03 (-0.76, 184.11)
Fiji	0.4 (0.3, 0.5)	1.9 (1.3, 2.6)	411.31 (217.73, 674.87)	10.5 (7.9, 13.6)	51.7 (36.6, 71.9)	390.56 (204.09, 644.57)	0.3 (0.2, 0.4)	1.6 (1.1, 2.2)	421.00 (223.63, 689.36)
Finland	1.2 (0.8, 1.6)	7.6 (4.7, 11.9)	561.12 (226.41, 1036.60)	16.9 (12.0, 23.4)	61.0 (41.0, 90.1)	261.12 (98.12, 497.33)	0.6 (0.4, 0.9)	2.7 (1.7, 4.1)	345.53 (127.39, 681.22)
France	6.8 (4.7, 9.6)	34.5 (21.5, 52.8)	405.54 (153.00, 776.67)	143.9 (99.4, 203.0)	449.1 (291.7, 656.8)	212.12 (71.26, 429.08)	5.3 (3.6, 7.6)	18.4 (12.0, 27.2)	245.60 (85.95, 501.89)
Gabon	0.1 (0.0, 0.1)	0.2 (0.1, 0.3)	204.35 (42.20,	1.8 (1.2, 2.6)	5.4 (3.1, 8.4)	195.54 (39.19,	0.1 (0.0, 0.1)	0.2 (0.1, 0.2)	203.69 (37.57,

Location	Prevalent cases			DALYs			Deaths		
	1990 No. (95% UI)	2019 No. (95% UI)	Relative change % (95% CI)	1990 No. (95% UI)	2019 No. (95% UI)	Relative change % (95% CI)	1990 No. (95% UI)	2019 No. (95% UI)	Relative change % (95% CI)
			457.20)			437.30)			463.84)
Gambia	0.3 (0.2, 0.5)	2.2 (1.5, 3.1)	565.19 (284.11, 994.61)	9.8 (6.7, 13.7)	64.7 (44.8, 92.3)	562.32 (280.29, 998.44)	0.3 (0.2, 0.4)	1.9 (1.3, 2.6)	562.06 (291.20, 963.82)
Georgia	0.3 (0.2, 0.4)	2.2 (1.5, 3.3)	715.80 (362.53, 1215.62)	6.9 (5.1, 9.5)	59.7 (40.9, 85.6)	760.24 (400.27, 1282.52)	0.2 (0.2, 0.3)	2.0 (1.4, 2.9)	719.03 (366.40, 1242.76)
Germany	15.4 (11.1, 21.2)	88.0 (55.9, 132.4)	472.12 (199.02, 856.56)	282.1 (204.9, 389.6)	898.4 (608.2, 1300.8)	218.48 (82.23, 417.71)	10.7 (7.6, 15.0)	38.9 (25.7, 57.0)	262.53 (99.74, 504.27)
Ghana	1.4 (1.0, 1.9)	7.2 (5.0, 9.7)	417.87 (214.95, 724.27)	40.1 (28.5, 55.1)	206.8 (142.8, 278.5)	415.71 (213.77, 720.21)	1.2 (0.9, 1.7)	6.1 (4.3, 8.4)	409.71 (208.62, 714.11)
Greece	2.5 (1.9, 3.1)	14.1 (9.7, 19.7)	464.47 (242.67, 745.11)	47.0 (36.1, 58.6)	202.1 (151.3, 263.0)	330.33 (190.90, 512.76)	2.0 (1.5, 2.6)	9.5 (6.9, 12.7)	368.09 (202.11, 593.79)
Greenland	0.0 (0.0, 0.0)	0.0 (0.0, 0.0)	900.34 (380.92, 1757.25)	0.0 (0.0, 0.1)	0.4 (0.2, 0.6)	745.90 (292.48, 1501.67)	0.0 (0.0, 0.0)	0.0 (0.0, 0.0)	929.60 (391.29, 1812.77)
Grenada	0.0 (0.0, 0.1)	0.0 (0.0, 0.1)	11.3 (-31.35, 69.12)	1.1 (0.9, 1.4)	1.2 (0.8, 1.6)	6.2 (-33.41, 59.79)	0.0 (0.0, 0.1)	0.0 (0.0, 0.1)	3.24 (-38.53, 62.91)
Guam	0.0 (0.0, 0.0)	0.3 (0.2, 0.4)	783.15 (507.27, 1140.32)	1.0 (0.8, 1.2)	8.2 (6.3, 10.5)	727.91 (471.20, 1059.33)	0.0 (0.0, 0.0)	0.3 (0.2, 0.3)	771.46 (494.40, 1133.80)
Guatemala	0.9 (0.7, 1.2)	3.0 (2.0, 4.3)	236.11 (91.56, 434.07)	25.7 (19.2, 33.9)	80.4 (54.8, 115.2)	212.63 (81.16, 390.25)	0.7 (0.5, 1.0)	2.5 (1.6, 3.6)	237.19 (85.51, 449.67)
Guinea	3.3 (2.5, 4.2)	9.3 (6.1, 12.8)	186.13 (71.28, 339.75)	92.7 (71.0, 119.0)	269.8 (178.7, 373.2)	191.06 (74.75, 344.29)	3.0 (2.3, 3.9)	8.3 (5.6, 11.4)	174.51 (67.04, 319.66)
Guinea-Bissau	0.1 (0.1, 0.2)	0.3 (0.2, 0.4)	117.26 (19.60,	3.4 (2.3, 5.1)	7.4 (5.0, 10.7)	117.86 (19.05,	0.1 (0.1, 0.1)	0.2 (0.1, 0.3)	105.90 (15.19,

Location	Prevalent cases			DALYs			Deaths		
	1990<br>No. (95% UI)	2019<br>No. (95% UI)	Relative change<br>% (95% CI)	1990<br>No. (95% UI)	2019<br>No. (95% UI)	Relative change<br>% (95% CI)	1990<br>No. (95% UI)	2019<br>No. (95% UI)	Relative change<br>% (95% CI)
			280.61)			285.71)			254.34)
Guyana	0.2 (0.1, 0.2)	0.3 (0.2, 0.4)	60.17 (-9.24, 154.53)	5.0 (3.8, 6.6)	7.8 (5.2, 11.3)	54.78 (-12.27, 145.15)	0.1 (0.1, 0.2)	0.2 (0.2, 0.4)	63.97 (-7.82, 163.06)
Haiti	1.2 (0.7, 1.9)	2.8 (1.6, 4.6)	131.59 (-4.38, 375.9)	34.8 (20.3, 52.4)	79.4 (44.7, 132.1)	128.53 (-5.56, 360.6)	1.1 (0.6, 1.7)	2.5 (1.4, 4.2)	128.15 (-9.68, 380.94)
Honduras	0.7 (0.3, 1.2)	4.7 (1.9, 8.1)	536.39 (95.59, 1574.54)	20.8 (8.3, 34.1)	125.0 (51.2, 216.8)	500.12 (83.28, 1464.91)	0.6 (0.2, 1.1)	4.3 (1.7, 7.4)	570.81 (94.69, 1744.01)
Hungary	5.3 (3.8, 7.3)	5.2 (3.5, 7.6)	-1.45 (-44.16, 60.47)	138.1 (100.9, 186.6)	117.9 (79.7, 170.6)	-14.64 (-51.59, 36.7)	5.1 (3.6, 7.0)	4.8 (3.2, 7.0)	-5.35 (-47.31, 56.22)
Iceland	0.0 (0.0, 0.0)	0.1 (0.1, 0.2)	904.09 (483.94, 1518.88)	0.2 (0.2, 0.3)	1.6 (1.1, 2.3)	542.60 (278.30, 938.69)	0.0 (0.0, 0.0)	0.1 (0.0, 0.1)	587.48 (272.42, 1086.39)
India	65.0 (52.8, 77.8)	332.8 (266.7, 408.8)	411.73 (277.41, 577.07)	1861.8 (1516.1, 2202.2)	9050.1 (7368.7, 11043.9)	386.09 (263.47, 535.29)	56.3 (45.1, 67.8)	295.6 (238.2, 362.3)	424.75 (285.06, 595.67)
Indonesia	5.5 (4.5, 6.5)	21.0 (16.3, 26.6)	281.97 (173.45, 415.30)	161.5 (132.9, 191.6)	565.0 (435.5, 704.0)	249.91 (153.47, 367.34)	4.3 (3.6, 5.2)	17.8 (13.7, 22.3)	312.10 (196.17, 454.88)
Iran (Islamic Republic of)	4.2 (3.5, 4.9)	41.0 (35.0, 47.4)	874.16 (663.98, 1117.02)	108.7 (91.3, 128.0)	764.6 (659.2, 877.4)	603.15 (458.82, 772.39)	3.6 (3.0, 4.3)	27.1 (23.2, 31.4)	648.35 (486.62, 839.08)
Iraq	2.1 (1.5, 2.8)	17.4 (11.8, 24.7)	739.71 (381.89, 1263.85)	57.3 (40.2, 77.6)	397.7 (270.0, 568.0)	594.32 (301.96, 1020.84)	1.8 (1.3, 2.5)	12.6 (8.6, 17.8)	607.54 (307.01, 1053.55)
Ireland	0.1 (0.1, 0.1)	1.8 (1.1, 2.7)	2287.05 (1111.94,	1.5 (1.1, 2.1)	20.0 (13.7, 29.0)	1195.56 (635.11,	0.1 (0.0, 0.1)	0.8 (0.5, 1.2)	1283.59 (638.67,

Location	Prevalent cases			DALYs			Deaths		
	1990 No. (95% UI)	2019 No. (95% UI)	Relative change % (95% CI)	1990 No. (95% UI)	2019 No. (95% UI)	Relative change % (95% CI)	1990 No. (95% UI)	2019 No. (95% UI)	Relative change % (95% CI)
			3969.33)			2031.53)			2323.17)
Israel	0.3 (0.2, 0.4)	1.9 (1.3, 2.9)	542.04 (233.96, 1003.22)	6.8 (4.8, 9.6)	30.0 (21.0, 42.7)	340.93 (155.62, 628.54)	0.3 (0.2, 0.4)	1.2 (0.8, 1.7)	347.40 (141.39, 690.85)
Italy	26.8 (22.6, 31.8)	78.3 (57.9, 103.4)	192.24 (98.54, 299.79)	469.9 (396.7, 559.1)	744.7 (614.7, 902.7)	58.47 (20.40, 104.54)	17.7 (14.7, 21.2)	31.5 (25.8, 38.5)	78.40 (33.79, 132.52)
Jamaica	0.4 (0.3, 0.5)	1.0 (0.7, 1.4)	156.76 (54.50, 294.18)	10.0 (7.7, 12.8)	25.1 (17.2, 34.9)	150.30 (51.61, 279.25)	0.4 (0.3, 0.5)	0.9 (0.6, 1.2)	146.03 (47.32, 284.61)
Japan	114.0 (99.3, 131.7)	339.9 (263.6, 428.7)	198.27 (117.99, 289.34)	1597.6 (1393.2, 1834.9)	2146.8 (1819.9, 2562.7)	34.38 (6.36, 66.55)	53.0 (45.9, 61.1)	107.1 (87.7, 130.0)	102.16 (55.76, 154.52)
Jordan	0.2 (0.1, 0.3)	1.8 (1.3, 2.5)	827.26 (474.59, 1324.01)	5.2 (3.9, 6.9)	36.7 (25.7, 50.2)	601.33 (331.68, 967.47)	0.2 (0.1, 0.2)	1.2 (0.9, 1.7)	656.61 (355.56, 1089.95)
Kazakhstan	1.9 (1.4, 2.5)	8.6 (6.0, 12.1)	360.75 (176.63, 618.40)	51.0 (38.2, 67.5)	228.9 (160.3, 318.2)	348.49 (173.50, 588.56)	1.6 (1.2, 2.2)	7.4 (5.1, 10.4)	359.91 (170.80, 629.39)
Kenya	0.3 (0.2, 0.5)	1.7 (1.0, 2.5)	490.16 (172.52, 1232.21)	8.2 (5.1, 14.7)	52.4 (33.4, 79.2)	540.91 (198.65, 1364.93)	0.2 (0.1, 0.4)	1.5 (1.0, 2.3)	528.20 (196.10, 1307.32)
Kiribati	0.1 (0.1, 0.1)	0.3 (0.2, 0.4)	206.26 (95.08, 362.35)	2.8 (2.0, 3.6)	8.5 (6.1, 11.5)	208.47 (95.48, 365.70)	0.1 (0.1, 0.1)	0.2 (0.2, 0.3)	197.06 (86.87, 353.00)
Kuwait	0.2 (0.1, 0.2)	1.6 (1.1, 2.2)	863.24 (493.90, 1315.92)	4.0 (3.2, 4.9)	23.9 (16.4, 33.5)	494.99 (265.28, 770.51)	0.1 (0.1, 0.1)	0.8 (0.5, 1.1)	570.87 (300.26, 908.42)
Kyrgyzstan	0.1 (0.1, 0.1)	0.4 (0.3, 0.5)	393.31 (204.40, 653.49)	2.0 (1.5, 2.7)	10.6 (7.6, 14.6)	427.68 (227.16, 707.13)	0.1 (0.0, 0.1)	0.3 (0.2, 0.5)	392.15 (190.98, 682.77)

Location	Prevalent cases			DALYs			Deaths		
	1990 No. (95% UI)	2019 No. (95% UI)	Relative change % (95% CI)	1990 No. (95% UI)	2019 No. (95% UI)	Relative change % (95% CI)	1990 No. (95% UI)	2019 No. (95% UI)	Relative change % (95% CI)
Lao People's Democratic Republic	1.1 (0.7, 1.6)	3.1 (2.0, 4.3)	177.01 (53.01, 394.97)	32.6 (20.3, 49.0)	87.1 (58.3, 122.3)	167.39 (47.03, 382.64)	1.0 (0.6, 1.4)	2.6 (1.7, 3.7)	167.60 (47.78, 374.71)
Latvia	0.2 (0.2, 0.3)	0.6 (0.4, 0.9)	185.60 (59.83, 362.67)	5.8 (4.3, 7.9)	14.4 (9.7, 20.8)	148.38 (40.07, 297.81)	0.2 (0.1, 0.3)	0.6 (0.4, 0.8)	175.94 (52.13, 355.17)
Lebanon	0.7 (0.6, 1.0)	5.5 (3.9, 7.9)	648.08 (343.88, 1055.72)	18.8 (14.2, 24.4)	72.7 (50.9, 102.4)	286.06 (133.12, 492.13)	0.6 (0.5, 0.8)	2.7 (1.9, 3.8)	317.99 (146.71, 547.84)
Lesotho	0.2 (0.1, 0.4)	1.5 (0.5, 2.6)	708.94 (12.41, 4116.01)	5.4 (2.8, 12.7)	45.7 (15.1, 80.0)	742.23 (3.02, 4261.63)	0.2 (0.1, 0.4)	1.3 (0.4, 2.3)	703.98 (4.80, 4059.16)
Liberia	0.4 (0.3, 0.5)	0.7 (0.5, 1.2)	98.24 (0.23, 233.82)	10.4 (7.5, 13.8)	21.3 (13.8, 32.9)	105.76 (6.96, 243.18)	0.3 (0.3, 0.5)	0.6 (0.4, 1.0)	80.87 (-7.41, 205.19)
Libya	0.5 (0.4, 0.7)	5.0 (3.3, 7.4)	852.03 (413.64, 1483.54)	14.2 (9.9, 19.4)	119.4 (78.8, 176.0)	740.01 (355.48, 1291.78)	0.4 (0.3, 0.6)	3.6 (2.4, 5.4)	729.66 (348.53, 1295.00)
Lithuania	0.3 (0.2, 0.4)	1.0 (0.7, 1.5)	266.24 (99.91, 494.72)	6.4 (4.7, 8.5)	18.8 (12.4, 27.8)	194.59 (61.80, 375.96)	0.2 (0.2, 0.3)	0.7 (0.5, 1.0)	223.64 (76.58, 429.11)
Luxembourg	0.0 (0.0, 0.0)	0.4 (0.2, 0.5)	1103.56 (526.24, 1967.28)	0.6 (0.4, 0.9)	4.4 (2.8, 6.6)	614.43 (271.10, 1129.58)	0.0 (0.0, 0.0)	0.2 (0.1, 0.3)	655.37 (274.11, 1267.98)
Madagascar	0.3 (0.2, 0.6)	0.8 (0.5, 1.4)	146.56 (3.42, 451.28)	10.3 (6.8, 17.8)	25.5 (15.7, 41.3)	148.42 (8.92, 444.82)	0.3 (0.2, 0.5)	0.7 (0.4, 1.2)	142.14 (-2.32, 461.91)
Malawi	0.3 (0.2, 0.5)	0.7 (0.5, 0.9)	109.01 (16.45, 274.44)	9.4 (6.3, 14.7)	18.6 (12.6, 26.7)	98.96 (7.60, 264.21)	0.3 (0.2, 0.4)	0.5 (0.4, 0.8)	101.57 (8.35, 280.02)
Malaysia	4.4 (3.5, 5.3)	30.2 (21.7,	592.31 (351.71,	120.8 (95.6,	722.3 (515.1,	498.14 (288.63,	3.9 (3.1, 4.7)	24.9 (17.7,	539.85 (320.44,

Location	Prevalent cases			DALYs			Deaths		
	1990 No. (95% UI)	2019 No. (95% UI)	Relative change % (95% CI)	1990 No. (95% UI)	2019 No. (95% UI)	Relative change % (95% CI)	1990 No. (95% UI)	2019 No. (95% UI)	Relative change % (95% CI)
		40.4)	883.85)	146.9)	966.2)	753.06)		32.9)	807.97)
Maldives	0.0 (0.0, 0.0)	0.2 (0.1, 0.2)	636.74 (316.13, 1360.97)	0.6 (0.4, 1.0)	3.8 (2.7, 5.0)	515.32 (244.52, 1135.66)	0.0 (0.0, 0.0)	0.1 (0.1, 0.2)	509.13 (241.03, 1102.89)
Mali	1.7 (1.2, 2.2)	4.7 (3.1, 6.9)	174.55 (52.57, 340.70)	50.5 (36.7, 65.5)	137.5 (87.8, 200.9)	172.54 (50.70, 338.77)	1.5 (1.1, 2.0)	4.0 (2.6, 5.8)	167.31 (48.29, 332.26)
Malta	0.0 (0.0, 0.0)	0.2 (0.1, 0.3)	620.10 (298.34, 1120.03)	0.6 (0.4, 0.9)	2.9 (2.0, 4.3)	375.21 (165.03, 696.58)	0.0 (0.0, 0.0)	0.1 (0.1, 0.2)	427.37 (178.51, 840.81)
Marshall Islands	0.0 (0.0, 0.0)	0.2 (0.1, 0.3)	450.52 (212.92, 781.87)	1.0 (0.7, 1.3)	5.3 (3.5, 7.6)	454.06 (216.02, 780.83)	0.0 (0.0, 0.0)	0.1 (0.1, 0.2)	431.22 (200.42, 775.20)
Mauritania	0.2 (0.1, 0.2)	0.3 (0.2, 0.5)	80.60 (6.21, 178.30)	5.2 (3.9, 6.7)	9.1 (5.9, 12.8)	75.01 (1.01, 172.43)	0.2 (0.1, 0.2)	0.3 (0.2, 0.4)	79.78 (6.88, 177.83)
Mauritius	0.1 (0.1, 0.2)	0.9 (0.6, 1.2)	624.92 (332.09, 982.39)	3.3 (2.6, 4.0)	19.9 (13.3, 28.3)	510.08 (265.04, 804.95)	0.1 (0.1, 0.1)	0.7 (0.5, 1.1)	607.63 (312.38, 987.23)
Mexico	3.4 (2.9, 4.0)	23.9 (18.9, 30.3)	604.79 (417.62, 819.05)	93.0 (79.4, 107.8)	592.2 (470.7, 730.7)	536.92 (376.20, 718.86)	2.9 (2.5, 3.4)	20.8 (16.4, 26.1)	619.55 (426.65, 842.44)
Micronesia (Federated States of)	0.1 (0.0, 0.1)	0.2 (0.1, 0.4)	370.12 (135.71, 709.72)	1.5 (1.0, 2.0)	6.7 (4.0, 10.5)	356.87 (119.32, 702.97)	0.0 (0.0, 0.1)	0.2 (0.1, 0.3)	352.57 (131.16, 669.79)
Monaco	0.0 (0.0, 0.0)	0.1 (0.0, 0.1)	675.55 (302.36, 1325.95)	0.1 (0.1, 0.2)	0.8 (0.5, 1.1)	458.05 (183.42, 932.11)	0.0 (0.0, 0.0)	0.0 (0.0, 0.0)	474.49 (192.77, 999.49)
Mongolia	1.0 (0.7, 1.3)	6.9 (4.5, 10.2)	622.53 (291.47, 1106.97)	26.9 (19.1, 36.9)	195.4 (126.3, 288.9)	626.87 (291.79, 1105.87)	0.9 (0.6, 1.2)	6.2 (4.1, 8.9)	605.99 (296.48, 1069.64)
Montenegro	0.2 (0.1, 0.3)	0.6 (0.4, 0.9)	201.62 (69.06,	5.4 (3.8, 7.5)	14.8 (9.9,	172.33 (53.54,	0.2 (0.1, 0.3)	0.5 (0.4, 0.8)	205.26 (67.80,

Location	Prevalent cases			DALYs			Deaths		
	1990 No. (95% UI)	2019 No. (95% UI)	Relative change % (95% CI)	1990 No. (95% UI)	2019 No. (95% UI)	Relative change % (95% CI)	1990 No. (95% UI)	2019 No. (95% UI)	Relative change % (95% CI)
			401.31)		21.6)	348.02)			418.88)
Morocco	1.0 (0.7, 1.4)	7.7 (5.2, 11.0)	641.25 (325.60, 1105.13)	26.9 (19.3, 36.6)	185.0 (124.0, 266.0)	587.62 (294.95, 1010.98)	1.0 (0.7, 1.3)	6.9 (4.6, 9.8)	617.10 (307.30, 1078.10)
Mozambique	0.4 (0.2, 0.6)	1.7 (1.1, 2.5)	386.61 (134.23, 977.06)	10.4 (6.6, 18.3)	51.5 (31.9, 75.4)	394.06 (140.71, 985.98)	0.3 (0.2, 0.6)	1.5 (0.9, 2.1)	361.15 (122.88, 927.21)
Myanmar	3.1 (1.9, 4.7)	18.1 (12.9, 25.2)	491.24 (227.86, 991.54)	86.4 (56.0, 133.6)	486.8 (346.4, 676.4)	463.37 (214.64, 928.92)	2.8 (1.8, 4.3)	16.0 (11.4, 22.2)	469.58 (220.02, 931.14)
Namibia	0.0 (0.0, 0.1)	0.3 (0.2, 0.4)	451.35 (141.31, 1778.49)	1.4 (0.7, 2.9)	7.8 (5.0, 11.2)	455.09 (138.36, 1888.82)	0.0 (0.0, 0.1)	0.2 (0.2, 0.3)	424.20 (129.32, 1686.72)
Nauru	0.0 (0.0, 0.0)	0.0 (0.0, 0.0)	113.46 (4.86, 282.91)	0.1 (0.1, 0.2)	0.3 (0.2, 0.5)	108.68 (-1.58, 282.98)	0.0 (0.0, 0.0)	0.0 (0.0, 0.0)	101.81 (1.54, 253.74)
Nepal	0.5 (0.3, 0.7)	2.6 (1.6, 4.0)	444.90 (164.12, 873.13)	13.4 (9.0, 19.7)	68.2 (42.4, 106.9)	407.68 (141.64, 825.79)	0.4 (0.3, 0.6)	2.3 (1.4, 3.6)	487.38 (180.49, 956.92)
Netherlands	0.8 (0.6, 1.1)	6.4 (4.2, 9.6)	667.73 (310.68, 1162.35)	14.9 (10.7, 20.5)	70.8 (49.5, 99.7)	374.81 (179.81, 661.33)	0.5 (0.4, 0.8)	2.9 (1.9, 4.1)	437.41 (196.66, 814.67)
New Zealand	0.2 (0.2, 0.3)	2.5 (1.9, 3.3)	921.36 (599.36, 1290.75)	4.2 (3.5, 5.0)	26.1 (21.7, 31.6)	526.61 (376.82, 706.32)	0.1 (0.1, 0.2)	0.9 (0.8, 1.1)	606.37 (432.38, 813.67)
Nicaragua	0.1 (0.1, 0.2)	0.9 (0.6, 1.3)	542.41 (267.32, 917.69)	3.9 (2.9, 5.1)	22.3 (15.1, 32.0)	467.95 (228.90, 792.64)	0.1 (0.1, 0.2)	0.7 (0.5, 1.0)	553.49 (258.12, 972.98)
Niger	0.0 (0.0, 0.0)	0.1 (0.1, 0.2)	434.78 (217.75, 740.75)	0.8 (0.6, 1.0)	4.0 (2.7, 5.7)	423.92 (209.75, 724.01)	0.0 (0.0, 0.0)	0.1 (0.1, 0.2)	423.00 (211.59, 720.97)
Nigeria	3.6 (2.6, 4.7)	6.9 (5.0, 9.2)	91.30 (22.16,	100.0 (74.1,	186.9 (136.5,	86.92 (20.00,	3.2 (2.3, 4.1)	6.4 (4.7, 8.3)	100.89 (31.41,

Location	Prevalent cases			DALYs			Deaths		
	1990 No. (95% UI)	2019 No. (95% UI)	Relative change % (95% CI)	1990 No. (95% UI)	2019 No. (95% UI)	Relative change % (95% CI)	1990 No. (95% UI)	2019 No. (95% UI)	Relative change % (95% CI)
			188.49)	131.1)	249.1)	179.87)			197.47)
Niue	0.0 (0.0, 0.0)	0.0 (0.0, 0.0)	125.70 (37.56, 247.99)	0.1 (0.1, 0.1)	0.2 (0.1, 0.3)	105.62 (24.00, 218.57)	0.0 (0.0, 0.0)	0.0 (0.0, 0.0)	107.75 (29.78, 216.34)
North Macedonia	0.9 (0.7, 1.2)	3.4 (2.3, 5.2)	276.45 (99.78, 521.54)	24.4 (17.8, 32.8)	83.2 (55.1, 124.9)	240.84 (83.44, 457.69)	0.8 (0.6, 1.1)	3.1 (2.0, 4.6)	276.45 (101.00, 524.24)
Northern Mariana Islands	0.0 (0.0, 0.0)	0.2 (0.2, 0.3)	898.58 (523.60, 1457.01)	0.6 (0.4, 0.8)	4.9 (3.6, 6.5)	709.78 (399.29, 1183.67)	0.0 (0.0, 0.0)	0.2 (0.1, 0.2)	886.45 (522.62, 1426.20)
Norway	0.5 (0.4, 0.6)	2.3 (1.7, 2.9)	346.55 (215.97, 494.78)	9.2 (7.8, 10.7)	26.0 (21.1, 32.1)	183.37 (113.50, 264.87)	0.3 (0.3, 0.4)	1.0 (0.8, 1.2)	197.18 (120.12, 288.86)
Oman	0.1 (0.1, 0.2)	1.2 (0.8, 1.7)	768.06 (400.11, 1372.86)	3.9 (2.4, 5.6)	25.4 (17.2, 35.6)	556.45 (270.09, 1033.52)	0.1 (0.1, 0.2)	0.7 (0.5, 1.0)	526.66 (253.60, 986.94)
Pakistan	4.0 (2.6, 5.5)	15.5 (11.9, 20.0)	289.80 (145.34, 513.51)	117.7 (81.8, 160.7)	463.2 (358.9, 597.7)	293.54 (151.91, 499.16)	3.3 (2.2, 4.6)	12.1 (9.4, 15.7)	269.16 (133.19, 478.69)
Palau	0.0 (0.0, 0.0)	0.1 (0.1, 0.2)	633.41 (325.36, 1134.60)	0.5 (0.3, 0.7)	3.5 (2.5, 4.9)	581.35 (294.29, 1049.47)	0.0 (0.0, 0.0)	0.1 (0.1, 0.1)	581.58 (297.57, 1041.86)
Palestine	0.3 (0.2, 0.4)	1.5 (1.1, 2.0)	427.33 (217.24, 770.27)	7.2 (4.8, 10.3)	33.3 (24.7, 43.8)	364.74 (182.98, 656.88)	0.2 (0.2, 0.4)	1.1 (0.8, 1.4)	339.02 (163.66, 625.60)
Panama	0.2 (0.1, 0.2)	0.8 (0.5, 1.2)	382.16 (159.81, 690.73)	4.5 (3.4, 6.0)	19.3 (12.4, 28.8)	324.16 (131.26, 582.53)	0.1 (0.1, 0.2)	0.6 (0.4, 1.0)	367.18 (144.34, 680.03)
Papua New Guinea	0.2 (0.2, 0.3)	1.1 (0.8, 1.6)	366.45 (175.26, 646.82)	6.5 (4.7, 9.0)	30.2 (21.0, 42.4)	363.38 (173.04, 643.56)	0.2 (0.2, 0.3)	1.0 (0.7, 1.4)	369.39 (176.46, 666.03)
Paraguay	0.1 (0.1, 0.1)	0.5 (0.3, 0.7)	398.50 (159.70,	2.6 (1.9, 3.5)	11.8 (7.3,	360.90 (140.06,	0.1 (0.1, 0.1)	0.4 (0.3, 0.6)	405.74 (152.98,

Location	Prevalent cases			DALYs			Deaths		
	1990<br>No. (95% UI)	2019<br>No. (95% UI)	Relative change<br>% (95% CI)	1990<br>No. (95% UI)	2019<br>No. (95% UI)	Relative change<br>% (95% CI)	1990<br>No. (95% UI)	2019<br>No. (95% UI)	Relative change<br>% (95% CI)
			735.65)		17.7)	671.71)			774.34)
Peru	2.3 (1.8, 2.8)	6.3 (4.3, 8.8)	177.34 (69.03, 310.47)	61.4 (48.8, 75.9)	152.4 (103.2, 212.5)	148.08 (51.93, 264.10)	2.0 (1.6, 2.6)	5.6 (3.8, 8.0)	176.88 (63.02, 322.13)
Philippines	17.5 (13.2, 21.7)	44.0 (34.0, 57.1)	151.72 (72.09, 255.84)	524.8 (395.4, 649.5)	1262.4 (989.2, 1586.1)	140.56 (68.00, 234.68)	14.3 (10.5, 18.2)	36.3 (28.4, 45.7)	153.18 (73.40, 261.48)
Poland	15.8 (13.4, 18.4)	11.4 (8.7, 14.7)	-27.76 (-48.67, -3.87)	414.1 (356.3, 481.3)	266.8 (208.2, 343.3)	-35.56 (-53.63, -14.97)	15.4 (13.1, 18.0)	11.0 (8.6, 14.2)	-28.61 (-48.86, -5.71)
Portugal	1.0 (0.7, 1.4)	10.3 (6.4, 16.1)	893.40 (391.47, 1614.21)	25.5 (18.2, 35.5)	178.9 (120.9, 262.3)	601.98 (294.55, 1060.46)	0.9 (0.6, 1.2)	6.8 (4.6, 10.0)	682.20 (333.76, 1218.45)
Puerto Rico	2.1 (1.5, 2.8)	3.7 (2.4, 5.5)	79.07 (-2.53, 191.84)	51.4 (38.6, 67.3)	75.3 (48.0, 111.9)	46.67 (-20.17, 136.57)	1.9 (1.4, 2.6)	3.1 (1.9, 4.6)	59.01 (-16.28, 163.99)
Qatar	0.1 (0.1, 0.2)	2.9 (1.9, 4.4)	2409.84 (1184.39, 4374.36)	3.1 (2.1, 4.6)	53.2 (33.6, 80.3)	1596.21 (759.87, 2938.52)	0.1 (0.1, 0.1)	1.6 (1.0, 2.5)	1616.04 (760.89, 2956.55)
Republic of Korea	33.8 (26.9, 42.1)	829.1 (640.0, 1066.0)	2351.88 (1603.39, 3309.17)	875.0 (697.3, 1098.1)	7240.8 (5951.9, 8591.4)	727.56 (511.00, 1004.61)	26.4 (21.1, 33.0)	271.2 (220.0, 323.6)	925.54 (649.63, 1272.77)
Republic of Moldova	0.3 (0.2, 0.3)	0.8 (0.5, 1.1)	204.46 (77.04, 388.48)	6.7 (5.0, 9.0)	18.9 (13.1, 27.1)	181.55 (64.92, 344.66)	0.2 (0.2, 0.3)	0.7 (0.5, 1.0)	216.54 (79.51, 419.29)
Romania	1.9 (1.3, 2.6)	7.2 (4.8, 10.5)	279.89 (110.75, 519.90)	50.7 (36.5, 68.3)	174.0 (117.5, 253.2)	243.34 (92.77, 452.81)	1.7 (1.2, 2.3)	6.5 (4.4, 9.4)	275.94 (111.36, 510.64)

Location	Prevalent cases			DALYs			Deaths		
	1990 No. (95% UI)	2019 No. (95% UI)	Relative change % (95% CI)	1990 No. (95% UI)	2019 No. (95% UI)	Relative change % (95% CI)	1990 No. (95% UI)	2019 No. (95% UI)	Relative change % (95% CI)
Russian Federation	10.6 (9.1, 12.4)	33.4 (25.8, 43.4)	214.57 (122.69, 319.38)	287.4 (246.0, 335.4)	853.4 (660.1, 1112.4)	196.91 (109.71, 296.16)	9.2 (7.8, 10.9)	29.1 (22.3, 37.5)	216.76 (123.68, 322.50)
Rwanda	0.3 (0.2, 0.4)	0.7 (0.5, 1.1)	153.81 (32.70, 361.72)	8.8 (5.6, 13.3)	21.7 (13.8, 31.9)	146.88 (27.56, 352.82)	0.2 (0.2, 0.4)	0.6 (0.4, 0.9)	144.29 (29.63, 338.70)
Saint Kitts and Nevis	0.0 (0.0, 0.0)	0.0 (0.0, 0.1)	5.7 (-39.31, 66.77)	0.9 (0.7, 1.2)	0.9 (0.6, 1.3)	0.19 (-41.85, 55.18)	0.0 (0.0, 0.0)	0.0 (0.0, 0.0)	-6.18 (-45.49, 50.48)
Saint Lucia	0.0 (0.0, 0.1)	0.1 (0.1, 0.1)	90.31 (18.62, 187.79)	1.2 (0.9, 1.5)	2.1 (1.5, 2.9)	76.56 (10.22, 165.67)	0.0 (0.0, 0.1)	0.1 (0.1, 0.1)	88.24 (14.02, 190.57)
Saint Vincent and the Grenadines	0.0 (0.0, 0.1)	0.1 (0.0, 0.1)	75.06 (11.25, 161.10)	1.0 (0.8, 1.3)	1.8 (1.3, 2.4)	70.17 (9.09, 150.64)	0.0 (0.0, 0.0)	0.1 (0.0, 0.1)	72.86 (7.29, 166.45)
Samoa	0.1 (0.0, 0.1)	0.2 (0.1, 0.3)	205.73 (88.71, 371.56)	1.8 (1.3, 2.4)	5.4 (3.8, 7.6)	199.81 (81.07, 367.86)	0.1 (0.0, 0.1)	0.2 (0.1, 0.2)	192.19 (82.66, 347.32)
San Marino	0.0 (0.0, 0.0)	0.0 (0.0, 0.0)	577.29 (250.02, 1123.33)	0.0 (0.0, 0.1)	0.2 (0.1, 0.3)	368.31 (96.17, 795.56)	0.0 (0.0, 0.0)	0.0 (0.0, 0.0)	389.35 (88.92, 884.39)
Sao Tome and Principe	0.0 (0.0, 0.0)	0.0 (0.0, 0.0)	220.01 (75.09, 418.46)	0.2 (0.1, 0.2)	0.6 (0.4, 0.9)	228.07 (77.38, 437.66)	0.0 (0.0, 0.0)	0.0 (0.0, 0.0)	193.57 (62.02, 373.91)
Saudi Arabia	1.7 (1.1, 2.3)	16.6 (11.1, 23.4)	876.85 (456.67, 1526.53)	51.5 (33.5, 72.5)	299.5 (199.3, 431.0)	481.38 (221.41, 893.79)	1.6 (1.1, 2.3)	9.3 (6.2, 13.3)	468.28 (215.16, 873.31)
Senegal	0.4 (0.3, 0.5)	1.5 (1.0, 2.0)	273.37 (136.37, 447.18)	11.0 (8.5, 13.6)	40.8 (28.4, 54.9)	269.78 (134.25, 442.05)	0.4 (0.3, 0.4)	1.3 (0.9, 1.8)	265.20 (136.62, 432.78)
Serbia	4.3 (3.0, 6.1)	9.3 (5.8, 14.1)	113.09 (7.79, 265.50)	111.6 (78.2, 154.2)	192.8 (121.5, 298.5)	72.78 (-12.78, 195.62)	3.9 (2.6, 5.4)	7.7 (4.8, 11.8)	99.49 (-0.62, 247.74)

Location	Prevalent cases			DALYs			Deaths		
	1990 No. (95% UI)	2019 No. (95% UI)	Relative change % (95% CI)	1990 No. (95% UI)	2019 No. (95% UI)	Relative change % (95% CI)	1990 No. (95% UI)	2019 No. (95% UI)	Relative change % (95% CI)
Seychelles	0.0 (0.0, 0.0)	0.1 (0.1, 0.2)	366.57 (199.13, 593.98)	0.8 (0.6, 1.1)	3.6 (2.6, 4.8)	332.38 (179.14, 536.71)	0.0 (0.0, 0.0)	0.1 (0.1, 0.2)	317.70 (166.57, 525.16)
Sierra Leone	0.2 (0.1, 0.3)	0.6 (0.4, 0.8)	175.53 (60.59, 347.29)	5.7 (3.9, 7.8)	16.2 (10.9, 22.8)	181.20 (62.27, 360.41)	0.2 (0.1, 0.3)	0.5 (0.3, 0.7)	154.87 (46.88, 313.64)
Singapore	3.2 (2.7, 3.7)	35.2 (25.5, 46.6)	1007.25 (658.19, 1406.36)	67.1 (58.4, 75.8)	305.2 (246.5, 368.0)	355.21 (252.98, 470.23)	2.3 (2.0, 2.7)	12.9 (10.1, 15.8)	454.16 (313.15, 613.85)
Slovakia	1.2 (0.9, 1.6)	2.4 (1.5, 3.6)	99.85 (6.70, 229.62)	31.4 (23.3, 41.9)	52.1 (33.2, 77.9)	65.77 (-10.63, 172.2)	1.1 (0.8, 1.5)	1.9 (1.2, 2.9)	81.33 (-5.26, 202.89)
Slovenia	0.4 (0.3, 0.6)	2.1 (1.3, 3.2)	443.79 (180.07, 881.88)	9.9 (6.5, 14.8)	40.9 (25.5, 61.2)	313.07 (111.96, 653.57)	0.3 (0.2, 0.5)	1.7 (1.1, 2.5)	383.79 (150.42, 775.31)
Solomon Islands	0.1 (0.1, 0.1)	0.4 (0.3, 0.5)	306.07 (162.11, 512.93)	3.0 (2.2, 4.0)	12.3 (9.2, 16.1)	306.15 (162.47, 512.49)	0.1 (0.1, 0.1)	0.3 (0.2, 0.4)	300.49 (155.07, 512.33)
Somalia	0.2 (0.1, 0.4)	0.7 (0.4, 1.3)	225.07 (0.20, 847.60)	6.8 (3.8, 13.0)	22.3 (13.0, 43.5)	228.51 (-11.71, 845.89)	0.2 (0.1, 0.4)	0.6 (0.4, 1.2)	225.03 (-3.13, 841.43)
South Africa	6.2 (4.1, 11.0)	25.9 (21.5, 30.9)	314.71 (157.71, 791.12)	190.3 (126.6, 333.4)	761.6 (635.2, 908.9)	300.32 (150.67, 739.22)	5.0 (3.2, 9.0)	21.5 (18.1, 25.3)	326.29 (162.83, 808.76)
South Sudan	0.1 (0.1, 0.3)	0.3 (0.2, 0.6)	122.23 (-43.13, 585.6)	4.3 (2.4, 8.5)	9.6 (4.9, 18.2)	123.2 (-45.25, 567.35)	0.1 (0.1, 0.3)	0.3 (0.1, 0.5)	117.54 (-45.24, 597.3)
Spain	7.6 (5.4, 10.4)	45.5 (28.7, 69.1)	501.83 (209.95, 915.73)	160.8 (113.8, 222.1)	525.3 (353.1, 754.0)	226.59 (86.46, 434.48)	5.9 (4.1, 8.5)	20.8 (13.8, 30.5)	252.57 (92.32, 506.69)
Sri Lanka	1.5 (1.2, 1.9)	15.6 (10.1,	943.25 (483.81,	40.9 (31.8,	343.9 (222.9,	740.82 (373.89,	1.3 (1.0, 1.7)	12.3 (7.9, 18.2)	833.76 (417.29,

Location	Prevalent cases			DALYs			Deaths		
	1990 No. (95% UI)	2019 No. (95% UI)	Relative change % (95% CI)	1990 No. (95% UI)	2019 No. (95% UI)	Relative change % (95% CI)	1990 No. (95% UI)	2019 No. (95% UI)	Relative change % (95% CI)
		23.1)	1515.75)	51.4)	507.4)	1191.46)			1373.58)
Sudan	1.2 (0.7, 1.9)	5.2 (2.8, 8.3)	327.71 (79.70, 774.69)	32.8 (19.6, 51.1)	131.9 (72.6, 214.7)	302.24 (68.80, 721.87)	1.1 (0.7, 1.8)	4.4 (2.4, 7.3)	292.90 (62.80, 711.26)
Suriname	0.1 (0.1, 0.2)	0.2 (0.2, 0.4)	99.48 (17.20, 210.41)	3.5 (2.6, 4.5)	6.7 (4.6, 9.5)	90.85 (12.90, 195.86)	0.1 (0.1, 0.1)	0.2 (0.2, 0.3)	103.04 (19.43, 217.86)
Sweden	0.4 (0.4, 0.6)	1.4 (1.0, 1.8)	208.25 (100.12, 350.19)	10.7 (8.4, 13.6)	26.8 (20.5, 34.8)	150.54 (70.51, 254.79)	0.4 (0.3, 0.5)	1.1 (0.8, 1.4)	164.88 (77.56, 282.65)
Switzerland	1.1 (0.8, 1.5)	8.3 (5.1, 12.9)	683.52 (288.75, 1258.73)	16.1 (11.3, 22.7)	76.7 (51.3, 109.6)	375.36 (167.53, 688.81)	0.6 (0.4, 0.9)	3.1 (2.1, 4.6)	421.70 (179.99, 812.41)
Syrian Arab Republic	1.2 (0.8, 1.6)	7.3 (4.8, 10.5)	525.75 (251.71, 914.60)	31.8 (22.9, 42.6)	156.0 (104.8, 225.6)	391.04 (177.17, 687.22)	1.0 (0.7, 1.4)	5.2 (3.4, 7.6)	428.99 (194.62, 772.54)
Taiwan (Province of China)	21.9 (18.8, 24.9)	86.3 (62.0, 118.5)	293.29 (158.48, 447.48)	565.0 (491.2, 637.6)	1185.6 (856.5, 1591.6)	109.86 (41.81, 185.82)	16.7 (14.1, 19.2)	43.1 (30.7, 58.7)	158.11 (70.26, 259.20)
Tajikistan	0.0 (0.0, 0.1)	0.8 (0.6, 1.1)	1568.79 (922.43, 2452.06)	1.2 (0.8, 1.5)	23.6 (16.4, 32.5)	1948.20 (1140.32, 3081.20)	0.0 (0.0, 0.0)	0.6 (0.4, 0.9)	1630.02 (906.98, 2683.13)
Thailand	46.3 (36.6, 57.2)	394.5 (268.7, 565.8)	751.29 (407.58, 1165.33)	1330.6 (1051.3, 1623.4)	9068.0 (6158.5, 13000.2)	581.50 (308.53, 909.57)	39.3 (31.2, 48.5)	306.9 (208.3, 436.1)	680.13 (368.60, 1054.20)
Timor-Leste	0.1 (0.0, 0.1)	0.4 (0.2, 0.6)	402.51 (126.00, 893.47)	2.2 (1.3, 3.4)	9.8 (5.1, 15.3)	356.37 (95.92, 821.78)	0.1 (0.0, 0.1)	0.3 (0.2, 0.5)	443.36 (143.45, 973.19)

Location	Prevalent cases			DALYs			Deaths		
	1990 No. (95% UI)	2019 No. (95% UI)	Relative change % (95% CI)	1990 No. (95% UI)	2019 No. (95% UI)	Relative change % (95% CI)	1990 No. (95% UI)	2019 No. (95% UI)	Relative change % (95% CI)
Togo	0.2 (0.1, 0.2)	0.8 (0.5, 1.1)	368.29 (185.74, 619.42)	4.7 (3.5, 6.3)	22.2 (15.4, 30.9)	369.45 (185.26, 623.25)	0.1 (0.1, 0.2)	0.6 (0.5, 0.9)	361.15 (179.67, 612.05)
Tokelau	0.0 (0.0, 0.0)	0.0 (0.0, 0.0)	105.03 (12.32, 251.31)	0.0 (0.0, 0.1)	0.1 (0.0, 0.1)	97.66 (8.38, 237.14)	0.0 (0.0, 0.0)	0.0 (0.0, 0.0)	90.23 (5.18, 224.42)
Tonga	0.2 (0.1, 0.2)	0.5 (0.3, 0.7)	177.24 (58.64, 364.45)	4.8 (3.1, 6.6)	13.0 (8.7, 18.4)	171.89 (54.36, 354.75)	0.1 (0.1, 0.2)	0.4 (0.3, 0.6)	174.67 (56.76, 360.77)
Trinidad and Tobago	0.5 (0.4, 0.7)	0.8 (0.5, 1.3)	55.57 (-14.95, 148.15)	14.9 (11.5, 19.1)	21.6 (14.0, 31.8)	44.89 (-19.07, 126.85)	0.5 (0.4, 0.7)	0.8 (0.5, 1.1)	55.61 (-15.35, 153.62)
Tunisia	0.5 (0.3, 0.7)	3.8 (2.3, 5.9)	715.74 (295.79, 1344.36)	12.1 (8.4, 17.0)	73.5 (43.5, 114.3)	507.02 (192.21, 964.86)	0.4 (0.3, 0.6)	2.6 (1.5, 4.0)	535.56 (204.75, 1042.62)
Turkey	8.4 (6.4, 10.7)	40.6 (29.6, 55.3)	382.52 (207.10, 614.12)	224.9 (170.8, 288.1)	749.9 (552.0, 1005.3)	233.49 (116.37, 389.47)	7.4 (5.6, 9.4)	28.1 (20.4, 38.3)	278.46 (140.27, 461.06)
Turkmenistan	0.0 (0.0, 0.1)	1.6 (1.1, 2.2)	3085.31 (1784.60, 4763.40)	1.2 (0.9, 1.5)	44.6 (30.7, 63.3)	3681.28 (2145.05, 5751.03)	0.0 (0.0, 0.1)	1.2 (0.8, 1.8)	3194.18 (1804.84, 5132.78)
Tuvalu	0.0 (0.0, 0.0)	0.0 (0.0, 0.0)	149.54 (41.41, 310.70)	0.3 (0.2, 0.4)	0.7 (0.5, 1.1)	145.29 (40.52, 299.70)	0.0 (0.0, 0.0)	0.0 (0.0, 0.0)	141.77 (38.99, 294.32)
Uganda	0.6 (0.4, 0.9)	2.9 (1.9, 4.1)	353.45 (156.46, 660.21)	19.2 (13.3, 26.8)	86.9 (58.0, 125.1)	353.57 (153.61, 656.53)	0.6 (0.4, 0.8)	2.3 (1.6, 3.4)	311.40 (129.44, 594.79)
Ukraine	2.6 (2.2, 3.0)	12.1 (9.7, 15.1)	365.29 (244.49, 503.98)	63.3 (53.7, 74.4)	292.5 (228.2, 369.3)	362.00 (237.79, 506.47)	2.4 (2.0, 2.8)	10.2 (8.1, 12.8)	335.24 (218.10, 474.18)
United Arab Emirates	0.1 (0.1, 0.3)	5.2 (2.2, 11.7)	3626.91 (61.86,	4.2 (2.2, 7.8)	150.5 (63.1,	3446.26 (-25.7,	0.1 (0.1, 0.2)	3.8 (1.5, 8.8)	3457.38 (-

Location	Prevalent cases			DALYs			Deaths		
	1990 No. (95% UI)	2019 No. (95% UI)	Relative change % (95% CI)	1990 No. (95% UI)	2019 No. (95% UI)	Relative change % (95% CI)	1990 No. (95% UI)	2019 No. (95% UI)	Relative change % (95% CI)
			11214.96)		341.5)	10583.03)			186.73, 11239.06)
United Kingdom	8.4 (7.1, 9.9)	83.1 (62.5, 106.4)	893.21 (597.96, 1229.28)	134.7 (112.9, 160.3)	801.3 (666.3, 957.4)	494.65 (354.14, 661.27)	5.1 (4.2, 6.1)	33.2 (27.3, 39.9)	549.98 (390.91, 740.49)
United Republic of Tanzania	0.3 (0.2, 0.4)	1.3 (0.9, 1.9)	343.95 (156.99, 617.15)	8.7 (6.3, 11.7)	38.2 (25.9, 54.9)	339.26 (154.68, 603.61)	0.2 (0.2, 0.4)	1.1 (0.8, 1.6)	343.14 (151.23, 640.38)
United States of America	35.3 (30.9, 40.4)	297.3 (224.7, 388.7)	742.47 (495.36, 1022.03)	613.2 (535.5, 699.3)	3657.3 (2949.9, 4509.6)	496.40 (355.06, 657.18)	21.6 (18.8, 24.9)	137.6 (111.2, 168.3)	535.85 (384.22, 705.71)
United States Virgin Islands	0.0 (0.0, 0.0)	0.1 (0.1, 0.1)	203.31 (73.09, 394.31)	0.7 (0.5, 0.9)	1.8 (1.2, 2.6)	166.63 (54.35, 328.07)	0.0 (0.0, 0.0)	0.1 (0.0, 0.1)	224.68 (84.93, 431.93)
Uruguay	0.1 (0.0, 0.1)	0.5 (0.3, 0.8)	678.55 (302.08, 1246.77)	1.7 (1.2, 2.4)	11.8 (8.3, 16.5)	605.78 (314.88, 1059.66)	0.1 (0.0, 0.1)	0.5 (0.3, 0.7)	646.49 (323.16, 1175.84)
Uzbekistan	0.2 (0.1, 0.3)	8.9 (6.3, 12.4)	4434.74 (2696.87, 6799.01)	4.7 (3.6, 6.1)	249.5 (179.0, 341.5)	5185.53 (3240.67, 7791.58)	0.2 (0.1, 0.2)	7.1 (4.9, 10.1)	4470.81 (2620.83, 7074.95)
Vanuatu	0.1 (0.0, 0.1)	0.4 (0.2, 0.5)	470.14 (191.73, 957.53)	1.8 (1.2, 2.8)	10.3 (6.5, 15.3)	466.40 (188.31, 951.47)	0.1 (0.0, 0.1)	0.3 (0.2, 0.5)	464.67 (186.18, 958.29)
Venezuela (Bolivarian Republic of)	1.7 (1.2, 2.3)	4.1 (2.6, 6.4)	146.05 (25.25, 316.20)	46.3 (34.2, 61.8)	101.3 (64.7, 157.6)	118.67 (12.96, 264.52)	1.5 (1.0, 2.0)	3.6 (2.2, 5.6)	148.28 (24.45, 326.95)
Viet Nam	6.0 (4.1, 8.2)	23.2 (15.7, 32.7)	288.64 (123.60, 537.95)	159.5 (107.7, 217.7)	554.2 (376.7, 785.8)	247.55 (99.47, 469.75)	5.6 (3.8, 7.7)	19.8 (13.4, 28.1)	253.78 (102.29, 482.09)

Location	Prevalent cases			DALYs			Deaths		
	1990 No. (95% UI)	2019 No. (95% UI)	Relative change % (95% CI)	1990 No. (95% UI)	2019 No. (95% UI)	Relative change % (95% CI)	1990 No. (95% UI)	2019 No. (95% UI)	Relative change % (95% CI)
Yemen	0.3 (0.2, 0.5)	1.8 (1.1, 2.7)	439.90 (161.89, 1046.95)	9.0 (5.3, 14.9)	47.1 (30.1, 71.2)	420.98 (152.89, 1007.89)	0.3 (0.2, 0.5)	1.5 (1.0, 2.3)	420.71 (152.39, 988.66)
Zambia	0.1 (0.1, 0.2)	0.6 (0.4, 0.8)	301.43 (124.61, 601.67)	4.2 (2.8, 6.3)	16.8 (11.3, 23.3)	299.68 (125.45, 591.80)	0.1 (0.1, 0.2)	0.5 (0.3, 0.7)	286.31 (113.87, 591.14)
Zimbabwe	1.8 (1.2, 3.1)	6.8 (4.5, 10.5)	271.56 (80.13, 670.55)	52.4 (35.7, 89.7)	203.7 (134.6, 318.2)	288.63 (85.82, 710.19)	1.5 (1.0, 2.6)	5.5 (3.7, 8.5)	259.05 (76.13, 644.29)

CI confidence interval, DALY disability-adjusted life year, T2DM type 2 diabetes mellitus, HBV hepatitis B virus, SDI socio-demographic index, UI uncertainty interval.

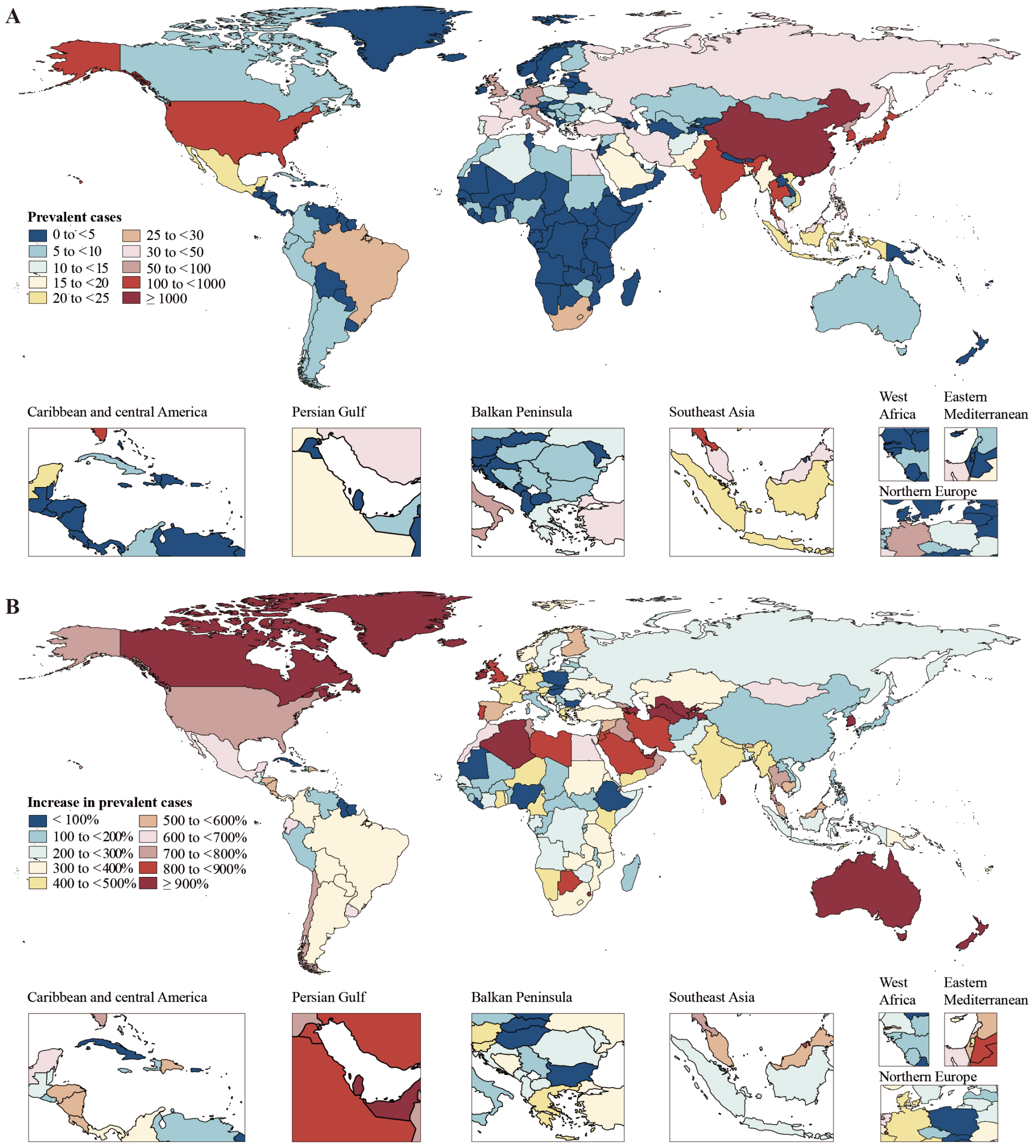

Figure S2. Prevalent cases (A) of liver cancer due to HBV–T2DM comorbidity in the 204 countries and territories in 2019. Relative changes in the number of prevalent cases (B) of liver cancer due to HBV–T2DM comorbidity in the 204 countries and territories between 1990 and 2019. HBV hepatitis B virus, T2DM type 2 diabetes mellitus

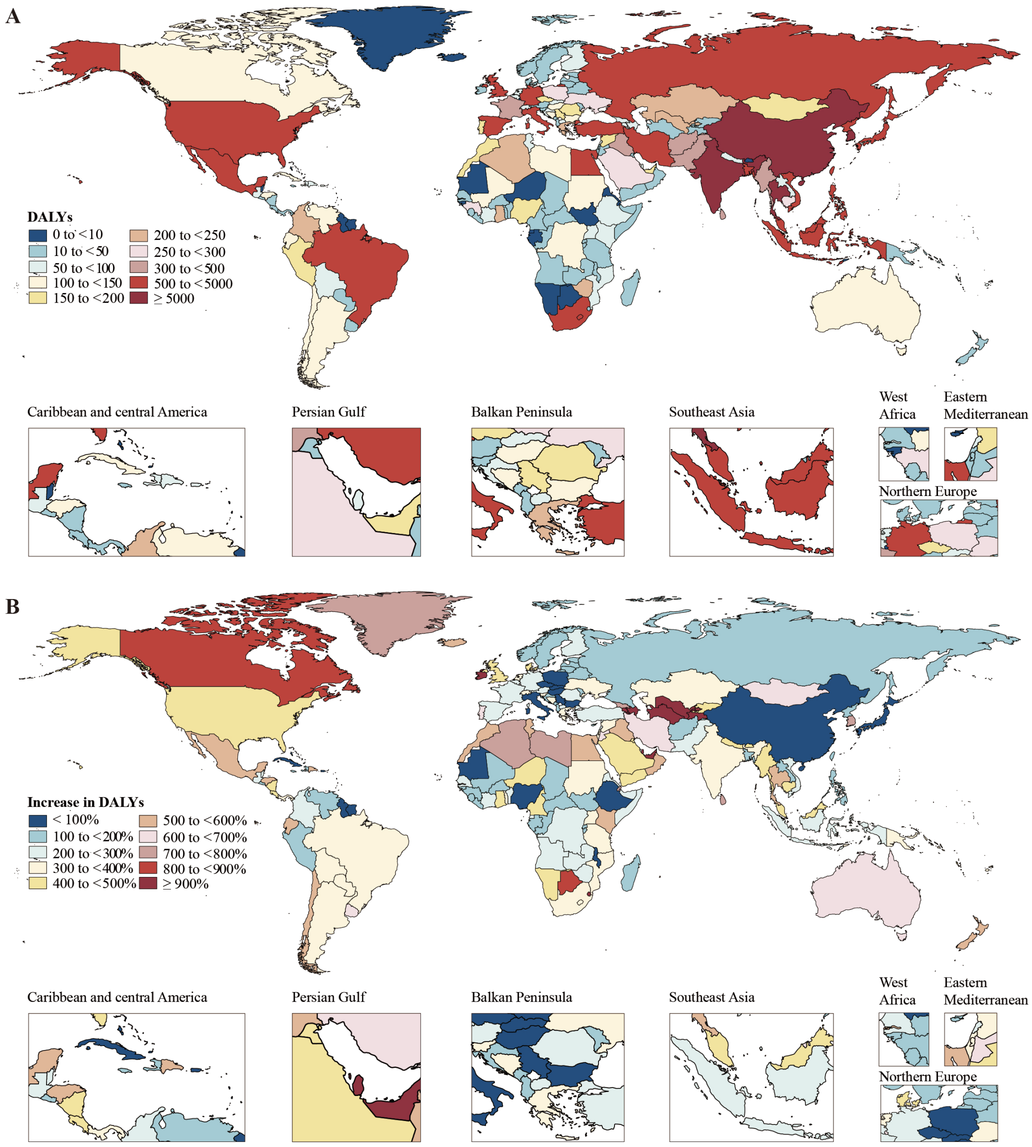

Figure S3. DALYs (A) of liver cancer due to HBV–T2DM comorbidity in the 204 countries and territories in 2019. Relative changes in DALYs (B) of liver cancer due to HBV–T2DM comorbidity in the 204 countries and territories between 1990 and 2019. DALY disability-adjusted life year, HBV hepatitis B virus, T2DM type 2 diabetes mellitus

Table S5. Age-standardized mortality rates (per 10,000,000 individuals) for liver cancer due to HBV–T2DM comorbidity in 1990 and 2019 and the EAPCs from 1990 to 2019, stratified by SDI and region

Location	Age-standardized mortality rate		
	1990	2019	EAPC
	No. (95% UI)	No. (95% UI)	% (95% CI)
**Global**	7.5 (6.4, 8.7)	6.1 (5.1, 7.1)	-2.05 (-2.59, -1.50)
**SDI**			
Low SDI	2.2 (1.8, 2.7)	1.6 (1.2, 1.9)	0.59 (0.45, 0.72)
Low-middle SDI	4.4 (3.8, 5.0)	3.7 (3.1, 4.2)	-1.12 (-1.59, -0.65)
Middle SDI	14.9 (12.5, 17.8)	10.2 (8.5, 12.3)	-2.8 (-3.46, -2.15)
High-middle SDI	8.5 (7.1, 10.1)	6.4 (5.3, 7.6)	-3.51 (-4.15, -2.87)
High SDI	1.8 (1.6, 2.1)	5.0 (4.2, 5.9)	2.01 (1.57, 2.45)
**Regions**			
High-income Asia Pacific	3.6 (3.1, 4.1)	10.1 (8.6, 11.8)	1.11 (0.33, 1.89)
Central Asia	1.2 (0.8, 1.6)	4.6 (3.3, 6.3)	4.81 (4.16, 5.45)
East Asia	27.3 (22.7, 32.8)	16.8 (13.8, 20.4)	-4.19 (-4.89, -3.49)
South Asia	1.7 (1.3, 2.0)	2.1 (1.8, 2.6)	1.24 (1.10, 1.39)
Southeast Asia	4.0 (3.2, 4.8)	5.6 (4.2, 7.5)	1.06 (0.90, 1.22)
Australasia	0.3 (0.2, 0.4)	1.4 (1.0, 1.9)	3.94 (3.58, 4.29)
Caribbean	4.2 (3.1, 5.5)	3.2 (2.2, 4.4)	-1.23 (-2.11, -0.33)
Central Europe	2.2 (1.7, 2.8)	3.0 (2.2, 4.1)	-0.51 (-0.90, -0.12)
Eastern Europe	0.4 (0.3, 0.5)	1.3 (1.1, 1.7)	3.15 (2.79, 3.51)
Western Europe	0.7 (0.5, 0.9)	2.3 (1.7, 3.0)	2.03 (1.90, 2.16)
Andean Latin America	2.8 (2.2, 3.6)	2.4 (1.8, 3.2)	-0.68 (-1.07, -0.29)
Central Latin America	1.8 (1.3, 2.4)	1.9 (1.4, 2.6)	0.22 (-0.16, 0.60)
Southern Latin America	0.4 (0.3, 0.5)	1.1 (0.8, 1.6)	3.55 (3.39, 3.72)
Tropical Latin America	0.8 (0.7, 1.0)	1.1 (0.9, 1.2)	1.10 (0.88, 1.32)
North Africa and Middle East	2.9 (2.3, 3.7)	3.7 (2.7, 4.8)	1.85 (1.67, 2.03)
High-income North America	0.5 (0.5, 0.6)	2.4 (1.9, 2.9)	4.98 (4.54, 5.43)
Oceania	5.9 (4.5, 7.4)	5.8 (4.4, 7.4)	1.30 (1.23, 1.36)
Central Sub-Saharan Africa	1.1 (0.8, 1.5)	0.6 (0.4, 0.9)	0.33 (0.17, 0.48)
Eastern Sub-Saharan Africa	0.9 (0.7, 1.3)	0.6 (0.4, 0.8)	0.64 (0.43, 0.86)
Southern Sub-Saharan Africa	3.9 (2.5, 6.8)	4.8 (4.0, 5.7)	0.99 (0.21, 1.77)
Western Sub-Saharan Africa	2.7 (2.1, 3.3)	1.6 (1.3, 2.1)	0.73 (0.60, 0.86)

CI confidence interval, T2DM type 2 diabetes mellitus, EAPC estimated annual percentage change, HBV hepatitis B virus, SDI sociodemographic index, UI uncertainty interval.

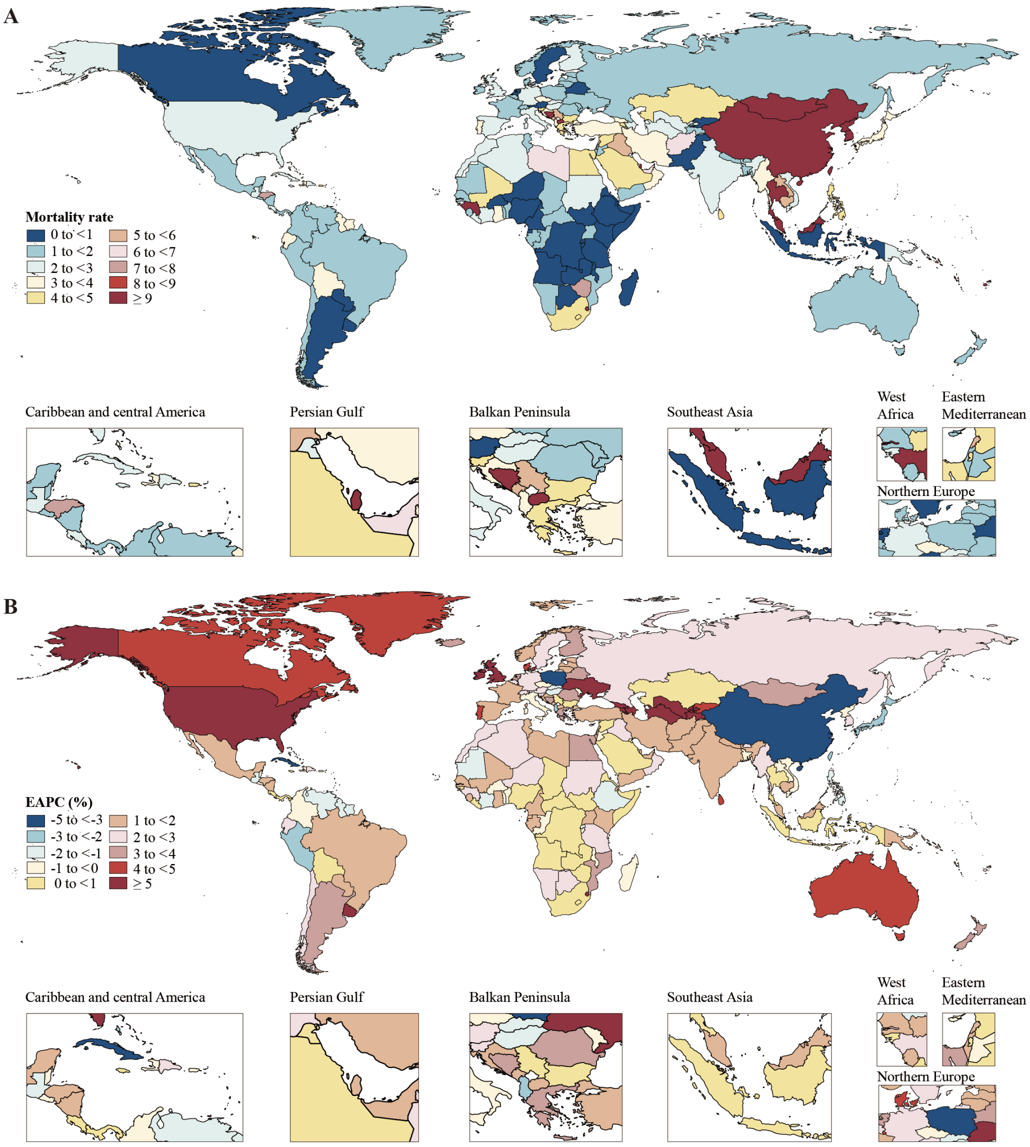

Figure S4. The age-standardized mortality (A) rates (per 10,000,000 individuals) of liver cancer due to HBV–T2DM comorbidity in 204 countries and territories in 2019; EAPCs in the age-standardized mortality (B) rates of liver cancer due to HBV–T2DM comorbidity in 204 countries and territories from 1990 to 2019. EAPC estimated annual percentage change, HBV hepatitis B virus, T2DM type 2 diabetes mellitus

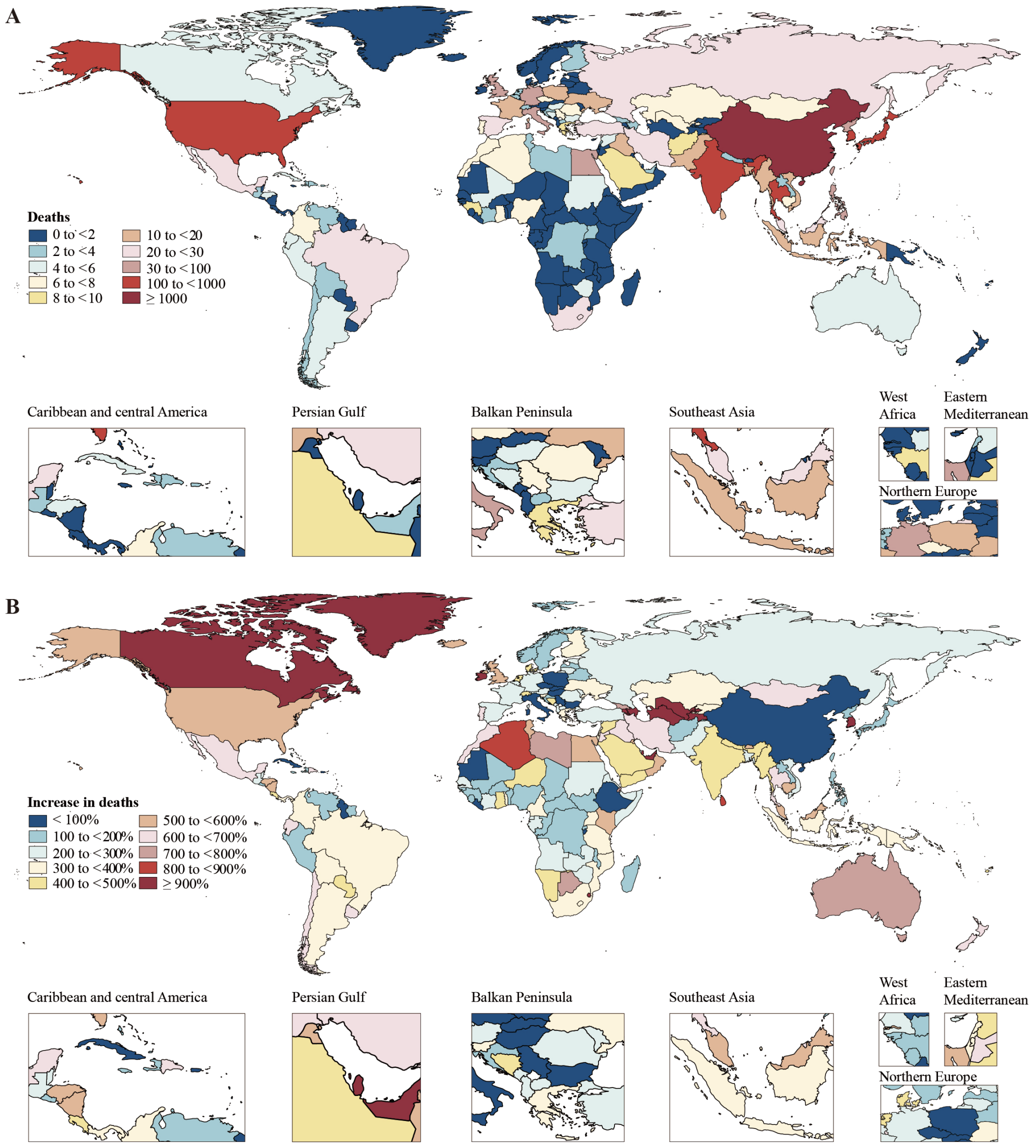

Figure S5. Deaths (A) of liver cancer due to HBV–T2DM comorbidity in the 204 countries and territories in 2019. Relative changes in deaths (B) of liver cancer due to HBV–T2DM comorbidity in the 204 countries and territories between 1990 and 2019. HBV hepatitis B virus, T2DM type 2 diabetes mellitus

Table S6. Age-standardized prevalence, DALY, and mortality rates (per 10,000,000 individuals) for liver cancer due to HBV–T2DM comorbidity in 1990 and 2019 among men and the EAPCs from 1990 to 2019, stratified by SDI and region

Location	Age-standardized prevalence rate among men			Age-standardized DALY rate among men			Age-standardized mortality rate		
	1990 No. (95% UI)	2019 No. (95% UI)	EAPC % (95% CI)	1990 No. (95% UI)	2019 No. (95% UI)	EAPC % (95% CI)	1990 No. (95% UI)	2019 No. (95% UI)	EAPC % (95% CI)
**Global**	15.5 (12.8, 18.4)	17.2 (14.4, 20.2)	-0.71 (-1.25, -0.17)	424.4 (353.3, 504.8)	312.6 (261.7, 370.4)	-2.26 (-2.86, -1.66)	13.1 (10.8, 15.5)	10.5 (8.8, 12.4)	-1.95 (-2.52, -1.37)
**SDI**									
Low SDI	3.5 (2.8, 4.3)	4.7 (3.7, 5.8)	0.72 (0.58, 0.86)	96.1 (76.8, 116.6)	128.4 (101.4, 157.4)	0.67 (0.52, 0.81)	3.5 (2.8, 4.3)	4.6 (3.6, 5.6)	0.63 (0.47, 0.80)
Low-middle SDI	7.5 (6.3, 8.9)	8.2 (7.0, 9.6)	-0.93 (-1.46, -0.39)	214.5 (179.8, 254.1)	213.0 (180.2, 246.3)	-1.26 (-1.80, -0.73)	6.8 (5.7, 8.0)	7.3 (6.2, 8.5)	-0.94 (-1.44, -0.43)
Middle SDI	27.6 (22.1, 33.5)	25.5 (20.6, 31.4)	-1.55 (-2.30, -0.80)	772.9 (627.2, 937.8)	502.7 (405.6, 611.5)	-2.76 (-3.47, -2.05)	24.2 (19.6, 29.3)	17.0 (13.8, 20.7)	-2.47 (-3.15, -1.78)
High-middle SDI	18.9 (15.2, 22.8)	16.1 (12.6, 19.7)	-1.98 (-2.66, -1.29)	516.5 (417.2, 626.8)	275.7 (222.5, 341.5)	-3.69 (-4.39, -3.00)	16.1 (13.1, 19.4)	9.0 (7.3, 11.1)	-3.50 (-4.18, -2.82)
High SDI	5.5 (4.8, 6.1)	16.9 (14.1, 19.9)	4.30 (3.79, 4.82)	110.0 (97.2, 124.4)	169.5 (145.9, 195.6)	1.52 (1.02, 2.02)	3.5 (3.1, 4.0)	6.1 (5.1, 7.1)	1.95 (1.48, 2.42)
**Regions**									
High-income Asia Pacific	13.4 (11.8, 15.2)	38.8 (31.3, 47.4)	3.80 (3.17, 4.44)	244.1 (213.9, 278.8)	330.3 (281.2, 382.3)	0.84 (-0.01, 1.71)	7.9 (6.8, 9.0)	11.6 (9.8, 13.4)	1.14 (0.33, 1.95)
Central Asia	2 (1.5, 2.7)	8.4 (6.1, 11.3)	4.27 (3.67, 4.87)	54.7 (40.5, 73.3)	226.1 (163.4, 301.8)	4.26 (3.63, 4.90)	1.9 (1.4, 2.6)	7.8 (5.6, 10.6)	4.29 (3.69, 4.88)
East Asia	52.1 (41.8, 63.4)	38.5 (30.3, 47.6)	-2.44 (-3.21, -1.66)	1448.9 (1170, 1772.6)	710.9 (558.6, 885.1)	-3.89 (-4.65, -3.12)	45.6 (37.0, 55.5)	23.2 (18.5, 28.7)	-3.71 (-4.44, -2.98)

Location	Age-standardized prevalence rate among men			Age-standardized DALY rate among men			Age-standardized mortality rate		
	1990	2019	EAPC	1990	2019	EAPC	1990	2019	EAPC
	No. (95% UI)	No. (95% UI)	% (95% CI)	No. (95% UI)	No. (95% UI)	% (95% CI)	No. (95% UI)	No. (95% UI)	% (95% CI)
South Asia	2.7 (2.1, 3.3)	4.3 (3.4, 5.3)	1.41 (1.29, 1.53)	74.7 (59.8, 90.5)	113.3 (91.3, 139.0)	1.23 (1.11, 1.36)	2.6 (2.0, 3.2)	4.0 (3.2, 5.0)	1.37 (1.21, 1.52)
Southeast Asia	6.8 (5.4, 8.2)	11.3 (8.4, 14.8)	1.40 (1.22, 1.59)	189.2 (151.6, 228.9)	274.8 (205.5, 356.5)	0.88 (0.70, 1.07)	6.2 (4.9, 7.6)	9.8 (7.2, 12.9)	1.22 (1.03, 1.41)
Australasia	0.8 (0.6, 1.0)	3.4 (2.3, 4.8)	5.29 (4.72, 5.86)	15.9 (11.9, 20.7)	46.6 (34.2, 63.0)	3.81 (3.52, 4.09)	0.5 (0.4, 0.7)	1.6 (1.2, 2.2)	3.87 (3.58, 4.17)
Caribbean	6.2 (4.6, 8.1)	5.3 (3.6, 7.3)	-0.57 (-1.41, 0.27)	161.8 (122.1, 209.9)	131.2 (91.6, 183.3)	-0.80 (-1.69, 0.09)	5.7 (4.2, 7.7)	4.6 (3.2, 6.5)	-0.74 (-1.62, 0.14)
Central Europe	3.6 (2.9, 4.6)	3.9 (2.8, 5.4)	0.49 (0.17, 0.81)	95.2 (75.9, 118.3)	89.6 (66.0, 122.2)	-0.07 (-0.41, 0.26)	3.4 (2.7, 4.3)	3.3 (2.4, 4.6)	0.07 (-0.27, 0.41)
Eastern Europe	0.8 (0.7, 0.9)	1.9 (1.5, 2.5)	3.48 (3.13, 3.84)	20.1 (17.3, 23.5)	48.0 (36.6, 62.3)	3.31 (2.90, 3.73)	0.7 (0.6, 0.8)	1.6 (1.2, 2.1)	3.25 (2.86, 3.63)
Western Europe	1.8 (1.4, 2.3)	6.0 (4.5, 8.0)	4.10 (3.76, 4.43)	33.7 (26.0, 42.9)	62.6 (47.3, 82.1)	1.90 (1.71, 2.08)	1.2 (0.9, 1.6)	2.3 (1.7, 3.1)	1.96 (1.77, 2.14)
Andean Latin America	3.3 (2.6, 4.0)	3.5 (2.5, 4.6)	-0.19 (-0.61, 0.23)	85.9 (67.8, 105.2)	84.1 (61.5, 110.3)	-0.49 (-0.93, -0.06)	3.2 (2.4, 3.9)	3.3 (2.3, 4.4)	-0.22 (-0.66, 0.22)
Central Latin America	2.3 (1.7, 3.0)	3.0 (2.2, 4.2)	1.09 (0.65, 1.52)	61.7 (46.6, 80.4)	74.0 (54.7, 101.3)	0.67 (0.21, 1.12)	2.1 (1.6, 2.8)	2.7 (1.9, 3.8)	0.95 (0.53, 1.37)
Southern Latin America	0.6 (0.4, 0.8)	1.9 (1.2, 2.8)	4.32 (4.17, 4.47)	15.5 (10.8, 21.5)	40.9 (29.0, 57.0)	3.83 (3.69, 3.98)	0.6 (0.4, 0.8)	1.6 (1.1, 2.3)	4.08 (3.93, 4.23)
Tropical Latin America	1.4 (1.2, 1.6)	1.9 (1.6, 2.2)	1.68 (1.42, 1.94)	37.6 (32.5, 42.8)	46.7 (40.0, 54.4)	1.36 (1.11, 1.61)	1.2 (1.1, 1.4)	1.7 (1.4, 2.0)	1.69 (1.44, 1.95)

Location	Age-standardized prevalence rate among men			Age-standardized DALY rate among men			Age-standardized mortality rate		
	1990 No. (95% UI)	2019 No. (95% UI)	EAPC % (95% CI)	1990 No. (95% UI)	2019 No. (95% UI)	EAPC % (95% CI)	1990 No. (95% UI)	2019 No. (95% UI)	EAPC % (95% CI)
North Africa and Middle East	4.5 (3.5, 5.6)	7.9 (5.8, 10.3)	2.30 (2.10, 2.50)	120.6 (95.6, 150.6)	193.4 (142.6, 254.1)	1.95 (1.77, 2.13)	4.3 (3.4, 5.5)	7 (5.2, 9.2)	2.00 (1.82, 2.17)
High-income North America	1.6 (1.4, 1.9)	7.0 (5.2, 9.4)	6.23 (5.73, 6.73)	27.4 (23.7, 31.5)	80.1 (63.1, 99.0)	4.86 (4.45, 5.28)	0.9 (0.8, 1.1)	2.8 (2.2, 3.5)	5.04 (4.61, 5.47)
Oceania	9.8 (7.4, 12.3)	14.0 (10.8, 17.8)	1.42 (1.35, 1.49)	265.8 (202.2, 332.6)	369.2 (287.4, 469.6)	1.32 (1.24, 1.39)	9.6 (7.1, 12.1)	13.6 (10.4, 17.3)	1.38 (1.32, 1.44)
Central Sub-Saharan Africa	1.9 (1.3, 2.6)	2.2 (1.5, 3.3)	0.40 (0.20, 0.61)	53.7 (38.1, 72.0)	62.9 (42.5, 92.5)	0.32 (0.12, 0.52)	1.8 (1.2, 2.5)	2.1 (1.4, 3.1)	0.30 (0.07, 0.52)
Eastern Sub-Saharan Africa	1.3 (1.0, 2.0)	2.0 (1.4, 2.8)	1.01 (0.74, 1.27)	37.6 (27.2, 56.4)	54.5 (38.0, 75.8)	0.96 (0.67, 1.24)	1.3 (0.9, 2.0)	1.9 (1.3, 2.6)	0.99 (0.70, 1.28)
Southern Sub-Saharan Africa	6.2 (3.7, 12.3)	11.1 (9.2, 13.3)	1.19 (0.45, 1.94)	177.4 (106.2, 349.1)	317.3 (264.6, 376.5)	1.21 (0.33, 2.09)	5.6 (3.3, 11.1)	10 (8.3, 11.9)	1.24 (0.34, 2.15)
Western Sub-Saharan Africa	4.1 (3.2, 5.0)	5.6 (4.4, 7.1)	0.96 (0.88, 1.05)	111.1 (87.9, 137.6)	151.1 (115.2, 188.5)	0.89 (0.81, 0.98)	4.1 (3.3, 5.1)	5.6 (4.3, 7.0)	0.96 (0.84, 1.08)

CI confidence interval, DALY disability-adjusted life year, T2DM type 2 diabetes mellitus, EAPC estimated annual percentage change, HBV hepatitis B virus, SDI socio-demographic index, UI uncertainty interval.

Table S7. Age-standardized prevalence, DALY, and mortality rates (per 10,000,000 individuals) for liver cancer due to HBV–T2DM comorbidity in 1990 and 2019 among women and the EAPCs from 1990 to 2019, stratified by SDI and region

Location	Age-standardized prevalence rate among women			Age-standardized DALY rate among women			Age-standardized mortality rate among women		
	1990<br>No. (95% UI)	2019<br>No. (95% UI)	EAPC<br>% (95% CI)	1990<br>No. (95% UI)	2019<br>No. (95% UI)	EAPC<br>% (95% CI)	1990<br>No. (95% UI)	2019<br>No. (95% UI)	EAPC<br>% (95% CI)
**Global**	3.1 (2.5, 3.7)	2.7 (2.2, 3.3)	-1.18 (-1.53, -0.82)	82.4 (66.2, 100.0)	51.4 (42.1, 62.4)	-2.53 (-2.97, -2.08)	2.7 (2.2, 3.3)	1.8 (1.5, 2.2)	-2.10 (-2.50, -1.70)
**SDI**									
Low SDI	1.2 (0.9, 1.5)	1.5 (1.2, 1.9)	0.74 (0.67, 0.81)	33.4 (25.1, 42.6)	43.1 (33.5, 54.9)	0.69 (0.62, 0.76)	1.1 (0.8, 1.4)	1.4 (1.1, 1.8)	0.63 (0.54, 0.71)
Low-middle SDI	2.2 (1.8, 2.7)	2.0 (1.6, 2.5)	-1.04 (-1.39, -0.68)	60.8 (49.5, 73.8)	52.8 (42.1, 65.6)	-1.27 (-1.64, -0.90)	2.0 (1.7, 2.5)	1.8 (1.4, 2.3)	-1.10 (-1.45, -0.76)
Middle SDI	6.5 (5.1, 8.1)	3.9 (3.1, 4.8)	-2.81 (-3.38, -2.23)	178.0 (141.3, 222.8)	79.0 (62.8, 97.9)	-3.90 (-4.47, -3.32)	6.0 (4.7, 7.5)	3.0 (2.4, 3.7)	-3.42 (-3.94, -2.89)
High-middle SDI	2.9 (2.3, 3.5)	2.0 (1.6, 2.5)	-2.23 (-2.65, -1.82)	77.0 (61.1, 94.7)	35.8 (29.0, 44.4)	-3.73 (-4.22, -3.24)	2.5 (2.0, 3.1)	1.3 (1.0, 1.6)	-3.38 (-3.80, -2.94)
High SDI	0.9 (0.8, 1.1)	2.7 (2.2, 3.4)	4.00 (3.56, 4.43)	16.4 (13.7, 19.7)	25.7 (20.7, 31.6)	1.60 (1.28, 1.93)	0.6 (0.5, 0.7)	1.0 (0.8, 1.2)	2.08 (1.73, 2.43)
**Regions**									
High-income Asia Pacific	1.7 (1.5, 2.0)	4.6 (3.6, 5.9)	2.76 (2.21, 3.31)	25.8 (21.3, 31.2)	32.3 (25.8, 39.8)	-0.00 (-0.73, 0.73)	0.9 (0.8, 1.1)	1.3 (1.0, 1.6)	0.31 (-0.39, 1.02)
Central Asia	0.6 (0.4, 0.9)	3.1 (2.2, 4.3)	5.86 (5.13, 6.60)	16.5 (11.8, 22.8)	84.2 (59.1, 115.4)	5.80 (5.05, 6.55)	0.6 (0.4, 0.8)	2.9 (2.0, 4.1)	6.01 (5.27, 6.76)
East Asia	10.7 (8.5, 13.6)	4.4 (3.4, 5.6)	-4.25 (-4.82, -3.68)	294.5 (229.4, 371.3)	84.8 (65.7, 108.4)	-5.55 (-6.17, -4.92)	9.8 (7.7, 12.4)	3.1 (2.4, 3.9)	-5.11 (-5.67, -4.55)

Location	Age-standardized prevalence rate among women			Age-standardized DALY rate among women			Age-standardized mortality rate among women		
	1990 No. (95% UI)	2019 No. (95% UI)	EAPC % (95% CI)	1990 No. (95% UI)	2019 No. (95% UI)	EAPC % (95% CI)	1990 No. (95% UI)	2019 No. (95% UI)	EAPC % (95% CI)
South Asia	0.8 (0.6, 1.0)	1.3 (1.0, 1.7)	1.58 (1.47, 1.69)	22.5 (17.8, 28.4)	35.8 (26.8, 47.3)	1.45 (1.34, 1.56)	0.7 (0.6, 1.0)	1.2 (0.9, 1.6)	1.46 (1.32, 1.59)
Southeast Asia	2.0 (1.6, 2.6)	3.1 (2.2, 4.2)	1.14 (1.03, 1.26)	55.5 (43.5, 69.1)	67.7 (49.8, 90.7)	0.38 (0.24, 0.51)	1.9 (1.5, 2.4)	2.6 (1.9, 3.6)	0.76 (0.64, 0.87)
Australasia	0.1 (0.1, 0.2)	0.6 (0.4, 0.8)	4.57 (4.08, 5.06)	3.1 (2.3, 4.1)	9.6 (7.1, 13.0)	3.82 (3.43, 4.21)	0.1 (0.1, 0.1)	0.3 (0.2, 0.5)	4.03 (3.61, 4.45)
Caribbean	3.0 (2.2, 4.0)	1.8 (1.2, 2.5)	-1.91 (-2.74, -1.06)	78.0 (56.8, 104.9)	45.8 (31.5, 64.4)	-1.97 (-2.82, -1.10)	2.8 (2.0, 3.8)	1.5 (1.0, 2.2)	-2.14 (-3.02, -1.25)
Central Europe	1.4 (1.1, 1.7)	0.9 (0.7, 1.3)	-1.17 (-1.57, -0.77)	35.9 (28.7, 44.2)	21.8 (15.7, 30.0)	-1.57 (-1.98, -1.16)	1.3 (1.0, 1.7)	0.8 (0.5, 1.1)	-1.63 (-2.08, -1.18)
Eastern Europe	0.3 (0.2, 0.3)	0.5 (0.4, 0.6)	2.71 (2.42, 3.01)	7.0 (5.9, 8.2)	12.8 (10.0, 15.9)	2.51 (2.17, 2.86)	0.2 (0.2, 0.3)	0.4 (0.3, 0.5)	2.57 (2.25, 2.90)
Western Europe	0.5 (0.4, 0.6)	1.3 (1.0, 1.8)	3.71 (3.54, 3.88)	9.0 (7.1, 11.3)	15.7 (12.0, 20.3)	1.96 (1.91, 2.01)	0.3 (0.3, 0.4)	0.6 (0.4, 0.8)	2.03 (1.99, 2.08)
Andean Latin America	2.5 (1.9, 3.3)	2.2 (1.6, 3.0)	-1.22 (-1.58, -0.87)	66.9 (51.7, 86.4)	54.4 (39.3, 73.6)	-1.45 (-1.82, -1.08)	2.5 (1.9, 3.3)	2.1 (1.5, 2.9)	-1.22 (-1.59, -0.86)
Central Latin America	1.6 (1.2, 2.1)	1.4 (1.0, 1.9)	-0.39 (-0.74, -0.04)	42.5 (32.4, 56.0)	34.8 (26.1, 47.6)	-0.77 (-1.14, -0.39)	1.5 (1.0.1, 2)	1.2 (0.9, 1.7)	-0.62 (-0.97, -0.27)
Southern Latin America	0.3 (0.2, 0.4)	0.5 (0.3, 0.8)	3.05 (2.91, 3.20)	6.4 (4.6, 8.9)	11.4 (8.1, 15.7)	2.44 (2.28, 2.60)	0.2 (0.2, 0.3)	0.4 (0.3, 0.6)	2.69 (2.53, 2.86)
Tropical Latin America	0.5 (0.4, 0.6)	0.5 (0.4, 0.6)	0.29 (0.08, 0.51)	14.3 (12.2, 16.6)	12.6 (10.5, 14.8)	0.02 (-0.19, 0.24)	0.5 (0.4, 0.6)	0.4 (0.4, 0.5)	0.13 (-0.09, 0.35)

Location	Age-standardized prevalence rate among women			Age-standardized DALY rate among women			Age-standardized mortality rate among women		
	1990 No. (95% UI)	2019 No. (95% UI)	EAPC % (95% CI)	1990 No. (95% UI)	2019 No. (95% UI)	EAPC % (95% CI)	1990 No. (95% UI)	2019 No. (95% UI)	EAPC % (95% CI)
North Africa and Middle East	1.7 (1.2, 2.2)	3.9 (2.9, 5.2)	3.25 (2.90, 3.61)	43.8 (32.1, 57.4)	60.2 (44.5, 81.3)	1.39 (1.18, 1.60)	1.5 (1.1, 2.0)	2.1 (1.5, 2.9)	1.48 (1.25, 1.71)
High-income North America	0.4 (0.3, 0.5)	1.2 (0.9, 1.5)	5.28 (4.79, 5.77)	7.7 (6.6, 8.8)	17.9 (15.1, 21.2)	4.36 (3.88, 4.84)	0.3 (0.2, 0.3)	0.7 (0.5, 0.8)	4.52 (4.04, 5.01)
Oceania	2.9 (2.2, 3.8)	3.4 (2.5, 4.6)	0.70 (0.65, 0.75)	79.8 (60.6, 102.9)	91.5 (67.2, 122.7)	0.55 (0.48, 0.62)	2.7 (2.0, 3.5)	3.2 (2.3, 4.3)	0.67 (0.60, 0.74)
Central Sub-Saharan Africa	0.7 (0.4, 0.9)	0.8 (0.5, 1.2)	0.69 (0.51, 0.87)	19.9 (13.4, 27.7)	23.0 (15.0, 33.8)	0.63 (0.45, 0.81)	0.6 (0.4, 0.8)	0.7 (0.5, 1.1)	0.64 (0.45, 0.83)
Eastern Sub-Saharan Africa	0.7 (0.5, 0.9)	0.8 (0.6, 1.0)	0.22 (0.14, 0.31)	19.8 (13.8, 28.0)	22.0 (16.7, 28.8)	0.19 (0.09, 0.29)	0.6 (0.5, 0.9)	0.7 (0.5, 1.0)	0.30 (0.21, 0.40)
Southern Sub-Saharan Africa	2.5 (1.9, 3.2)	3.0 (2.4, 3.9)	0.66 (0.24, 1.08)	73.2 (57.4, 90.5)	85.0 (66.6, 108.7)	0.60 (0.20, 1.01)	2.2 (1.7, 2.9)	2.7 (2.1, 3.5)	0.67 (0.28, 1.06)
Western Sub-Saharan Africa	1.4 (1.0, 1.7)	1.6 (1.2, 2.0)	0.37 (0.29, 0.44)	38.0 (28.9, 48.3)	42.9 (31.7, 56.3)	0.29 (0.21, 0.38)	1.3 (1.0, 1.7)	1.5 (1.1, 2.0)	0.38 (0.29, 0.47)

CI confidence interval, DALY disability-adjusted life year, T2DM type 2 diabetes mellitus, EAPC estimated annual percentage change, HBV hepatitis B virus, SDI socio-demographic index, UI uncertainty interval.

Table S8. Age-standardized prevalence, DALY, and mortality rates (per 10,000,000 individuals) for liver cancer due to HBV–T2DM comorbidity in 1990 and 2019 among adults aged 60 years and over and the EAPCs from 1990 to 2019, stratified by SDI and region

Location	Prevalence rate among adults aged 60 and over			DALY rate among adults aged 60 and over			Mortality rate among adults aged 60 and over		
	1990 No. (95% UI)	2019 No. (95% UI)	EAPC % (95% CI)	1990 No. (95% UI)	2019 No. (95% UI)	EAPC % (95% CI)	1990 No. (95% UI)	2019 No. (95% UI)	EAPC % (95% CI)
**Global**	103.3 (84.8, 122.2)	119.4 (97.5, 143.4)	-0.38 (-0.80, 0.03)	2347.3 (1943.9, 2781.6)	1874 (1530.8, 2271.6)	-1.77 (-2.26, -1.29)	105.8 (88.0, 124.8)	90.0 (74.0, 108.7)	-1.53 (-1.99, -1.08)
**SDI**									
Low SDI	33.6 (24.7, 43.6)	42.6 (31.1, 55.9)	0.64 (0.50, 0.77)	825.4 (606.3, 1070.5)	1015.1 (735.0, 1341.6)	0.51 (0.37, 0.66)	37.9 (27.9, 49.1)	47.7 (35.1, 62.2)	0.61 (0.44, 0.77)
Low-middle SDI	57.9 (47.7, 69.5)	64.3 (51.7, 78.6)	-0.54 (-0.92, -0.16)	1397.6 (1156.7, 1676.4)	1442.7 (1160.6, 1769.9)	-0.80 (-1.18, -0.41)	63.0 (52.1, 75.7)	68.6 (55.1, 83.8)	-0.57 (-0.94, -0.21)
Middle SDI	203.3 (163.2, 246.2)	179.5 (145.5, 219.7)	-1.54 (-2.16, -0.91)	4749.6 (3902.0, 5762.4)	3107.4 (2509.7, 3793.5)	-2.56 (-3.15, -1.95)	212.8 (176.0, 256.9)	147.3 (120.7, 179.8)	-2.37 (-2.94, -1.80)
High-middle SDI	115.3 (95.2, 137.2)	89.9 (71.5, 109.3)	-2.16 (-2.70, -1.62)	2636.6 (2163.8, 3140.1)	1435.3 (1161.9, 1752.1)	-3.46 (-4.04, -2.88)	118.9 (98.0, 140.7)	69.3 (56.2, 83.9)	-3.20 (-3.75, -2.64)
High SDI	36.9 (29.8, 45.2)	131.0 (100.3, 164.7)	4.48 (4.02, 4.95)	627.7 (504.5, 771.8)	1180.4 (920.0, 1468.5)	2.03 (1.58, 2.48)	29.1 (23.4, 35.7)	60.0 (46.6, 74.7)	2.43 (1.98, 2.88)

Location	Prevalence rate among adults aged 60 and over			DALY rate among adults aged 60 and over			Mortality rate among adults aged 60 and over		
	1990 No. (95% UI)	2019 No. (95% UI)	EAPC % (95% CI)	1990 No. (95% UI)	2019 No. (95% UI)	EAPC % (95% CI)	1990 No. (95% UI)	2019 No. (95% UI)	EAPC % (95% CI)
**Regions**	85.8 (70.2, 102.9)	265.1 (202.9, 336.8)	3.27 (2.76, 3.78)	1254.8 (1032.3, 1504.0)	1912.0 (1499.7, 2346.8)	0.62 (-0.13, 1.38)	57.2 (47.3, 68.1)	99.3 (77.5, 121.1)	1.18 (0.46, 1.91)
High-income Asia Pacific									
Central Asia	16.7 (10.4, 24.7)	75.0 (47.3, 112.3)	4.96 (4.30, 5.62)	407.3 (251.4, 598.4)	1773.8 (1108.2, 2661.8)	4.81 (4.15, 5.48)	18.1 (11.5, 26.6)	79.5 (50.8, 119.0)	5.05 (4.35, 5.75)
East Asia	350.2 (284.1, 425.7)	204.8 (165.7, 250.4)	-3.31 (-3.94, -2.67)	8131.3 (6666.2, 9833.8)	3337.1 (2697.2, 4085.9)	-4.53 (-5.18, -3.87)	363.7 (298.8, 439.2)	159.0 (129.5, 194.7)	-4.32 (-4.95, -3.68)
South Asia	23.6 (17.8, 30.0)	36.1 (27.6, 45.3)	1.43 (1.25, 1.61)	574.8 (435.3, 726.3)	837.6 (644.8, 1065.6)	1.22 (1.03, 1.40)	25.9 (19.6, 32.6)	40.0 (31.0, 50.8)	1.43 (1.24, 1.63)
Southeast Asia	59.9 (43.5, 79.2)	109.2 (73.5, 154.5)	2.07 (1.99, 2.15)	1414.5 (1024, 1879.5)	2287.8 (1547, 3248.0)	1.64 (1.53, 1.74)	64.8 (47.2, 84.6)	108.0 (72.8, 152.3)	1.80 (1.69, 1.90)
Australasia	6.2 (4.0, 9.3)	24.4 (15.1, 37.0)	4.63 (3.94, 5.34)	119.3 (75.5, 180.3)	354.4 (227.2, 535.4)	3.57 (3.10, 4.05)	5.6 (3.6, 8.4)	17.9 (11.6, 26.9)	3.78 (3.24, 4.32)
Caribbean	69.0 (44.8, 100.2)	47.3 (29.6, 70.2)	-1.20 (-2.05, -0.33)	1559.5 (1011.8, 2281.9)	1023.0 (632.4, 1524.3)	-1.39 (-2.29, -0.49)	76.6 (49.0, 111.8)	50.3 (31.5, 73.7)	-1.37 (-2.27, -0.47)
Central Europe	38.6 (27.4, 52.0)	34.2 (22.2, 50.1)	-0.17 (-0.53, 0.18)	897.0 (635.4, 1212.6)	707.7 (461, 1041.1)	-0.66 (-1.03, -0.29)	42.6 (30.5, 56.9)	34.9 (22.9, 51.1)	-0.47 (-0.85, -0.09)
Eastern Europe	5.7 (4.5, 7.1)	13.3 (10.1, 17.0)	3.36 (3.06, 3.67)	134.0 (105.7, 168.7)	291.6 (219.6, 381.9)	3.04 (2.73, 3.35)	6.1 (4.8, 7.5)	13.8 (10.6, 17.6)	3.30 (2.93, 3.68)

Location	Prevalence rate among adults aged 60 and over			DALY rate among adults aged 60 and over			Mortality rate among adults aged 60 and over		
	1990<br>No. (95% UI)	2019<br>No. (95% UI)	EAPC<br>% (95% CI)	1990<br>No. (95% UI)	2019<br>No. (95% UI)	EAPC<br>% (95% CI)	1990<br>No. (95% UI)	2019<br>No. (95% UI)	EAPC<br>% (95% CI)
Western Europe	17.9 (12.7, 24.9)	46.3 (30.4, 68.7)	3.19 (2.90, 3.49)	326.6 (229.1, 455.2)	539.6 (357.9, 777.7)	1.51 (1.35, 1.66)	15.8 (11.1, 22.1)	28.3 (19.0, 41.3)	1.84 (1.66, 2.02)
Andean Latin America	56.7 (40.1, 74.3)	56.1 (37.2, 77.6)	-0.44 (-0.81, -0.07)	1316.4 (934.5, 1717.0)	1225.6 (811.5, 1684.5)	-0.63 (-1.02, -0.24)	64.3 (45.2, 84.4)	63.0 (42.0, 87.8)	-0.39 (-0.76, -0.03)
Central Latin America	30.5 (19.9, 44.9)	31.6 (20.9, 46.7)	0.19 (-0.14, 0.52)	706.5 (460.1, 1043.0)	691.9 (462.1, 1026.2)	-0.09 (-0.44, 0.26)	33.3 (21.9, 49.2)	33.8 (22.8, 49.0)	0.05 (-0.28, 0.38)
Southern Latin America	8.3 (5.1, 13.0)	20.9 (12.0, 34)	3.72 (3.55, 3.89)	189.1 (114.5, 297.2)	428.3 (259.6, 663.7)	3.36 (3.19, 3.53)	9.1 (5.5, 14.3)	21.6 (13.1, 33.5)	3.52 (3.35, 3.68)
Tropical Latin America	12.9 (10.2, 16.2)	17.1 (13.5, 21.4)	1.54 (1.28, 1.80)	302.8 (239.0, 381.6)	379.5 (296.9, 476.8)	1.36 (1.10, 1.61)	14.0 (11.2, 17.4)	18.3 (14.6, 22.7)	1.51 (1.25, 1.76)
North Africa and Middle East	50.7 (35.0, 69.4)	91.2 (62.0, 128.4)	2.36 (2.14, 2.57)	1194.9 (817.2, 1629.7)	1742.5 (1174.9, 2490.2)	1.61 (1.43, 1.79)	54.2 (37.3, 73.9)	81.3 (55.7, 113.9)	1.74 (1.58, 1.89)
High-income North America	12.0 (9.7, 14.8)	47.5 (34.8, 62.9)	6.17 (5.72, 6.63)	202.9 (163.3, 250.9)	607.5 (464.0, 787.2)	5.22 (4.77, 5.67)	10.2 (8.3, 12.4)	29.3 (22.9, 37.2)	4.96 (4.54, 5.38)
Oceania	72.4 (49.9, 97.1)	102.8 (71.6, 139.4)	1.31 (1.25, 1.37)	1773.1 (1224.6, 2371.1)	2455.2 (1706.7, 3345.4)	1.23 (1.16, 1.29)	79.1 (54.9, 106.5)	111.0 (77.3, 149.4)	1.24 (1.15, 1.32)
Central Sub-Saharan Africa	14.8 (8.8, 23.4)	16.5 (9.3, 27.3)	0.32 (0.18, 0.46)	375.5 (224.4, 593.2)	404.8 (226.8, 675.7)	0.19 (0.05, 0.32)	16.5 (10.0, 26.0)	18.0 (10.4, 29.2)	0.24 (0.07, 0.41)
Eastern Sub-Saharan Africa	14.5 (9.5, 21.5)	19.8 (13.2, 28.4)	0.84 (0.61, 1.07)	363.1 (237.2, 538.2)	482 (320.9, 694.1)	0.75 (0.51, 1.00)	16.4 (11.0, 24.2)	22.2 (14.9, 31.7)	0.84 (0.59, 1.09)

Location	Prevalence rate among adults aged 60 and over			DALY rate among adults aged 60 and over			Mortality rate among adults aged 60 and over		
	1990<br>No. (95% UI)	2019<br>No. (95% UI)	EAPC<br>% (95% CI)	1990<br>No. (95% UI)	2019<br>No. (95% UI)	EAPC<br>% (95% CI)	1990<br>No. (95% UI)	2019<br>No. (95% UI)	EAPC<br>% (95% CI)
Southern Sub-Saharan Africa	55.0 (32.1, 98.4)	86.6 (66.1, 111.2)	0.98 (0.30, 1.66)	1293.3 (754.5, 2330.7)	2049.2 (1559.9, 2644.2)	1.01 (0.19, 1.84)	60.3 (35.5, 107.4)	94.6 (72.5, 121.2)	0.96 (0.12, 1.80)
Western Sub-Saharan Africa	49.6 (36.3, 63.1)	64.4 (46.3, 83.6)	0.75 (0.59, 0.92)	1201.0 (890.0, 1546.2)	1528.8 (1112.1, 1980.9)	0.68 (0.51, 0.85)	56.6 (42.1, 72.2)	73.2 (53.4, 94.9)	0.76 (0.56, 0.96)

CI confidence interval, DALY disability-adjusted life year, T2DM type 2 diabetes mellitus, EAPC estimated annual percentage change, HBV hepatitis B virus, SDI socio-demographic index, UI uncertainty interval.

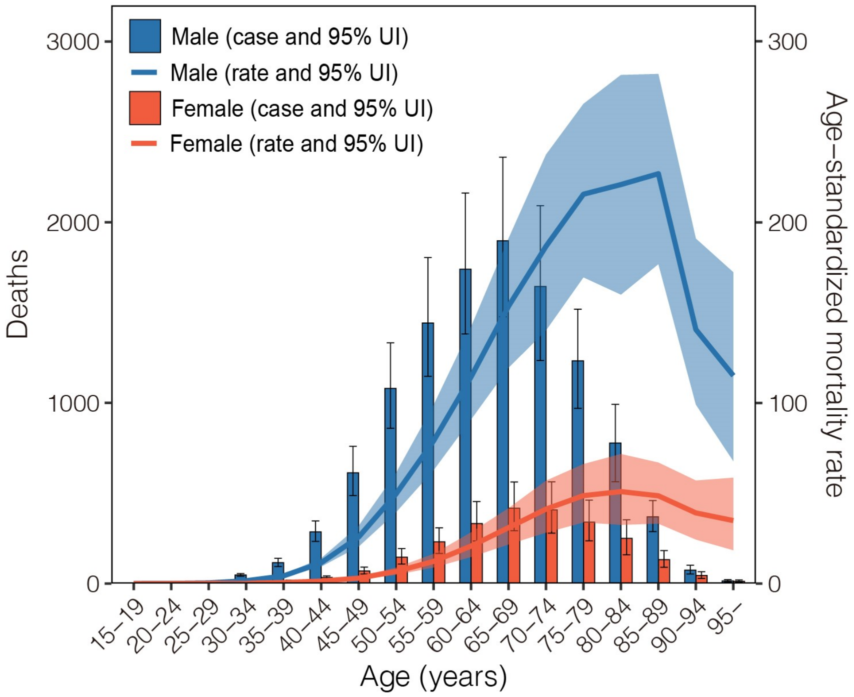

Figure S6. The global numbers and age-standardized rates (per 10,000,000 individuals) for the deaths of liver cancer due to HBV–T2DM comorbidity in 2019, stratified by age and sex. HBV hepatitis B virus, T2DM type 2 diabetes mellitus

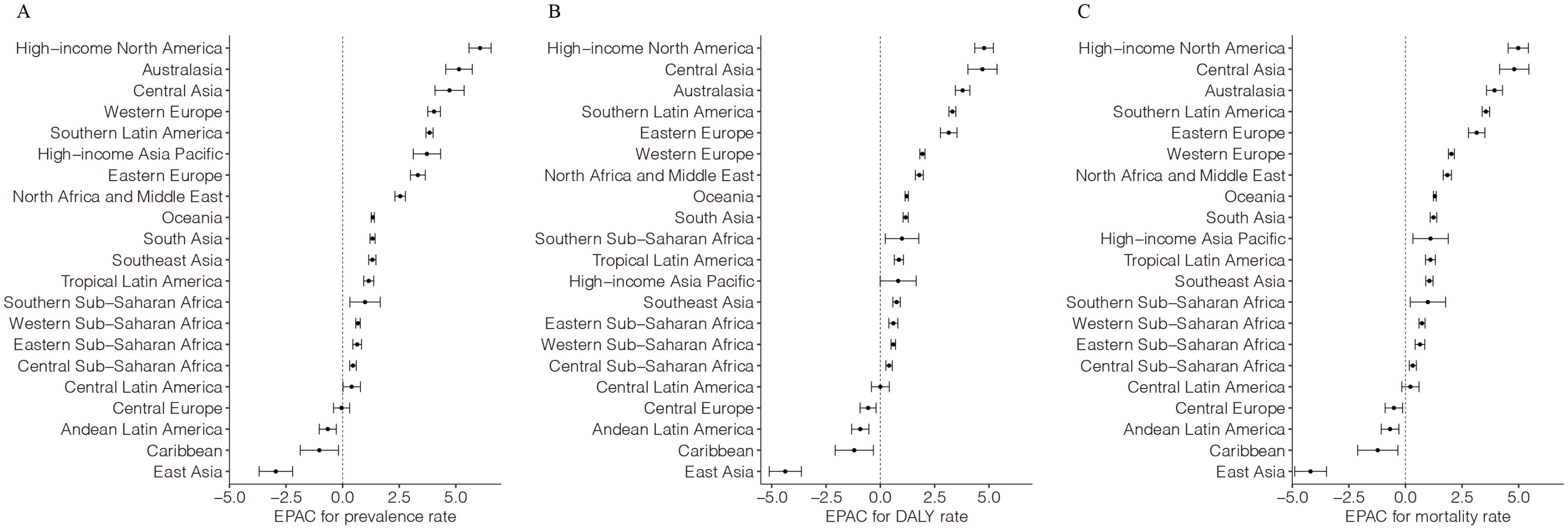

Figure S7. EAPCs of the age-standardized prevalence (A), DALY (B), and mortality (C) rates (per 10,000,000 individuals) of liver cancer due to HBV–T2DM comorbidity from 1990 to 2019 for the 21 GBD regions. ASR age-standardized rate, DALY disability-adjusted life year, EAPC estimated annual percentage change, GBD global disease of burden, HBV hepatitis B virus, T2DM type 2 diabetes mellitus

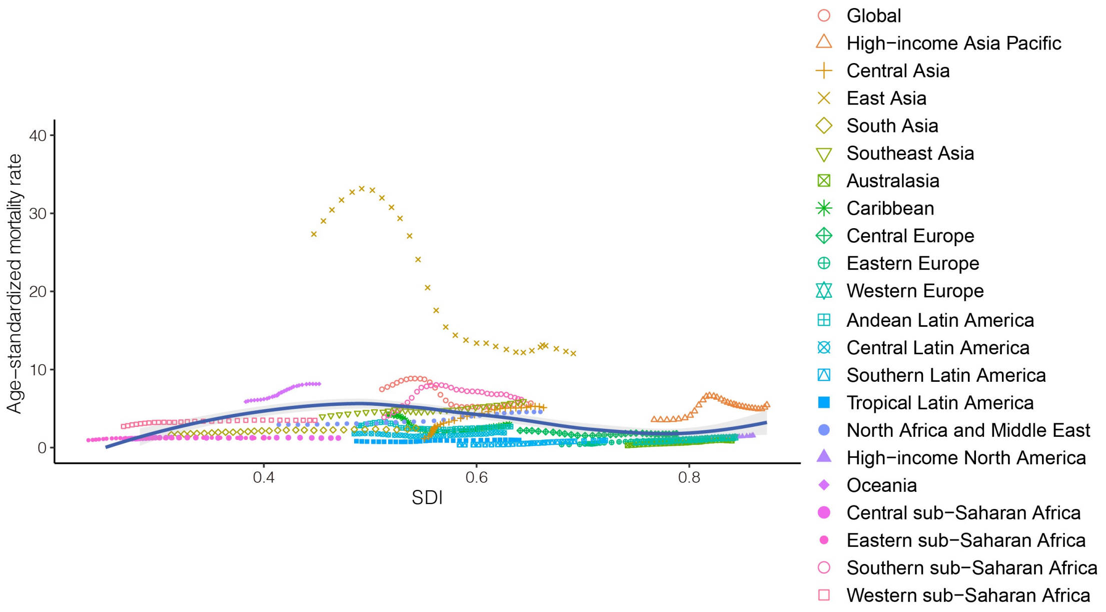

Figure. S8. The age-standardized mortality rates (per 10,000,000 individuals) of liver cancer due to HBV–T2DM comorbidity in the 21 GBD regions by SDI during 1990–2019. Each dot represents the disease burden for a year in that region. The blue line, a locally weighted scatterplot smoothing smoother, presents the expected global values based on the SDI values. GBD global disease of burden, HBV hepatitis B virus, SDI sociodemographic index, T2DM type 2 diabetes mellitus

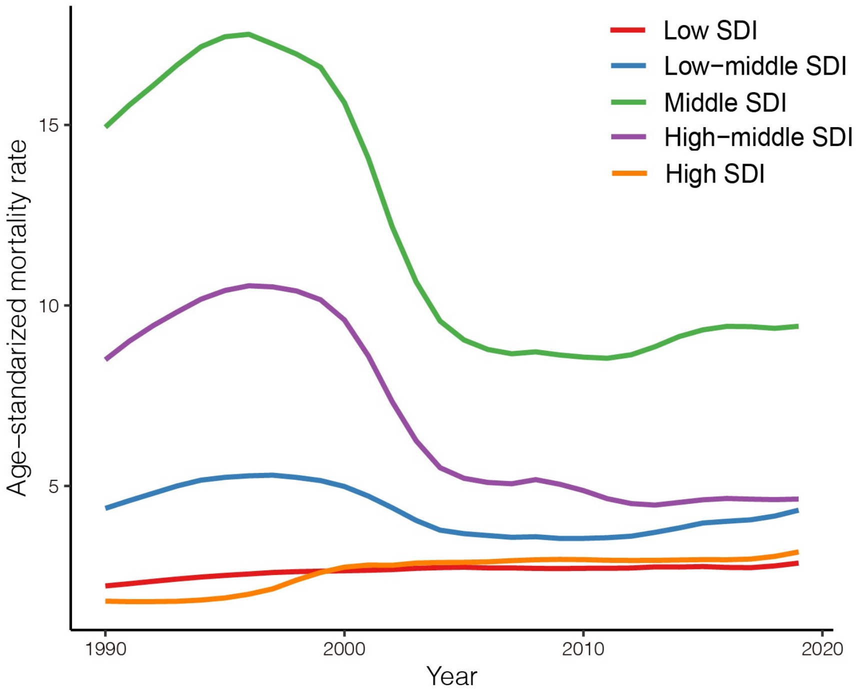

Figure S9. The trends in the age-standardized mortality rates (per 10,000,000 individuals) of liver cancer due to HBV–T2DM comorbidity from 1990 to 2019, stratified by the SDI level. HBV hepatitis B virus, T2DM type 2 diabetes mellitus

Reference

1. World Health Organization. Hepatitis B. Available from: https://www.who.int/news-room/fact-sheets/detail/hepatitis-b. Accessed 10 Apr 2024.
2. Trépo C, Chan HL, Lok A. Hepatitis B virus infection. Lancet. 2014 Dec 6;384(9959):2053-63. doi: 10.1016/S0140-6736(14)60220-8.
3. Institute for Health Metrics and Evaluation. Diabetes mellitus type 2 — Level 4 cause. Available from: https://www.healthdata.org/results/gbd_summaries/2019/diabetes-mellitus-type-2-level-4-cause. Accessed 10 Apr 2024.
4. Institute for Health Metrics and Evaluation. Liver cancer — Level 3 cause. Available from: https://www.healthdata.org/results/gbd_summaries/2019/liver-cancer-level-3-cause. Accessed 10 Apr 2024.
5. Campbell C, Wang T, McNaughton AL, et al. Risk factors for the development of hepatocellular carcinoma (HCC) in chronic hepatitis B virus (HBV) infection: a systematic review and meta-analysis. J Viral Hepat. 2021 Mar;28(3):493-507. doi: 10.1111/jvh.13452.
6. Tan Y, Wei S, Zhang W, et al. Type 2 diabetes mellitus increases the risk of hepatocellular carcinoma in subjects with chronic hepatitis B virus infection: a meta-analysis and systematic review. Cancer Manag Res. 2019 Jan 14;11:705-713. doi: 10.2147/CMAR.S188238.
